# The Spirit of Cachaça Production: An Umbrella Review of Processes, Flavour, Contaminants and Quality Improvement

**DOI:** 10.3390/foods12173325

**Published:** 2023-09-04

**Authors:** Nicolas Ratkovich, Christian Esser, Ana Maria de Resende Machado, Benjamim de Almeida Mendes, Maria das Graças Cardoso

**Affiliations:** 1 Department of Chemical and Food Engineering, Universidad de los Andes, Bogotá 111711, Colombia; 2 Wineschool3, P.O. Box 11227, Grand Cayman KY1-1008, Cayman Islands; wine@wineschool3.com; 3 Departamento de Química, Centro Federal de Educação Tecnológica de Minas Gerais, Avenida Amazonas, 5253, Nova Suiça, Belo Horizonte 30421-169, MG, Brazil; anamariaderesendemachado@gmail.com; 4 Instituto Politécnico da Bahia-IPB, Salvador 40170-140, BA, Brazil; benjaalmendes@gmail.com; 5 Department of Chemistry, University of Lavras (UFLA), Campus Universitário, Lavras 37200-900, MG, Brazil; mcardoso@ufla.br

**Keywords:** cachaça, distillation, yeast, fermentation, ethyl carbamate, sugarcane spirits, ageing, phenolic compounds, copper, chemical composition

## Abstract

This review provides a comprehensive analysis of the production, classification, and quality control of cachaça, a traditional Brazilian sugarcane spirit with significant cultural importance. It explores the fermentation and distillation of sugarcane juice, the ageing process in wooden containers, and the regulatory aspects of cachaça labelling. It emphasises the role of quality control in maintaining the spirit’s integrity, focusing on monitoring copper levels in distillation stills. Ethyl carbamate (EC), a potential carcinogen found in cachaça, is investigated, with the study illuminating factors influencing its formation and prevalence and the importance of its vigilant monitoring for ensuring safety and quality. It also underscores the control of multiple parameters in producing high-quality cachaça, including raw material selection, yeast strains, acidity, and contaminants. Further, the impact of ageing, wood cask type, and yeast strains on cachaça quality is examined, along with potential uses of vinasse, a cachaça by-product, in yeast cell biomass production and fertigation. A deeper understanding of the (bio)chemical and microbiological reactions involved in cachaça production is essential to facilitate quality control and standardisation of sensory descriptors, promoting global acceptance of cachaça. Continued research will address safety concerns, improve quality, and support the long-term sustainability and success of the cachaça industry.

## 1. Introduction

Cachaça, known as the “Mother of Rum”, is a traditional Brazilian alcoholic beverage distilled from fermented sugarcane juice. Recognised for its unique sensory characteristics deeply influenced by the terroir and the sugarcane production process, cachaça stands as the second most-consumed spirit in Brazil and the third globally [1]. Its close relative, rum, is derived from the alcoholic fermentation and distillation of sugarcane molasses, syrups, juices, or cane sugars generated during sugarcane processing. However, in particular Caribbean islands, e.g., Martinique and Haiti, rum is made from sugarcane juice, such as cachaça. The connection between cachaça and rum traces back to the historical growth of the sugar industry, where cachaça played a significant role [2].

Despite cachaça’s global popularity and status as a protected geographical indication (GI) product by the Brazilian Ministry of Agriculture, Livestock, and Supply (MAPA) as of 2023 [3], concerns linger around the quality and safety of its production. Challenges and potential contaminants persist through the stages of fermentation, distillation, and ageing, posing a threat to the overall quality of the final product.

This review examines these production stages in-depth, identifies potential contaminants, and offer insights into high-quality cachaça production [4]. It sets out to build upon the existing body of review literature surrounding cachaça production and quality control, adding a fresh perspective and innovative approach to tackling persistent issues in the field. In particular, the review will underscore the importance of adhering to the MAPA guidelines, which stress that cachaça should be exclusively produced from fermented sugarcane juice, as a crucial step in ensuring public health and maintaining the integrity of this beloved spirit.

Cachaça is a unique and exotic alcoholic beverage that has been intricately linked to the history of Brazil. It was introduced by the Portuguese between 1532 and 1548 when they brought the sugarcane plant to Brazil, originally from South Asia. An alcoholic drink was produced from the sugarcane syrup, sour garapa, which later became cachaça [5]. In 2001, the publication of Decree 4062/2001 defined the expressions “cachaça,” “Brazil,” and “Brazilian cachaça” as GI. This decree, the result of the work carried out by the Brazilian Program for the Development of Cane Spirit, Caninha or Cachaça (PBDAC), is a milestone for cachaça and is the primary legal instrument that has been used as the basis for the defence and protection of cachaça internationally.

Historically, cachaça was viewed with disdain as it was considered the liquor of the poor and enslaved, similar to South America’s moonshine. Even words related to cachaça carried negative connotations; in Portuguese, a person who makes cachaça was called an Alambiqueiro instead of a Cachaceiro, as the latter term was a slur used to describe a drunkard. Despite this negative reputation, cachaça has a long history and was created a century before rum. Not until the late 20th century did cachaça’s reputation shift positively. Today, there is a growing appreciation for cachaça as a high-quality spirit, and it is increasingly recognised for its unique flavour and cultural significance. Cachaça’s growing recognition is its use in mixed drinks, such as the caipirinha, Brazil’s de facto national drink (see Appendix A). While the caipirinha is a well-known cocktail, the flavour of cachaça can be easily lost in the mix. This may have contributed to cachaça’s relative obscurity outside of Brazil. However, with a greater awareness of cachaça’s unique qualities, it is gaining recognition as a distinct and essential spirit in the spirits world [6].

In 2012, Colombia became the first country to recognise cachaça as a distinct Brazilian product, marking a turning point in the protection and recognition of cachaça internationally. In 2013, the glory of cachaça in the US market broke years of product generalisation—prior to this, it had been forcefully labelled “Brazilian Rum” from 2001 to 2013 for sale in the United States. The recognition allowed cachaça to be marketed as cachaça and was an immeasurable gain for Brazil, with Brazilian producers’ exclusive use of the denomination cachaça. In 2016, the Agreement of Mutual Recognition of Cachaça and Tequila as Geographical Indications and Distinctive Products of Brazil and Mexico officially concluded negotiations to recognise cachaça in Mexico as a Brazilian-exclusive distilled spirit. The negotiations between the two countries had been ongoing for several years. Still, the joint actions of the Brazilian Institute of Cachaça (IBRAC) and the Tequila Regulatory Council (CRT), starting in June 2014, strengthened the process of reciprocal recognition.

Finally, in 2018, Chile recognised cachaça as a distinct Brazilian product through the Free Trade Agreement between Brazil and Chile, which allowed for mutual recognition of geographical indications of cachaça and pisco (Chilean). This recognition added to the growing international recognition of cachaça as a unique Brazilian product with a rich history and cultural significance.

The national production of cachaça is estimated to be 800 million litres per year, with 70% of the production represented by industrial cachaça and 30% by alembic cachaça. The use of copper alembic stills is traditional in the production of cachaça and is required for cachaça to be classified as such [7].

Produced exclusively in Brazil, cachaça is a distilled alcoholic beverage—or aguardente de cana—with an alcohol content ranging from 38% to 48% ABV at 20 °C [8]. Cachaça is produced from the fermented juice of sugarcane and then distilled in a copper alembic still. Although not required, sugars may be added to cachaça. It is essential to underscore that cachaça is differentiated from rum—predominantly produced in Caribbean countries—because it is made from the distillation of fermented raw sugarcane juice. In contrast, rum is produced from fermented cooked sugarcane juice and molasses [9,10].

### 1.1. Classification and Labelling

The classification and labelling terminology for cachaça is summarised in Table 1. It is essential to highlight that labelling is enforced by Brazilian legislation.

In addition to the classification presented in Table 1, the labelling must comply with the specific technical regulations related to packaged foods [4].

- The relative ageing time may only be declared for cachaça, aged entirely for a period equal to or greater than one year.
- In the case of mixtures between aged products, the declaration of ageing time on the label shall correspond to the product with the shortest ageing period.
- In the labelling of cachaça, as long as it is dissociated from the denomination, the terms of:
  ○ “Premium” may be used for a beverage aged for at least one year.
  ○ “Extrapremium” may be used for a beverage that is entirely aged for at least three years.

If the cachaça is packaged with wood fragments, the label must clearly state the type and name of the wood used. The label does not allow any association with the ageing process or classification of aged cachaça. Additionally, the name(s) of the wood(s) used for storage or ageing must be included on the label clearly and prominently [4].

Certain superlative expressions, such as artisanal or natural, are prohibited on the label unless federal legislation allows it. The label must also declare any added sugar or caramel colouring in the ingredient list. Adhering to these labelling regulations ensures transparency and provides consumers important information about the cachaça they purchase [4].

### 1.2. Overview of the Production Process

The production of high-quality cachaça can be influenced by various factors such as raw materials, fermentation, distillation, and ageing. Research has been conducted throughout the entire cachaça production chain, including sugarcane varieties, fermentative aspects, yeast strain selection, distillation process improvement, choice of woods for ageing the finished product, and chemical analysis to characterise the high-quality product. The fermentation process is considered the critical point in the manufacturing process as several compounds that form the aroma characteristic of the beverage are formed during this stage.

**Figure 1:**
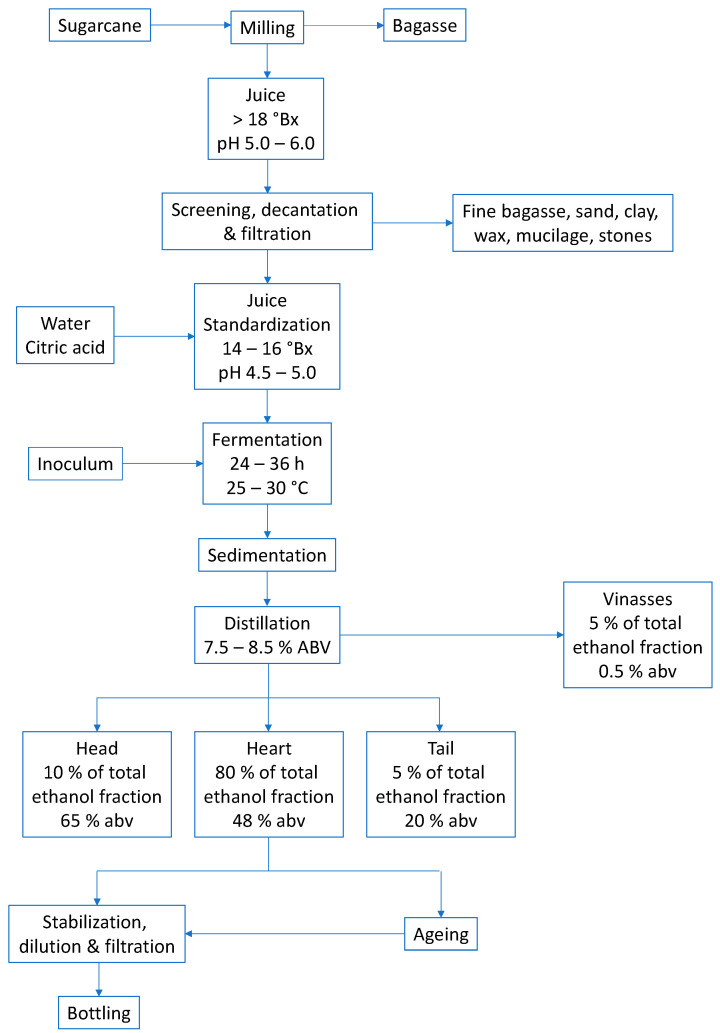
Cachaça production process.

The production processes of alembic and industrial cachaça present distinct characteristics that result in different product profiles. Alembic cachaça, as described, is produced through meticulous steps, including sugarcane selection and preparation, fermentation, distillation, and ageing. These measures ensure a good yield and exceptional product quality (Figure 1). Each stage contributes to the final sensory profile of the cachaça, with the ageing process adding particular depth and complexity.

On the other hand, industrial cachaça production involves a more streamlined and large-scale approach, often using column stills for continuous distillation. The distillation process in industrial production tends to be faster. While it can achieve greater volume, it may not always capture the same depth of flavour and complexity as alembic cachaça. Industrial cachaça often skips the ageing process, which can limit the depth and range of its flavour profile.

Despite these differences, both types of cachaça hold their unique value and appeal. Alembic cachaça is often prized for its artisanal quality and depth of flavour, while industrial cachaça is appreciated for its accessibility and consistent product output. Recognising these variations is crucial in understanding the diverse landscape of cachaça production. Appendix B provides further details on the basic calculations related to these processes.

Figure 1 shows the flowchart of the alembic cachaça production process. Each stage defines the ideal parameters for good beverage yield, ensuring it meets legal standards and is safe for the consumer.

#### 1.2.1. Preparation of the Sugarcane

The sugarcane harvest plays a critical role in determining the quality of the final product. Sugarcane stalks, the primary raw material, should possess specific characteristics before being utilised for cachaça production [11].

- Composition: sugarcane consists chiefly of two primary elements: fibre and juice. The fibre component constitutes approximately 8 to 14% of the cane and includes compounds such as cellulose, lignin, pentosan, and cane gum. Conversely, the juice represents a more substantial proportion, between 86 and 92% of the cane. This juice component predominantly consists of water, accounting for 75 to 82% of its composition. Within the juice, soluble solids are present in a range of 18 to 25%. Sugars, a significant component of these soluble solids, make up 15 to 24% and can be further classified into saccharose (14 to 24%), glucose (0.2 to 1.0%), and fructose (0.2 to 1.0%). Beyond sugars, soluble solids comprise non-sugars, representing 1 to 2.5% of the composition. These non-sugars are categorized into organics and inorganics. The organics, which make up 0.8 to 1.5%, encompass amino acids, nitric acid, fats and waxes, and nitrogenated material. In contrast, inorganic substances constitute 0.2% to 0.7% of the soluble solids, featuring compounds such as SiO_2_, K_2_, P_2_O_5_, CaO, Na_2_O, MgO, Cl, and Fe_2_O_3_ [12].
- Maturity: sugarcane is considered mature when Brix (% soluble solids or °Bx) ≥ 18 °Bx, Pol (% apparent sucrose) ≥ 14.4%, juice purity ≥ 80%, and reducing sugars (glucose and fructose) ≤ 1.4%. Field refractometers and densitometers can be used to determine the maturity index (MI) and Brix levels.
- Freshly cut: stalks should be processed within 36 h of cutting to ensure freshness. However, this interval may extend to 72 h in specific regions during the beginning of the harvest season.
- Topped stalks: sugarcane tops, low in sugar and containing components that can interfere with fermentation, should be removed before milling.
- Minimal foreign matter: the presence of vegetable or mineral impurities can compromise the quality of industrial sugarcane. Proper cane topping, lateral stripping, and weed control are essential, and minimal mineral impurities are ensured, especially for cachaça production.
- Good health: the quality of the raw materials is affected by the health of the stalks. The borer-rot complex can depreciate the quality of sugarcane, reducing sugar content and the purity of the raw material. Borer infestation rates up to 3% are considered normal.

Harvest operation: sugarcane harvesting should be conducted without prior burning of the cane field to avoid sugar loss, increased impurities, and microbial contamination. Cane cutting should be performed close to the ground level, and topping must be carried out to prevent clogging in the mill and reduce cachaça yield.

Proper harvesting techniques and attention to these factors are crucial in obtaining a high-quality final cachaça product. To ensure the quality of cachaça, it is essential to consider the cutting method of sugarcane. Manual cutting is the most frequently used method by cachaça producers, as mechanized harvesting can cause issues in sugarcane processing due to increased dirt, such as soil, residue from corrosion and abrasion originating from the equipment. During the harvest, producers must pay attention to hygiene during cutting and transportation to the milling process [11].

The ideal harvesting point for sugarcane is crucial as it determines the yield of the extraction process. The sugar content of each internode should be measured separately to calculate the MI. Sugarcane is considered ripe for milling when the MI is equal to or greater than 0.85 and less than 1.00. Apart from the recommended instruments for determining the sugar content of the juice, producers can use other laboratory resources, such as a refractometer, to determine the suspending solids as brix degrees, which should be between 20 and 22 °Bx, and the pH of the sugarcane juice, which should range from 5.0 to 6.0 [11].

Delays in the transport and processing of cut sugarcane, especially during humid and hot periods, can result in the loss of sugars, favouring the formation of fermentative yeasts and the possibility of microbial deterioration. It is essential to process sugarcane within 24 h after the harvest. Adjusting the milling machine and the bagasse position is also fundamental to the success of the milling operation. The speed of the cylinders is a function of the opening adjustment, the cylinder’s compression, and the peripheral speed, which mainly determines the milling capacity. The peripheral speed limit in Brazil is 20 m/min due to the materials’ resistance and the negative effect on sugar extraction. The types of milling grooves utilised are usually transverse circumferences and drainage channels. Nowadays, there are variations in the groove models to improve cane juice extraction after extracting the sugarcane juice by crushing the sugarcane in mills, which is then filtered and decanted to eliminate impurities [11].

#### 1.2.2. Fermentation Process Parameters

The traditional process of producing cachaça involves two main stages: propagation of microorganisms and fermentation [5,13,14,15]. In the first stage, microorganisms’ propagation occurs under intense aeration with a sugar content close to 5 °Bx. Higher concentrations can inhibit cell respiration, which is crucial for efficient growth. Most distilleries use homemade yeast or inoculated pressed yeasts used in baking. The second stage, fermentation, requires the addition of juice with a sugar content between 14 and 16 °Bx [8,14]. Sugar concentrations above 16 °Bx can result in slower and incomplete fermentations [5], reducing the yield and quality of the final product.

The fermentation process consists of three phases: initial (pre-fermentation), primary (tumultuous fermentation), and final (post-fermentation). The initial phase requires oxygen for yeast multiplication, while the primary fermentation produces enzymes that convert sugars into alcohol and CO_2_, forming bubbles in the must. The final stage ends with the complete stoppage of CO_2_ release, the disappearance of bubbles, and the return of the temperature to ambient temperature, with the stabilisation of the pH value. The pH value and the temperature of the must during fermentation should be controlled to prevent the development of contaminating microorganisms. Bacterial contamination may occur when pH values are close to 4 and the temperature is above 32 °C [11].

To create the right environment for yeast fermentation, a ferment propagator is needed for optimal yeast propagation and the development of desirable microorganisms. The propagator provides an exclusive aerobic metabolism that results in lower ethanol formation and higher production of yeast cells. To ensure efficient control of fermentation, constant low sugar levels, the balance of other nutrients, and control over temperature and respiratory quotient are necessary. The propagator can be made available in the fermentation tank with an air compressor or agitator to bubble the existing ferment [11].

Fermentation tanks should be made of non-reactive materials resistant to repeated cleaning and sanitation operations. They are usually cylindrical with a diameter equal to half the height, but broader vats are used mechanically when yeast recovery is made. The bottom is cone-shaped, with a central drain channel. Nowadays, fermentation tanks with a more inclined bottom are preferred for easier liquid drainage and surface cleaning. Mechanical agitation can be beneficial when using fermented yeast from a propagator, but over-agitation can damage the yeast. Starchy materials and technology adjuvants are permitted, but adding any ingredient not provided for in the regulations or that can be used to adulterate the product is prohibited [11].

Factors affecting alcoholic fermentation include aeration, sugar concentration, pH, temperature, and contaminating microorganisms. Meeting the nutritional requirements of yeast, such as minerals, vitamins, and unsaturated fatty acids, is essential since these nutrients are often insufficient in sugarcane [11]. By providing yeast with these nutrients, the yield of alcoholic fermentation and the final ethanol concentration can be increased.

Yeast requires oxygen during the oxidative metabolism phase, which occurs in the presence of oxygen, to stimulate intense multiplication. However, without oxygen, the metabolism becomes fermentative, producing ethanol and CO_2_. Sugar concentration affects the production of cellular yeast biomass and the fermentation process, with well-diluted broth during the yeast multiplication phase and gradually increasing sugar concentration as the cellular mass reaches an appropriate level. The optimal range for fermentation occurs at a pH of 4.5, and the ideal temperature for fermentation is around 25–30 °C. Yeast can also demand nitrogen during the cell multiplication phase [11].

Using non-sterile sugarcane juice as a substrate can introduce a diverse population of microorganisms into the fermentation vessel, interfering with fermentation and producing undesirable by-products [3]. To avoid such issues, discarding contaminated yeast populations, cleaning the vessel thoroughly with hot water, and preparing a new yeast starter culture for the subsequent fermentation cycle is crucial. The microbiome of traditional cachaça fermentations is complex and includes a variety of yeast genera and bacteria, such as *Lactobacillus* and *Lactococcus* spp. However, using specified yeast strains can help avoid fluctuations in fermentation performance and congener formation, which can affect the safety and sensory characteristics of the spirit and fail to comply with Brazilian identity and quality standards [11].

The initial inoculum volume should be 12 to 20% of the fermentation vat volume, depending on the inoculum quality [8] (see Appendix C). The fermentation process can vary from 20 to 36 h, but on average, it is 24 h [5,14,16]. The fermentation progress is monitored by observing the drop in °Bx, which should occur every 1 or 2 h. The decrease in °Bx in the must indicates glucose utilisation, likely being converted into alcohol. The total dissolved solids contribute to the specific gravity, which decreases when sugar is converted into alcohol and CO_2_.

During fermentation, volatile congeners are produced, and excessive formation can be controlled using preventive measures. The content of each volatile congener is measurable only in the final spirit, and monitoring procedures are generally not applicable during fermentation. Therefore, corrective actions are typically taken during the distillation process. Preventive measures to avoid acetic acid formation include minimising acetic bacteria contamination during and after the fermentation step. Esters and aldehydes are essential components of sensory characteristics in spirits, and their production depends on the relative abundance of the corresponding alcohols and acyl-coA radicals involved in yeast metabolism. Higher alcohols produced by yeast include n-propyl, isobutyl, and isoamyl alcohol; their presence is essential for the aromatic characterisation of cachaça. However, when in excess, they cause adverse effects. The most critical safety aspect related to fermentation is to avoid the formation of ethyl carbamate (EC) precursors, which are considered the primary contaminant of spirits since they are potentially carcinogenic compounds [2].

In summary, creating the ideal environment for yeast fermentation in cachaça production is crucial. A ferment propagator, non-reactive fermentation tanks, and controlled pH and temperature are all necessary for efficient fermentation. The fermentation process has three phases, each requiring specific conditions for optimal yeast activity. Careful attention to these details is essential for high-quality cachaça production.

#### 1.2.3. Distillation

After fermentation, the fermented must is distilled to produce cachaça. The yeast remaining in the vat can initiate a new fermentation cycle [14,15]. Distillation is purifying and concentrating the volatile compounds formed during fermentation to enhance the sensory characteristics of the raw materials. Encrustations can occur in the alembic during the distillation of the fermented juice, resulting in furfural formation and an undesirable aroma and taste in cachaça [17]. The fermented must, or wine, comprises approximately 88% to 93% water, 5% to 9% ABV, and small amounts of various alcohols, acids, esters, and aldehydes crucial to the quality of cachaça [15].

Small and medium cachaça producers typically use copper pot stills with two plates during distillation, while larger producers use column stills. The first distillation is focused on recovering ethanol from the wash, and the second distillation involves separating the distillate into “heads,” “hearts,” and “tails.” However, the “heads” fraction, which includes aldehydes, esters, and methanol, can negatively impact the quality of the final product if not controlled. Brazilian law limits the concentration of aldehydes in cachaça. Acetic acid and furfural are concentrated in the last fraction of the distillate, and their removal by cutting at about 38% ABV is essential to reduce the acidity formed in the wine. Double distillation can be a great alternative to remove excessive acetic acid from the spirit [11]. Contamination of spirits during distillation can occur, including copper contamination, and Brazilian law mandates that the maximum permitted content of copper in cachaça is 5 mg/L. Heavy metals can also contaminate cachaça and rum during the production process. Methanol is undesirable in distilled spirits because of its toxicity and is concentrated in the “heads” fraction of the distillate. Controlling distillation or applying double distillation can remove methanol effectively [2].

In the production of cachaça, separating the “head” and “heart” fractions is a critical step in distillation and is essential for producing high-quality spirits. The volatile fractions are separated through cuts in the distillate, which separate the desirable compounds from the undesirable ones found in the head. This process leads to the desired alcohol content in the heart and allows for the reuse of the tail in subsequent distillations to obtain other products. The presence of volatile compounds in the fractions of the distillates depends on their boiling point and their affinity for water, alcohol, or both. Based on this principle, the volatile compounds are classified into four groups: those that are fully or partially soluble in alcohol, those soluble in water, those soluble in both alcohol and water and those that are not soluble in alcohol but can be transported by water vapour (hydro-distillation) [11].

The “head” and the beginning of the “heart” distillation contain volatile compounds that are soluble in alcohol and have a low boiling temperature, such as acetaldehyde and ethyl acetate, entirely or partially soluble in alcohol and water and have a boiling temperature of less than 200 °C such as methanol and superior alcohols, and entirely or partially soluble in alcohol and have a high boiling temperature such as fatty acids and their esters, isoamyl acetate, ethyl hexanoate, ethyl octanoate, ethyl decanoate, and ethyl dodecanoate [11].

The middle of the “heart” and “tail” contain volatile compounds that are soluble or partially soluble in water with a boiling temperature above the boiling point of water, such as acetic acid, 2-phenyl ethanol, ethyl decanoate, and diethyl succinate. It also contains volatile compounds soluble in water and has a high boiling temperature, such as furfural. Furfural and higher alcohols, which are hydrophilic compounds, are present in all distillate fractions [11].

**Figure 2:**
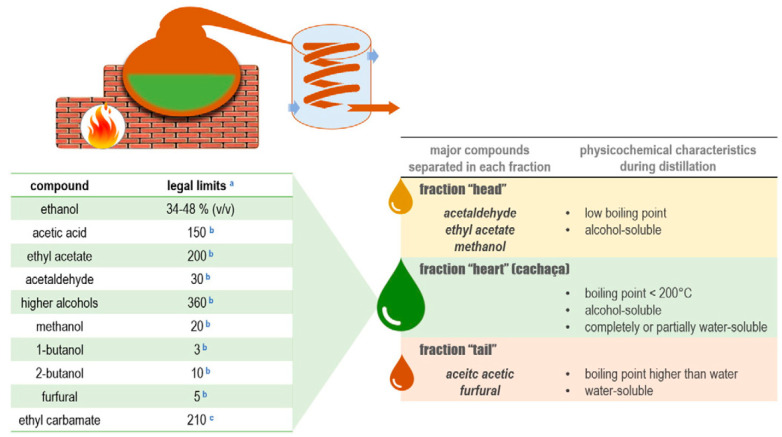
The traditional scheme of cachaça distillation and legal limits of volatile compounds: ^a^ legal limits according to Brazilian legislation; ^b^ concentrations in mg/100 mL anhydrous alcohol, and ^c^ concentration in μg/L. Reprinted with permission from Portugal et al. [18].

The process involves collecting three different distillates: the head, the heart, and the tail (Figure 2). The head contains high levels of unwanted secondary components and should be discarded, representing 1% of the fermented must or 10% of the total volume of distillate. In comparison, the heart is the desired distillate representing the volume equivalent to 16% of the total volume of the fermented must or 80% of the total volume of distillate. The tail represents 3% of the fermented must or 15% of the total volume of distillate and should be cut when the alcoholic strength of the fraction reaches 14% ABV. After distillation, cachaça produces vinasses containing minerals, sugars, non-fermenting substances, yeast cells, and soluble non-volatile or volatile acids in water and alcohol [11].

Efficient distillation requires a suitable still and an appropriate distillation technique, which depends on the still’s reflux, column geometry, and vapour condensation. For instance, certain compounds, such as furfural and hydroxymethylfurfural (HMF), are formed during distillation and not fermentation, and their presence in cachaça depends on the still and technique used. The distillation process significantly impacts the sensory characteristics of the final product. The presence and concentration of volatile components depend on the type of still and the distillation technique used. Good distillation practices, such as controlling the distillation temperature (≤90 °C) and distillation time (from 1.5 to 3 h), allowing for sedimentation or filtration of fermented must before distillation, and separating the fractions, are essential to ensure the quality of the cachaça and avoid harmful substances [11].

Figure 2 illustrates the traditional cachaça distillation process, which involves separating distilled fractions into the “head” (first 1–2% of boiler volume, discarded); the “heart” (distillate collected up to 40–45% ABV); and the “tail” (collected after the “heart” until distillation ends). The critical “head” and “tail” separation eliminates most contaminants. The “head” contains a low boiling point and alcohol-soluble compounds such as acetaldehyde, ethyl acetate, and methanol. At the same time, the “tail” has a higher boiling point and volatile water-soluble compounds such as acetic acid and furfural. The “heart” represents the cachaça, which must meet Brazilian legislation’s chemical criteria for product safety and sensory quality, summarized in the table accompanying Figure 2.

**Figure 3:**
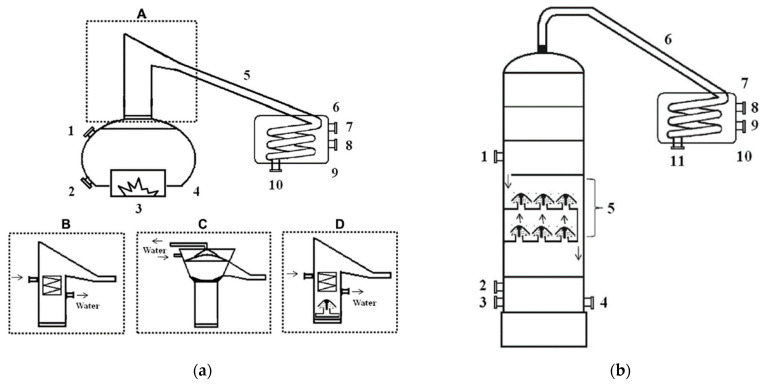
(**a**) Pot still column distiller and cooling devices. (1) Vine inlet; (2) vinasse outlet; (3) heating system; (4) kettle; (5) condenser tube; (6) condenser tank; (7) cooling water inlet; (8) heated water outlet; (9) condenser coil; (10) distillate outlet. (A) Hot head column type; (B) column with tubular dephlegmator; (C) head cooler column type; (D) column with tubular dephlegmator and bubble cap tray, and (**b**) continuous column distiller. (1) Vine inlet; (2) steam inlet; (3) steam outlet; (4) vinasse outlet; (5) reflux system with bubble cap trays; (6) condenser tube; (7) condenser tube; (8) cold water inlet; (9) heated water outlet; (10) condenser coil; (11) distillate outlet. Reprinted with permission from Riachi et al. [19].

Cachaça production can be divided into two main distillation methods: batch and continuous. Batch distillation is the traditional method that involves using copper pot stills. This method produces a small quantity of high-quality cachaça with a flavour and aroma profile influenced by the still’s size and shape and the distiller’s skill. On the other hand, continuous distillation uses industrial stainless-steel columns and produces a large quantity of cachaça in a shorter time. However, the resulting product has a more uniform taste and aroma, with less complexity and character than the alembic cachaça. The choice of distillation method is often determined by factors such as the scale of production, desired flavour profile, and economic considerations. Understanding the differences between batch and continuous distillation methods is essential for cachaça producers, as it can impact their product’s quality and commercial value (Figure 3).

Figure 3a,b present diagrams illustrating the various types of distillation equipment employed in the production of cachaça. Figure 3a presents a pot still column distiller featuring a heating system, kettle, condenser tube, and distillate outlet, with various column types such as hot head column, the column with tubular dephlegmator, head cooler column, and column with tubular dephlegmator and bubble cap tray. Figure 3b depicts a continuous column distiller, including a wine inlet, steam inlet, steam outlet, vinasse outlet, reflux system with bubble cap trays, condenser tube, cold water inlet, heated water outlet, condenser coil, and distillate outlet. The diagram demonstrates the continuous distillation system components and their interplay in producing cachaça. Both figures elucidate the diverse distillation equipment types in cachaça production and their impact on the final product’s quality and traits, offering a visual guide to the equipment and processes.

#### 1.2.4. Ageing

The production of cachaça involves an essential step of ageing the distilled product in wooden barrels to achieve specific reactions and modify its flavour profile. To meet regulatory standards, appropriate first-use wooden containers must be used for storing and ageing the raw material used in cachaça, and it is prohibited to use equipment that does not comply with technical regulations for food contact materials. Wood fragments can also be added to the cachaça for sensory enhancement, but they must meet specific requirements. Ageing in wooden barrels softens and improves the harsh characteristics of newly-distilled spirits, and the wood acts as an active packaging that modifies the beverage’s characteristics over time, enhancing its quality [11].

Brazilian law mandates that aged cachaça must contain at least 50% of the spirit matured in appropriate wooden barrels for a minimum of one year, with premium and extra-premium cachaça aged for one year and three years, respectively. While oak is the primary wood used for spirits ageing worldwide, native Brazilian woods such as amendoim, araruva, and cabreúva offer flavour characteristics due to the extraction of specific compounds. They can be a viable option for cachaça producers [11].

During ageing, the cachaça gradually develops a yellowish tint and a transparent appearance, with a typical vanilla-like aroma and a more pronounced wood flavour. After one to two years of ageing, the taste becomes even more astringent due to the tannins from the wood, with a harmonious and rounded character. At three years, the spirit’s aroma is no longer easily distinguishable from the tannins in the wood. There is a specific association between the distillate and wood-derived components, as various chemical reactions occur during maturation and ageing [11].

Secondary components from the wood, such as tannins and phenolic compounds from lignin, play a significant role in the ageing process. The ageing also involves the extraction of components from the wood, the decomposition of macromolecules from the wood, and their incorporation into the spirit, the transformation of extracted materials from the wood, the reactions of the wood components with the original distillate components, the evaporation of volatile compounds through the wooden barrel, and the formation of stable molecular complexes between the secondary compounds and water and/or ethanol [11].

The transformation of the distilled product occurs via both additive and subtractive pathways. Additive pathways involve the decomposition of macromolecules from the wood, extracting minor components, and oxidation of compounds. In contrast, subtractive pathways involve the evaporation of volatile compounds, adsorption of molecules by the wood fibres, and oxidation of compounds. During ageing in wooden barrels, there is a gradual increase in the dry extract content, with tannins and phenolic compounds from lignin comprising up to 40%. Many aldehydes and phenolic acids, such as vanillin, syringaldehyde, conifer aldehyde, and sinapaldehyde, have been identified in spirits aged in oak barrels [11].

After the ageing process, filtration and standardisation are essential steps to remove solid particles from the barrels, reduce the alcohol content, and avoid future turbidity in the spirit. Good-quality water for standardisation is recommended, and water should be filtered using specific mineral and heavy metal filters to prevent turbidity in the final product after bottling. The bottling system must be automatic, and the process must be carried out in a specific and separate room to avoid physical contamination [2].

### 1.3. Components, Contaminants, and Safety

Despite its popularity, it faces significant challenges concerning its quality and safety. Contaminants, including heavy metals, pesticides, and mould growth toxins, are a significant concern in production. Monitoring these is crucial to ensure the safety and quality of the final product. Table 2 shows the main components and contaminant limits stipulated by Brazilian legislation. Moreover, the ageing process, wood cask type, and yeast strains impact the quality and authenticity of cachaça. While the concentration of EC in cachaça is below the limit set by legislation in Brazil, further research is necessary to ensure the safety and quality of this popular drink. Recent research has explored the potential of vinasse, a by-product of cachaça production, in several applications, including yeast cell biomass and as a medium for fertigation.

A thorough understanding of quality control is essential for cachaça production, involving complex chemical processes such as fermentation, distillation, and wood maturation. The chemical reactions during these processes are complex, and understanding the thermodynamic driving forces behind these reactions is vital. The sugarcane spirits industry in Brazil is significant, generating US$ 6 billion in total revenue per year, and future research trends in cachaça production include improving quality, ensuring product safety, and utilising new technology and research for fermentation, distillation, and ageing [2].

The undesirable secondary compounds in cachaça production are: acrolein, 1-butanol, 2-butanol, copper, EC, furfural, hydroxymethylfurfural (HMF), methanol, and polycyclic aromatic hydrocarbons (PAHs) [20].

PAH limits in alcoholic beverages, including cachaça, differ globally, reflecting varied regulatory environments. For instance, the European Union imposes a legal limit of 2 μg/L for benzo[a]pyrene (BaP), indicating PAHs in food items. In Germany, the German Society for Fat Science suggests a higher threshold, a limit of 25 μg/L for total PAHs in alcoholic beverages. Specifically, concerning cachaça, a study identified that some of these spirits, produced from burned sugar cane crops, exhibited median PAH levels ranging from 2.99 × 10^−2^ to 6.19 μg/L [4]. This underscores the variance in PAH content, depending on the source material and the production process. These outlined limits are not static and may be subject to future amendments in light of evolving research and regulations. Consequently, continual monitoring and research are critical to ensuring adherence to standards and the production of safe, high-quality cachaça [21].

Acrolein is an aldehyde that can be formed during the distillation of cachaça from the dehydration of glycerol or by *lactobacillus* in the wort, which converts glycerol into β-hydroxy propionaldehyde. It is highly toxic and mutagenic, irritating the eyes, nose, and throat. In a study by Masson et al. [22], 9.85% of the analysed sugarcane spirit samples exceeded the legal limits for acrolein content established by Brazilian legislation.

Both 1-butanol and 2-butanol are bacterial byproducts that may compromise the quality of cachaça when present in high concentrations. Contamination by *Clostridium acetobutylicum* is known to cause 1-butanol formation during fermentation [11]. In studies by Bortoletto et al. [23], 1-butanol and 2-butanol were responsible for 12.5% and 380% of the irregular samples, respectively. The leading cause of these compounds in cachaça is bacterial contamination during production.

Copper, a metal used in still construction, contributes to the aroma and flavour of cachaça but can harm the human body and catalyse the formation of carcinogenic EC when present in high concentrations [24]. Brazilian legislation limits copper content to 5 mg/L in distilled beverages [4], whereas other countries enforce stricter regulations, limiting the content to 2 mg/L.

EC is a widespread contaminant in fermented food products and alcoholic beverages, classified as a Group 2A carcinogen, posing a significant risk to human health and creating challenges for sugarcane spirits export [25].

Furfural and HMF may impart an unpleasant taste and negatively impact the final product’s quality. Brazilian legislation does not establish limits for furfural, and HMF in sugarcane spirits, but their presence in high concentrations may indicate improper production processes or poor quality of raw material [11].

Methanol, a toxic compound formed from the degradation of sugarcane pectin, is undesirable due to its severe health risks, including blindness and death. Bortoletto and Alcarde [23] found that methanol was responsible for the slightest disagreement with Brazilian law, with only 2.6% of evaluated samples exceeding the legal limit.

PAHs are genotoxic and carcinogenic compounds in various food products and beverages, including cachaça. Menezes et al. [26] developed a method to identify 16 PAHs in artisanal sugarcane spirits, revealing the need for strict production control to minimize PAH presence, as Brazilian legislation does not establish a limit for PAHs in cachaça [19].

Therefore, high-quality sugarcane spirits production requires strict control of raw material quality, fermentation, and distillation conditions. Minimizing undesirable secondary compounds such as acrolein, 1-butanol, 2-butanol, copper, EC, furfural, HMF, PAHs, and methanol can be achieved through careful monitoring and adherence to good manufacturing practices. Regular final product analysis is essential to ensure compliance with national and international regulations and consumers’ safety and satisfaction.

By maintaining strict control over the production process and monitoring the presence of undesirable secondary compounds, producers can minimize the impact of these compounds on the quality and safety of cachaça. Continued research and development of analytical methods, such as those by Guerreiro et al. [27] for EC determination and Menezes et al. [26] for PAH identification, can enhance the robustness and reproducibility of quantitative results, allowing for more effective monitoring and management of these contaminants in cachaça production.

Collaborative efforts between researchers, regulatory agencies, and industry stakeholders are necessary to establish appropriate limits and guidelines for undesirable secondary compounds in cachaça. By working together to develop and implement effective strategies for minimizing these compounds, it is possible to improve the overall quality and safety of cachaça, enhance consumer satisfaction, and facilitate market expansion for this unique and culturally significant beverage.

### 1.4. Word Cloud

**Figure 4:**
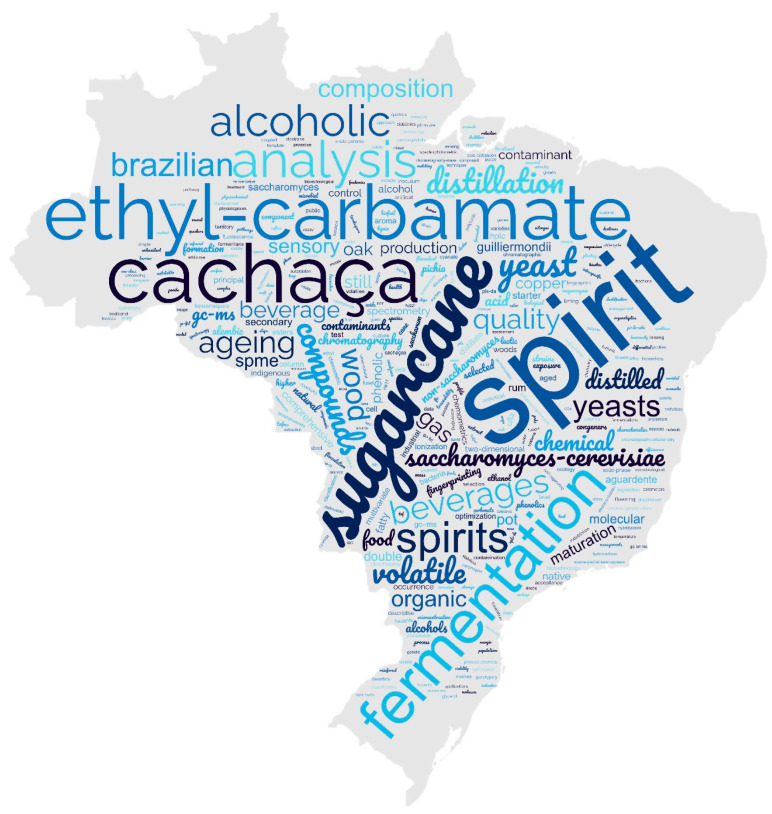
Cachaça word cloud.

A word cloud was developed based on the keywords available in the literature (Figure 4) that shows that several factors affect cachaça production, including the use of different distillation techniques such as pot stills made of copper or stainless-steel, the use of various yeasts, the presence of lower fatty acids and fusel alcohols, and the formation of EC during and after distillation. EC, a carcinogenic compound, is a significant concern in cachaça production and analysis, with research focusing on its occurrence, formation pathways, and biological activity. Analytical methodology for EC detection and its precursors is also a current trend.

Researchers have also investigated molecular diversity in fermentation and the genetic characterisation of yeasts for cachaça production. Ageing markers such as phenolic compounds and volatile organic compounds (VOCs) are being studied to improve the quality of aged beverages. Analytical techniques include comprehensive two-dimensional gas chromatography, solid phase extraction fingerprint, and volatile compounds analysis. Wood species, such as Brazilian oak, are tested for suitability in cachaça maturation.

The use of non-*Saccharomyces* yeasts, sensory analysis, and the flocculent nature of *S. cerevisiae* are also areas of interest. Furthermore, studies on the quality, chemical composition, and contaminations such as EC have shown that cachaça production requires further quality control measures.

## 2. Fermentation

The quality of cachaça mainly depends on the yeast strains used during the fermentation process. To produce high-quality sensory, chemical, and toxin-free cachaça, it is essential to have a good understanding of the yeast and bacteria populations present during the fermentation process. The yeast strain not only affects the alcohol content of the final product but also contributes to its sensory characteristics.

### 2.1. Role of Yeast in Cachaça Production

Fermentation plays a crucial role in the production of cachaça, a distilled sugarcane juice-based alcoholic beverage popular in Brazil. The yeast species are primarily responsible for fermentation, with only a few other species playing a minor role. The dominant yeast species observed during the fermentation process is *S. cerevisiae*, which increases in count from the start to the end of the fermentation cycle. At the same time, non-*Saccharomyces* strains disappear during the final stages. Guerra et al. [28] focused on the fermentation process in an open vat with possible adjustments to sugar concentration and the addition of nutrients. The authors noted that sugarcane juice alone is not a suitable growth medium; thus, maize flour was added to provide nutrients. It is essential to highlight that according to Brazilian legislation, adding flour maize or other nitrogen sources is not allowed. The study collected samples only when the fermentation progressed well according to the producer’s standards. Overall, they provide valuable insights into the fermentation process of cachaça production and the importance of nutrient supplementation.

Studies have been conducted on the yeast strains used in the production of cachaça. A total of 30 yeast strains were evaluated, with 24 being *S. cerevisiae* and six belonging to other species, such as *Candida apicola*, *C. famata*, and *C. guillermondii* [29]. These yeast strains were isolated from three small-scale cachaça distilleries in Minas Gerais, Brazil. The researchers found that yeast is the primary factor influencing the flavour and aroma of cachaça, and different yeast strains did not show significant differences in the sensory attributes of aroma and flavour [30].

Studies have also been conducted on *S. cerevisiae* yeast strains that produce cachaça. The production of cachaça dates to the Portuguese colonisation period, and 90 *S. cerevisiae* strains were isolated from cachaça distilleries in different regions of Minas Gerais, Brazil [31]. The UFMGA 905 strain was used as a reference and presented favourable fermentation and sensory characteristics. The strains were maintained on GYMP agar at 4 °C or in liquid nitrogen, and 15 strains were evaluated for specific sedimentation velocity. The research highlights the critical role yeast plays in cachaça production and its impact on the final characteristics of the beverage.

Studies have also examined the impact of yeast species on cachaça production. Morais et al. [32] isolated yeast species from sugarcane juice in the Dominican Republic and found that yeast population changes during sugarcane spirit fermentation, with *S. cerevisiae* becoming the dominant species. They also found that the pH, ethanol content, and high sugar levels due to the daily addition of sugarcane juice may play a role in selecting the dominant yeast. Another study by Gomes et al. [16] examined the ability of cachaça fermentation strains to produce high levels of sugared trehalose to make the beverage. The results indicated that *Schizosaccharomyces pombe* strains were well adapted to the conditions of cachaça fermentation and showed similar behaviour to *S. cerevisiae* strains isolated during fermentation.

A study on the genetic diversity of *S. cerevisiae* strains collected from spontaneous fermentations during alembic cachaça production in Brazil showed a high variation among the strains [28]. The yeast strains isolated during the 24 h fermentation cycle showed that *S. cerevisiae* was the dominant species in most fermentation intervals across the three distilleries studied. In addition, other yeast species, such as *Schizosaccharomyces pombe*, are well-adapted to the conditions of cachaça fermentation.

Yeast is mainly responsible for the fermentation process and results in the significant genetic variation in the yeast community in Brazil [33]. The chemical composition of cachaça was investigated using mass spectrometry [34]. The study evaluated the chemical profile of cachaças produced by 14 yeast strains that presented appropriate fermentative characteristics.

*S. cerevisiae* is a prevalent yeast strain during the cachaça fermentative process. In modern industrial processes, adding cultured *S. cerevisiae* strains is recommended to speed up the fermentative process, increase desired metabolite level and prevent the production of harmful components by microbial contaminants [14].

A new strategy has been developed for isolating yeast strains used in the production of cachaça [35]. The yeast strains described in the study were isolated from a cachaça distillery in Ouro Preto, State of Minas Gerais, Brazil. All strains demonstrated the ability to grow under similar conditions as those encountered during the fermentation of the sugarcane must. The differences in the ITS (Internal Transcribed Spacer) were used to identify yeast species, and the taxonomic identity of these strains was confirmed by cloning and sequencing the rDNA internal transcribed spacer region.

In a study aiming to select starter yeasts for cachaça production, *S. cerevisiae* strains from different regions of Minas Gerais, Brazil, were isolated and characterised. The study involved 19 trained or consuming panellists, and some results contradict prior research. For example, the authors found that some strains resistant to TFL (Trifluoroleucine) and cerulenin were analysed through chromosomal patterns, mtDNA RFLP (Restriction Fragment Length Polymorphism), RAPD-PCR with the primers EI1 and LA1, analysis of six microsatellite loci, and COX1-PCR analysis [36].

The study of indigenous *S. cerevisiae* strains in sugarcane juice found that yeast strains isolated from bioethanol distilleries and fruit wine fermentations could grow in high temperatures. They were osmotolerant and sulfite-tolerant [37]. A mixed culture of *S. cerevisiae* and *Pichia caribbica* was used to investigate the fermentation of sugarcane juice, and 20 non-*Saccharomyces* strains isolated from fruit wine fermentation, coffee fermentation, and sugarcane silage were evaluated. Duarte et al. [38] found that the ability to grow at 12% ABV is an essential factor for any strain in cachaça production and that using different yeast strains can affect the sensory qualities of the final product.

Brazil is a leading bioethanol producer, and its production is increasing rapidly. However, a study showed that in approximately 40% of the distilleries involved, no selected yeast strains could be successfully implanted to compete with the indigenous yeasts. This resulted in economic losses due to foaming, flocculation, high glycerol formation, and high residual sugar. The authors argue that future work should be performed to identify any technological advantages of these variants, mainly regarding better adaptability or higher stress tolerance during fuel ethanol fermentations [39].

The role of yeast flocculation in alcoholic beverage production, including cachaça, is an important phenomenon. A study by Soares [40] described yeast flocculation as the aggregation of yeast cells into clusters known as flocs. Flocculation is a low-cost and easy method in brewing for separating yeast cells from the broth. The FLO1 gene is responsible for flocculation and encodes a cell wall protein. The study highlights the benefits of using flocculent yeast strains in alcoholic beverage production and discusses the aggregation of yeast cells as a form of social behaviour. The presence of specific genes, such as Flo1, Flo5, Flo9, Flo10, and Flo11p, increases yeast cell wall hydrophobicity, and the study supports previous research that CSH (Cell Surface Hydrophobicity) is partially responsible for triggering the flocculation of brewing strains.

In cachaça production, fermentation parameters such as ethanol yield, productivity, and maximum specific cell growth velocity are crucial for yeast strain performance during fermentation. In total, 13 *S. cerevisiae* strains isolated from cachaça distilleries were studied to understand and explain their flocculation behaviours. Yeast flocculation is the asexual, calcium-dependent, and reversible aggregation of cells into flocs with subsequent rapid sedimentation from the medium in which they are suspended. The yeast strains were selected based on *S. cerevisiae*’s biochemical and molecular characterisations [13].

**Figure 5:**
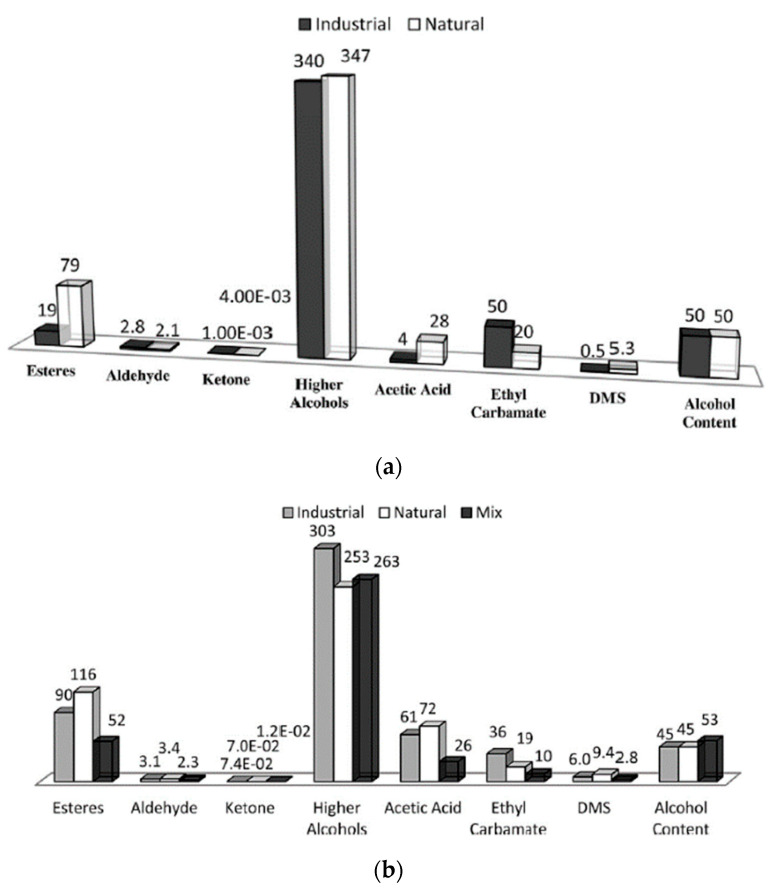
Chemical profiles (mg/L) of (**a**) 14 cachaças samples produced with natural fermentation and others 30 with industrial fermentation, distilled in stainless steel columns, and (**b**) 30 cachaças samples fermented with industrial, 19 with natural yeasts and others 12 with a mix of them, distilled in copper pot stills (alembics). Reprinted with permission from Serafim and Franco [42].

Three studies from the University of São Paulo aimed to investigate the potential role of yeast strains in cachaça production and its effect on the final product. In the first study, da Conceicao et al. [41] isolated 118 yeast strains from cachaça distilleries in several states of Brazil and performed a physiological survey. The authors found that robust industrial *S. cerevisiae* strains are ideal for high gravity bio-ethanol fermentation and concluded that yeast strains from cachaça distilleries could produce bioethanol. The second study, conducted by Serafim and Franco [42], analysed the chemical traceability of natural and industrial yeasts used to produce cachaça. The authors compared the chemical composition of 105 commercial cachaças from different producers in the State of Sao Paulo and found the concentrations of esters, aldehydes, ketones, EC, dimethyl sulfide (DMS), and acetic acid varied considerably. The study also found that cachaças distilled in copper pot stills exhibited low discriminating ability, likely due to chemical information loss in the head and tail fractions. The results suggest that the chemical profiles of cachaça are sensitive to fermentation methodologies and can be used as a tool for discrimination and pattern recognition of yeast strains used during fermentation, which could provide consumer information regarding the authenticity and traceability of the product (Figure 5). In the third study, Parente et al. [43] investigated the potential of the industrial strain *D. bruxellensis* GDB 248 in producing handmade cachaça. The authors performed fermentation experiments in laboratory media. They found that the yeast population almost doubled with the supplementation of aromatic and branched-chain amino acids, which strongly influenced the production of aromas. However, the yield of glycerol in the study was lower than that of *S. cerevisiae*, a commonly used yeast in the production of cachaça.

Figure 5a,b display the chemical profiles of 14 cachaças produced using natural fermentation and 30 with industrial fermentation, distilled in stainless-steel columns, respectively. Significant differences were observed in the concentrations of esters, aldehydes and ketones, EC, DMS, and acetic acid. Cachaças derived from natural yeasts exhibited higher total ester concentrations, dominated by ethyl lactate and ethyl acetate. Conversely, ethyl butanoate, ethyl hexanoate, ethyl octanoate, isoamyl octanoate, and ethyl decanoate contents were higher in cachaças from industrial fermentation. This figure offers valuable insights into the chemical composition of cachaças produced via different fermentation methods, aiding the understanding of factors impacting cachaça quality and informing strategies for production improvement [42].

The presence of native yeasts in the production process significantly impacts the chemical and sensory profiles of cachaça. A study by Portugal et al. [18] evaluated the performance of different yeast strains in the production of cachaça. The results showed that strains such as *Meyerozyma guilliermondii* and *Hanseniaspora guillierdii* had a more significant impact on the flavour complexity of cachaça than *Pichia fermentans*, which was found to have the lowest ethanol yield. The authors suggest that the role of native microbiota should be considered a cornerstone in the profiling and traceability of cachaça. Another study by Araújo et al. [44] isolated yeast strains from cachaça distilleries in four Brazilian states and evaluated their biotechnological properties. The authors found that strain LBCM1045 showed good fermentation performance at low temperatures and desirable production of acetate esters. However, the higher production of phenolic compounds requires further work to facilitate its utilisation in producing lager beers.

On the other hand, the genome of *S. cerevisiae*, the yeast used in rum and cachaça production, has been sequenced for the first time. Costa et al. [45] described the complete genome sequence and analysis of an *S. cerevisiae* strain used for sugarcane spirit production. The cachaça yeast strain BT0510 was sequenced using an Illumina MiSeq platform and showed a complex genetic variability. The differences among the strains highlight how strains with different genomic endowments can accomplish the same fermentative process. Whether the differences are due to genetic drift or distinct selective pressures will require further analysis.

The role of yeast in cachaça production is essential for fermentation, resulting in significant genetic variation in Brazil’s yeast community. The dominant yeast species in cachaça fermentation is *S. cerevisiae*, which increases in count from the start to the end of the fermentation cycle. At the same time, non-*Saccharomyces* strains disappear during the final stages. Yeast is the primary factor influencing the flavour and aroma of cachaça, and different yeast strains do not show significant differences in the sensory attributes of aroma and flavour. The impact of yeast species on cachaça production has also been examined, with *S. cerevisiae* being the dominant species in most fermentation intervals across the three distilleries studied. In modern industrial processes, adding cultured *S. cerevisiae* strains is recommended to speed up the fermentative process, increase desired metabolite levels, and prevent the production of harmful components by microbial contaminants. Finally, a new strategy has been developed for isolating yeast strains used in the production of cachaça, and indigenous *S. cerevisiae* strains in sugarcane juice are well-adapted to the conditions of cachaça fermentation. Table 3 shows the principal yeast and bacteria that affect the fermentation process.

### 2.2. Improvement of Cachaça Quality

Cachaça production in Brazil has increased its focus on quality and the international market due to its flavour and aroma. Several studies have been conducted to improve the quality of cachaça by optimising the yeast strains used for fermentation and adding nitrogen sources. The impact of these factors on fermentation is crucial to producing high-quality cachaça. Researchers have analysed the secondary components of cachaça, including volatile substances that distil with ethanol and water, to improve quality [53].

In recent years, studies have focused on the role of yeast strains in shaping the chemical and sensory profiles of cachaça. Indigenous yeast strains have been used to improve the quality of Cachaça. Researchers screened 3014 yeast isolates, and 916 (30%) showed a higher resistance to various types of stress, including high osmolarity, high alcohol content, and high temperature. Fifteen yeast strains were selected for further study [54]. Yeast strains used in the fermentation process were selected based on their results in the biochemical tests used for identification and characterisation.

Studies have evaluated the use of various organic nitrogen sources, such as soy protein isolate and yeast extract, to improve the fermentation process and the chemical and sensory quality of cachaça. A pilot scale study found that adding nitrogen derived from protein sources improved the fermentation time and productivity. However, adding protein sources did not affect the sensory quality of the cachaça. In another study, a mixed inoculum of *S. cerevisiae* and *M. caribbica* positively influenced the final quality of the cachaça [55]. In another study, Jeronimo et al. [56] conducted a pilot-scale batch fermentation process using soy protein isolate as an organic protein nitrogen source to supplement sugarcane juice to assess its effect on yeast viability and cachaça quality. This provides insights into the potential of soy protein isolate as a protein supplement and its impact on the chemical quality of the resulting cachaça.

The quality of cachaça also depends on the raw material used, the treatment of the sugarcane juice, and the type of yeast used. Clarifying sugarcane juice resulted in a must with better technological characteristics, with significant amounts of TA (Total Acidity) and TPC (Total Phenolic Content) removed. The use of selected yeast strains, such as *S. cerevisiae* CA-11, in the production of cachaça is recommended [57].

Several studies have been conducted to improve the quality of cachaça production by understanding the role of yeast and developing effective strategies. One study utilised a mixed culture of *S. cerevisiae* and *L. fermentum* to produce cachaça and analysed the impact of co-inoculation on the quality of the beverage. The study involved 50 untrained panellists who evaluated the composition of the head and tail fractions. The results showed that co-inoculation of yeast and bacteria positively influenced the quality of cachaça, and the head and tail fractions were identified as potential sources of compounds with economic potential for future studies [15].

In another study, researchers investigated the use of non-*Saccharomyces* yeasts in the production of cachaça. The authors found that a mixed inoculum of *S. cerevisiae* and *M. caribbica* positively influenced the final quality of the cachaça. The study concluded that the protein extracted directly from the must and the MALDI-TOF MS technique helped monitor the yeast inocula during the fermentative process [48]. Additionally, a study of yeast strains used to produce cachaça in Germany found that while some strains were unsuitable for industrial bioethanol production, there was a high degree of potential for industrial application of the strain LMQA SNR 65 [58].

Finally, a study analysed the chemical composition of cachaça produced using selected yeast CA-11, resulting in better quality distillate than natural yeast. The study found a lower coefficient of congeners, total aldehydes, total esters, and volatile acidity, indicating a higher-quality product [59]. Gonçalves et al. [60] mentioned that one barrier to increased exports is the variation in chemical composition between harvests, primarily resulting from inadequate control in the fermentation process. Utilizing selected strains of *S. cerevisiae* as starters in the fermentation process presents an alternative for achieving consistent sensory characteristics and superior quality. This study evaluated the chemical composition of cachaças produced by spontaneous fermentation and selected yeast strains. All samples displayed concentrations of volatile compounds within the recommended limits; however, cachaças produced using selected strains exhibited lower volatile acidity values and less variation than spontaneous fermentation. Overall, these studies provide valuable insights into the role of yeast in cachaça production and suggest strategies for improving the quality of the beverage.

The improvement of cachaça quality has become a focus of research in recent years due to its flavour and aroma and the increased demand for high-quality cachaça in the international market. Studies have explored the role of yeast strains in shaping the chemical and sensory profiles of cachaça, and researchers have screened various yeast isolates to find those with higher stress resistance. Indigenous yeast strains have been used to improve the quality of cachaça, and yeast strains used in the fermentation process are selected based on their results in biochemical tests. Additionally, researchers have investigated the use of various organic sources of nitrogen to improve the fermentation process and the chemical and sensory quality of cachaça.

Furthermore, the quality of cachaça depends on the raw material used, the treatment of the sugarcane juice, and the type of yeast used. Clarifying sugarcane juice resulted in a must with better technological characteristics, and the use of selected yeast strains, such as *S. cerevisiae* (i.e., CA-11), in the production of cachaça is recommended. The use of mixed cultures of yeast and bacteria has also been found to influence the quality of cachaça positively, and the head and tail fractions have been identified as potential sources of compounds with economic potential.

Overall, these studies provide valuable information about the role of yeast in cachaça production and suggest strategies for improving the quality of the beverage. Additional studies are required to investigate the capabilities of various yeast strains and fermentation processes in producing cachaça of higher quality, especially in terms of its chemical composition and sensory attributes, while ensuring the absence of harmful substances.

### 2.3. Alembic Cachaça Production

Minas Gerais is home to approximately 8000 traditional distilleries producing 230 million litres of cachaça annually. The study of the population dynamics of yeast in the fermentation process found that using flocculent starter *S. cerevisiae* strains could improve the process and reduce H_2_S production. *S. cerevisiae* was found to be the dominant yeast in the spontaneous fermentation of alembic cachaça [61]. The molecular diversity of *S. cerevisiae* strains was determined through PCR fingerprinting using an intron splice primer, mitochondrial DNA restriction analysis, and RAPD-PCR. The study showed that differentiating the indigenous *S. cerevisiae* strains was possible [62].

A genetic diversity study of *S. cerevisiae* strains associated with cachaça production found that PCR directly from a colony was a convenient and rapid protocol for amplifying target DNA. However, the only limitation of this method is that no stock is available for additional DNA studies [49]. Gomes et al. [63] studied three indigenous *S. cerevisiae* strains isolated from cachaça distilleries in Minas Gerais, Brazil, and found these strains could ferment in temperatures between 43 and 46 °C and resist between 10 and 14% ABV.

A different study by Carvalho-Netto et al. [64] identified bacterial contamination in the fermentation process of cachaça by sequencing the DNA of the yeast used in beverage production. Sedimented yeast samples were collected from a still in Monte Alegre do Sul São Paulo State, Brazil. The results showed that the samples differed in bacterial composition and that *Lactobacillus hilgardii* was shared among the three samples.

The chemical compositions of different brands of cachaça produced by alembic distilleries in Minas Gerais state, Brazil, were compared in a study by da Silva et al. [65]. The study used yeasts isolated from distilleries in Minas Gerais to produce cachaça on a laboratory scale. Nova et al. [66] studied the population dynamics of yeast in the fermentation of sugarcane in cachaça stills and found that *S. cerevisiae* dominated the yeast population at the end of the process.

De Aguino and Franco [67] investigated the formation of dextran deposits in cachaça. The data obtained from this study can be applied in developing procedures to minimise the dextran problem in cachaça. The production of alembic cachaça, the traditional use of a natural starter ferment known as “fermento caipira”, is still prevalent among small producers in Brazil. The ferment is made from a mixture of crushed corn, rice bran, and citric fruit juice added to the sugarcane juice [68]. They analysed various parameters of the sugarcane juice samples, including pH, titratable acidity, phenolic compounds, Pol, Brix, purity, reducing sugar, total reducing sugars and protein. The results showed that yeast numbers decreased as the juice reached its maximal maturity, with an increase in the proportion of *Saccharomyces*. The primary microorganisms associated with the juice were primarily from soil and plants.

Several studies have investigated the yeasts and bacteria involved in fermentation and their impact on the final product. Gomes et al. [69] found that the indigenous strain of cachaça yeast was better adapted to the fermentation conditions and had higher viability than a commercial strain of *S. cerevisiae*. Similarly, Guerra et al. [28] studied three indigenous *S. cerevisiae* strains isolated from cachaça distilleries and found that strain UFMG-A905 produced the best results for fermentation parameters compared to the other strains tested. The results showed that three strains could ferment in temperatures between 43 and 46 °C and resist between 10 and 14% ABV. In another study, de Souza et al. [70] investigated some characteristics of cachaça produced by a selected commercial wine strain. They found significant variations in galactose and maltose utilisation, resistance to sulphur dioxide, and ethanol.

*Lactobacillus* was the predominant bacterium in the samples collected from two traditional cachaça distilleries in Minas Gerais [71]. Furthermore, an uncultured bacterium clone was the most prevalent after 45 days of fermentation [72]. Duarte et al. [38] found that using a mixed inoculum of *S. cerevisiae* and *M. caribbica* improved the final quality of cachaça, but further research is necessary to understand the technique and its potential. Portugal et al. [73] found that isobutanol levels were almost twice the average reported for Brazilian cachaças, and isobutanol contributes to the fruity and sweetish sensory impressions.

Stroppa et al. [74] studied the best strains of cachaça yeasts and found that the RM01 and CV01 strains were the predominant yeasts from two cachaça distilleries in the State of Minas Gerais. Badotti et al. [75] conducted genome-wide association studies to identify the strains of *S. cerevisiae* used in traditional cachaça fermentations in Brazil and found two interbreeding populations of *S. cerevisiae* strains coexist in cachaça fermentations.

Lastly, Brexó et al. [76] reported that 38% of 134 yeast isolates obtained from malt bagasse and indigenous inoculum had biotechnological relevance, and *S. cerevisiae* CSC 23 was found to have high encapsulation efficiency and could be a good candidate for microencapsulation.

The role of yeast in cachaça production is critical as it is responsible for the fermentation process that converts the sugarcane juice into alcohol. A study found that using flocculent starter *S. cerevisiae* strains can improve the fermentation process and reduce the production of H_2_S. *S. cerevisiae* was found to be the dominant yeast in the spontaneous fermentation of alembic cachaça. The genetic diversity of *S. cerevisiae* strains was determined using different methods, such as PCR fingerprinting and mitochondrial DNA restriction analysis. The indigenous *S. cerevisiae* strains could ferment in temperatures between 43 and 46 °C and resist between 10 and 14% ABV. In contrast, bacterial contamination, particularly by *Lactobacillus hilgardii*, was found in the fermentation process. Studies have shown that the indigenous strain of cachaça yeast is better adapted to the fermentation conditions and has higher viability than commercial strains of *S. cerevisiae*. Using a mixed inoculum of *S. cerevisiae* and *M. caribbica* improved the final quality of cachaça. However, further research is necessary to understand the technique and its potential. Overall, yeast plays a crucial role in determining the quality and characteristics of cachaça, making it an essential factor in the production process.

## 3. Distillation Process

During the distillation process, several compounds are recovered and concentrated (i.e., aldehydes, ketones, carboxylic acids, alcohols, esters, DMS, higher alcohols, ethyl esters, and organic acids). In addition, different materials, such as copper, stainless-steel, etc., affect these compounds’ levels. The use of different types of stills in the production of cachaça also affects its chemical composition. Furthermore, the distillation process separates cachaça into three sequential fractions: the head, the heart, and the tail. The heart, the fraction with the lowest concentration of unwanted compounds, is the commercial interest of the distillation and is characterised by a clear and colourless aspect.

### 3.1. The Chemical Composition of Cachaça

Several studies have investigated the chemical composition of sugarcane spirits and found that they contain various compounds such as aldehydes, ketones, carboxylic acids, alcohols, esters, DMS, higher alcohols, ethyl esters, and organic acids. Distillation using different materials, including copper, stainless steel, aluminium sponge, and porcelain balls, can affect the levels of these compounds. Free acids produced during fermentation are also present but are not considered toxic. Simple and fast methods such as GC-FID have been developed to analyse these beverages’ acids and other compounds. One such method presented by Nascimento et al. [77] can be applied to spirits and other liquors, including cachaça. Another study by Nonato et al. [78] presents a method based on headspace solid-phase microextraction (SPME) and gas chromatography for identifying and quantifying higher alcohols and ethyl acetate in cachaça.

Viela et al. [79] examined the physical-chemical composition of cachaças and identified the characteristic profile of the beverage, including sufficient concentrations of higher alcohols, esters, and aldehydes. Furthermore, a method for determining fatty esters in cachaça found that those produced in copper and hybrid alembic exhibit higher ethyl acetate and ethyl lactate content. In contrast, those produced in a stainless-steel column tend to exhibit a higher content of ethyl octanoate, ethyl decanoate, and ethyl laurate [80].

Tábua et al. [81] analysed volatile compounds in Mozambican spirits and found that they contained 35 compounds, of which 19 were esters. The compounds were categorised into alcohol, ester, aromatic hydrocarbon, acetal, and aldehyde. Although glycerol was detected in trace amounts, no significant zinc, iron, cadmium, or chromium concentrations were found. In contrast, another study analysed the volatile components and contaminants of sugarcane spirits in Brazil and found that the chemical composition of the alcoholic distillates was not affected by the alcohol content of the phlegm, which ranged from 30% to 48% ABV [3].

The chemical composition of cachaça is a crucial aspect to consider when discussing the production of this traditional Brazilian spirit. Various research studies have employed techniques such as gas chromatography and solid-phase microextraction to determine the primary components of cachaça. These studies have identified water, ethanol, higher alcohols, ethyl esters, aldehydes, ketones, and organic acids as the main constituents of cachaça.

Interestingly, the use of different types of stills in the production of cachaça affects its chemical composition. While copper or aluminium pot stills have been found to result in lower DMS levels, they also tend to increase the levels of sulfate and methanol. In contrast, cachaças produced in a stainless-steel column tend to exhibit a higher ethyl octanoate, ethyl decanoate, and ethyl laurate content. Furthermore, studies have found that cachaças produced in copper and hybrid alembic exhibit higher ethyl acetate and ethyl lactate content.

Finally, it is noteworthy that the chemical composition of cachaça is not affected by the alcohol content of the phlegm, which can range from 30% to 48% ABV. Thus, the chemical composition of cachaça is influenced by several factors, including the type of still used, and identifying these components is essential for producing high-quality cachaça.

### 3.2. Controlling the Distillation Process

Distillation is a crucial step in the production of cachaça and can impact its sensory and chemical properties. The copper alembic and stainless-steel column are the two main distillation methods used in cachaça production, with the former producing a small batch of unique and differentiated cachaça while the latter produces a standardised version at a large scale [3].

Industrial cachaça is produced continuously in a stainless-steel column. The fermented must is continuously introduced at the top of the column, flowing through trays while heated by water vapour. The vaporisation of the alcohol and volatile compounds separates them from the liquid, which accumulates at the bottom of the column. The control of DMS is essential to avoid its accumulation and consequent transfer to the cachaça [3].

New standards for the identity of cachaça have reduced the copper content of sugarcane spirits [82]. Neves et al. [83] found that solid calcium and magnesium carbonates can act as cation exchangers, removing most copper ions. Reche and Franco [84] analysed 115 cachaça samples distilled in copper stills or stainless-steel columns, examining 35 items using chromatography and spectrometry. Chemometric techniques were employed to distinguish between the two cachaça groups. A classification model was developed with 91.7% accuracy in predicting the distillation apparatus used for unknown samples using specific chemical descriptors. In a study by Fernandes et al. [85], the physicochemical quality of cachaças from Minas Gerais was evaluated. Although the blended samples met acceptable standards, 43.75% of individual products exceeded quality limits. They recommended best practices during manufacturing to prevent contamination and emphasized the importance of monitoring copper levels. Training and collaboration among producers could help improve production management and ensure that cachaças meet physicochemical standards, enhancing both quantity and quality.

Santiago et al. [86] examined the physicochemical profiles of cachaça produced in copper stills over six years in Minas Gerais, Brazil. Minas Gerais is a focal point for small-scale alembic cachaça production using copper stills. They analysed samples from various cities in Minas Gerais between 2006 and 2011, discovering that 61.5% and 77.9% of samples met the identity and quality standards set by Brazilian legislation. The study’s Principal Component Analysis (PCA) suggested that alembic cachaças were homogeneous, indicating a good percentage of product acceptability across the consumer market.

In a study by Alcarde et al. [87], the authors examined the fermentation and distillation process of cachaça. The study found that the ethanol concentration in the distillate decreased linearly during distillation, with the most volatile compounds being distilled at the beginning. The study also found that the less volatile compounds, e.g., acetic acid, were distilled in the final fractions. Another study by Silva et al. [88] found that reducing copper content during double-distillation is common in other distilled beverages such as whiskey, rum, and brandy. According to the study, the tail fraction’s degree of removal directly influences the distillate’s copper content.

A study by Franitza et al. [89] isolated volatile compounds from 51 rum samples and analysed them using GC×GC-TOF-MS. The study found that the total set of volatiles in the final rum is influenced by the raw material used and the production process, including the type of yeast, distillation process, and ageing time. Additionally, three spectrophotometric methods have been developed to determine the concentration of copper in sugarcane spirit. About 75% of the samples had copper concentrations above the limit established by Brazilian legislation, and the proposed spectrophotometric methods were susceptible, with acceptable limits of detection (LODs), precision, and accuracy [90].

Industrial cachaça is commonly produced using a stainless-steel column. However, controlling DMS during production is essential to avoid its accumulation and subsequent transfer to the cachaça. New standards for the identity of cachaça have reduced the copper content of sugarcane spirits. The reduction in copper content during double-distillation is typical in other distilled beverages.

The distillation process separates the alembic cachaça into three sequential fractions: the head, the heart, and the tail, each containing different volatile compounds. Studies have found that the ethanol concentration in the distillate decreases linearly during distillation, with the most volatile compounds being distilled at the beginning. The less volatile compounds, e.g., acetic acid, are also distilled in the final fractions.

The type of yeast, distillation process, and ageing time all influence the final set of volatiles in cachaça. About 75% of the samples had Cu concentrations above the limit established by Brazilian legislation, and the proposed spectrophotometric methods were reliable.

### 3.3. Monitoring and Improving Cachaça Production Processes

Cachaça production is a complex process that can introduce potential contaminants that affect the quality of the beverage, such as DMS. In order to produce high-quality cachaça, it is essential to control and optimize the distillation process. Cardoso et al. [91,92] developed a new method for detecting DMS in alcoholic beverages, indicating the need for improved production processes to enhance the flavour quality of cachaça. Reche et al. [93] analysed the database of Brazilian cachaças for 34 constituents, including copper, ethyl acetate, and phenyl methanal, while Cardeal et al. [94] studied aroma compounds in cachaça samples to understand the ageing process using the technique of fingerprinting for extensive sample characterization.

De Souza et al. [95] compared the volatile compounds in cachaça obtained from single and double distillation processes, finding a different composition in the latter. The effects of ion exchange resin and charcoal filters on the volatile composition of cachaça samples were also studied. A total of 55 compounds were tentatively identified using HS-SPME, including esters, aldehydes, alcohols, and ketones. Carlos et al. [96] found that 33 brands of Brazilian sugarcane spirits were non-compliant with the country’s anti-pollution laws, showing high levels of copper, cyanide, and carbonyl compounds. Negri et al. [97] analysed 152 samples of alcoholic beverages and found high levels of methanol, copper, and cyanide in suspected unrecorded alcohols in the Brazilian market.

In a study by Zacaroni [98], response surface optimization was employed to analyse the extraction conditions of volatile compounds in Brazilian sugarcane spirits using HS-SPME-GC-MS. The research focused on 14 VOCs identified through the extraction process from distilled sugarcane spirits. It employed a combination of experimental design and response surface analysis allowed for a reduction in analysis time while determining the influence of various parameters on the extraction process. Of these parameters, extraction time had the most significant positive impact on the extraction, while desorption time exhibited a negative effect, holding the slightest influence on the process. The results underscore the importance of chemometric evaluation as an efficient technique for optimizing extraction parameters, as it assesses influential parameters simultaneously, thereby reducing analysis time.

Duarte et al. [99] explored the physicochemical and sensory alterations in aged sugarcane spirits subjected to activated carbon filtering. The study aimed to evaluate the differences in composition and sensory quality pre- and post-filtering. The findings indicated that activated carbon filtering effectively lowered copper and furfural levels, adhering to national regulations. However, the filtering also diminished sensory attributes, such as yellow colour, aroma, and woody flavour. Although activated carbon filtering successfully removed specific components, it impacted the unique sensory traits of the aged sugarcane spirits.

In another study, Duarte et al. [86] assessed the physicochemical and chromatographic profiles of 11 organic sugarcane spirit samples from various Brazilian states. They discovered that 36.40% of the samples did not meet the MAPA-recommended standards for ester content, copper content, and dry extract. To ensure cachaça’s quality and authenticity, monitoring and enhancing the production process is crucial. Ferreira et al. [100] found that GC × GC two-dimensional chromatograms were highly useful in authentication problems, and chemometrics classifiers, such as DDSIMCA, could be successfully applied to chromatographic data. Additionally, do Nascimento et al. [101] developed a new method for determining higher alcohols, n-butanol, and ethyl acetate in cachaça using GC-BID by headspace solid-phase microextraction, showing concern for product quality.

Oliveira et al. [102] examined the chemical composition of cachaça produced in Paraíba, Brazil; they assessed conformity with legal parameters and identified volatile compounds in the beverages. Of 20 unaged cachaça samples, 17 were irregular, concerning at least one legislation-established parameter. The analysis identified 57 compounds, primarily consisting of esters and alcohols. While ethyl acetate has often been cited as the principal ester in cachaça, this study found higher percentages of ethyl decanoate and ethyl dodecanoate.

Potential contaminants, such as DMS, copper, and cyanide, can affect the flavour and pose health risks to consumers. Therefore, optimising the distillation process and using techniques such as ion exchange resin and charcoal filters to remove impurities is necessary. Analysing the volatile compounds and physicochemical profiles of cachaça samples can also provide valuable information on the quality and ageing process of the beverage. Moreover, novel methods, such as GC × GC two-dimensional chromatograms and GC-BID by headspace solid-phase microextraction, can detect and quantify specific compounds of interest. The findings of these studies demonstrate the need for ongoing research and development in cachaça production to ensure the beverage’s quality, safety, and authenticity.

### 3.4. The Safety and Quality of Cachaça

As the demand for cachaça increases, it is essential to ensure its safety and quality. Bortoletto et al. [103] conducted a study on the safety and quality of cachaça, focusing on all possible quality and safety hazards based on HACCP. The study found that it is possible to avoid the formation of hazardous substances by applying for GMP, Good Manufacturing Practices, and a well-designed HACCP (Hazard Analysis and Critical Control Points) program. The authors identified approximately 1000 species of weeds in sugarcane agroecosystems and the use of natural enemies such as Cotesia flavipes (parasitoid wasp) for the biological control of insect pests. These findings highlight the importance of using sustainable practices in cachaça production to ensure the safety and quality of the final product.

A study by Santiago et al. [104] investigated cachaça production focusing on standardization and quality. As a once predominantly low-income beverage, cachaça has been increasingly embraced by higher-income consumers. Producers’ investments in quality control and standardization have elevated the drink to prominence in national and international markets. The state of Minas Gerais is a leader in alembic cachaça production, generating 250 million litres annually. Among the 512 samples analysed, only 8% showed noncompliance with the maximum copper limit of 5 g/L as mandated by the MAPA. With 70% of the samples meeting MAPA’s quality standards, it is clear that these investments have positively impacted the cachaça market.

Copper contamination in cachaça production, primarily due to copper stills, challenges meeting international export regulations and ensuring product quality. Lima et al. [105] explored the use of activated carbon to remove copper while preserving important flavour components. Although they found that using at least 12 g/L of activated carbon and a shaking time of 60 min effectively reduced copper content, it also impacted other beverage components, suggesting the need for further optimization.

Duarte et al. [106] investigated kaolinite clays for copper removal, emphasizing their potential to maintain beverage quality. They found that clay samples K01, K03, and K04 were particularly effective in reducing copper concentrations below exportation limits. Meanwhile, Zacaroni et al. [107] evaluated the use of clay and activated charcoal matrices for copper adsorption, identifying the Freundlich and pseudo-second-order models best describing the adsorption process and kinetics.

Lastly, Barbosa et al. [108] explored the application of coconut fibre as a copper adsorbent in cachaça, demonstrating its efficacy in reducing copper levels and its potential as a low-cost and selective adsorbent. The combined findings of these studies highlight various promising methods for copper removal in cachaça production, emphasising preserving the beverage’s chemical and sensory properties. Further research is needed to optimize these processes and ensure compliance with established quality standards in the growing cachaça market.

However, ensuring the safety and quality of cachaça remains a challenge. Lima et al. [20] found that 50.7% of sugarcane spirits in Brazil did not meet the standards of identity and quality, with contaminants, higher alcohols, and volatile acidity being the components that showed the most significant irregularities. This study underscores the need for ongoing monitoring and improvement of cachaça production processes to ensure quality and safety standards compliance.

Cachaça production requires the application of sustainable practices, GMP, and a well-designed HACCP program to ensure the safety and quality of the final product. Monitoring and improving production processes are also crucial to meet quality and safety standards. The findings of these studies provide valuable insights for producers and regulators in the cachaça industry.

## 4. Deciphering Cachaça: Insights into Its Composition and Ageing Markers

Producing high-quality aged cachaça involves various factors, including oak barrels and ageing time. Studies have shown that ageing cachaça in oak barrels can significantly enhance its quality and chemical composition. The optimal ageing time varies, and the wood used, barrel size and preparation all play a role in the process. Researchers have explored the use of Brazilian wood casks to achieve a unique flavour profile, with different cask types significantly influencing phenolic compounds, absorbance values, and sensory scores for aroma, flavour, and colour. This section explores oak barrels’ crucial role in cachaça production, focusing on the ageing process and its impact on the spirit’s sensory properties.

### 4.1. Desirable Secondary Compounds in Cachaça Production: Organic Acids, Volatile Compounds, and Aging Effects

The production of cachaça relies on the intricate interplay of desirable secondary compounds such as organic acids, volatile compounds, and ageing effects to achieve its unique flavour, aroma, and overall quality.

Organic acids contribute to cachaça’s acidity, with volatile acidity, expressed as acetic acid, a critical quality control parameter [11]. Common organic acids formed during fermentation include acetic, lactic, formic, butyric, and propionic acids [100,109]. Acidity levels are influenced by yeast strain, fermentation duration, and distillation process temperature and duration [11].

Esters, the majority of aromatic compounds in alcoholic beverages, contribute to a pleasant, fruity aroma. Ethyl acetate and ethyl lactate are primary esters in distilled alcoholic beverages. They are crucial for balancing aroma and flavour in high-quality cachaça [110].

Aldehydes, volatile carbonyl compounds formed chiefly during fermentation, play an integral role in defining the traditional flavour and aroma of cachaça, an iconic Brazilian spirit [11]. Acetaldehyde is the most abundant among aldehydes, generated mainly during fermentation. Particularly in aged cachaça, careful regulation of aldehyde levels is essential [111,112].

Another critical component shaping cachaça’s flavour and aroma is higher alcohols, typically generated during fermentation [78,113]. Fermentation conditions, yeast cell count, temperature, and the final alcohol content can significantly influence their formation.

Acetals contribute to cachaça’s aroma by neutralizing the pungent odour of primary aldehydes or imparting refreshing, fruity, and green characteristics. The equilibrium of acetals in sugarcane spirits is sensitive to factors such as pH, ethanol content, and the type of wood utilized for barrel ageing [114].

The traditional flavour and aroma profile of cachaça, a rich mosaic of hundreds of flavour compounds suspended in an ethanol-water matrix, is subject to considerable variation due to various factors, including fermentation conditions and the ageing process. This flavour composition includes higher aliphatic and aromatic alcohols, ethyl esters, aldehydes, and terpene flavour compounds responsible for the spirit’s primary or varietal aroma [115].

Acetals, formed during ageing, contribute to cachaça’s aroma by providing fruity and floral undertones. Understanding that cachaça’s specific flavour and aroma profile can show substantial variation, contingent on different producers’ production methods and ageing processes, is crucial. Moreover, factors such as ageing, the type of wood used, and other production variables play a crucial role in defining the flavour and aroma attributes [115].

The conventional flavour and aroma of cachaça encompass a spectrum of notes, including fruity, floral, herbal, and grassy, sometimes accented with hints of caramel or vanilla [116].

Acetals, generated during the fermentation and ageing processes, imbue the spirit with fruity and floral notes [116].

The distinctive flavour and aroma of cachaça can be influenced by the distillation method and the specific types of casks used for ageing. Ageing in oak or native Brazilian wood casks can instil a slightly spicy or woody undertone to cachaça. Typically, cachaça carries fruity and floral notes and hints of sugarcane, banana, citrus, and tropical fruits [117].

Finally, acetals, originating from the distillation process, can significantly shape the aroma profile of cachaça due to their low flavour thresholds. Thus, even trace amounts of acetals can trigger noticeable changes in the overall aroma [117].

### 4.2. Ageing Cachaça: The Crucial Role of Oak Barrels

**Figure 6:**
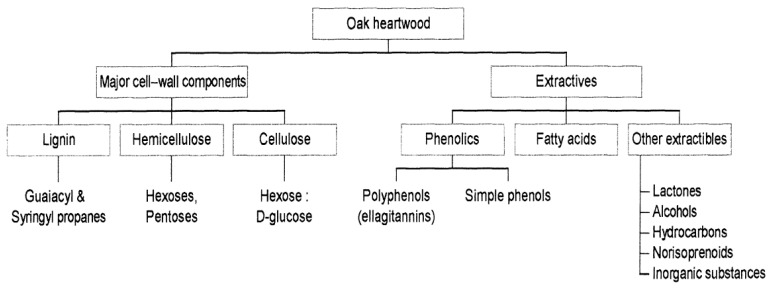
Components of oak heartwood. Reprinted with permission from Mosedale and Puech [118].

Oak barrels play a crucial role in producing high-quality aged cachaça, as studies have shown that ageing cachaça in oak barrels can significantly improve the spirit’s quality and chemical composition. The optimal ageing time of spirits varies depending on various factors, such as the raw distillate’s properties, the barrel’s size and preparation, and the wood used (Figure 6) [118].

Figure 6 outlines the components of oak heartwood. Comprising various extractive and structural compounds, oak heartwood contains ellagitannins as the most abundant extractives in unheated wood, although they are absent in spirits matured in oak casks. Oak heartwood also includes lignin, hemicelluloses, and volatile flavour components such as whisky lactone. The degradation of wood macromolecules, such as hemicelluloses and lignin, generates products, e.g., hexose and pentose monosaccharides, furan derivatives, and lignin degradation products, including aromatic aldehydes and phenols. Oak heartwood’s volatile flavour components, exemplified by whisky lactone, contribute to the distinct flavour and aroma of spirits aged in oak casks [118].

Parazzi et al. [119] evaluated the main chemical compounds of sugarcane spirit aged in oak barrels and found a significant difference between brandies stored in oak barrels and those stored in glass containers. De Auino et al. [120] developed a method for quantifying phenolic compounds found in sugarcane spirits that can be used to assess their ageing properties. Alcarde et al. [121] and Alcarde et al. [122] investigated the chemical composition of cachaça aged for over 30 years in oak barrels and found that the ethanol concentration decreased while the concentrations of phenolic acids and aldehydes increased. A positive correlation was also found between vanillin, vanillic acid, and syringaldehyde. Santiago et al. [123] aimed to evaluate the physicochemical quality of cachaça and quantify phenolic compounds and acrolein in samples stored in barrels made from different woods. The physicochemical analyses included alcohol content, volatile acidity, esters, aldehydes, higher alcohols, furfural, methanol, and copper levels. High-Performance Liquid Chromatography (HPLC/UV) determined phenolic compounds and acrolein. Phenolic compound concentrations ranged from 0.13 to 14.51 mg/L, with different predominant compounds depending on the wood type used. Acrolein concentrations in the samples were mainly below the legal limit, with one exception. Of the analysed samples, 66.6% were deemed suitable for commercialization based on physicochemical properties.

Ageing in wooden casks enhances cachaça’s overall qualities, with various wood species imparting different sensorial properties [23,124]. The ageing process increases dry extract content due to the migration of non-volatile compounds from the wood [11]. Ageing effects are influenced by wood type, charring, and previous cask usage. Lignin transformations during ageing significantly affect aged sugarcane spirits’ quality [125]. Monitoring total phenolic compound levels can ensure proper ageing and prevent fraud [124].

**Figure 7:**
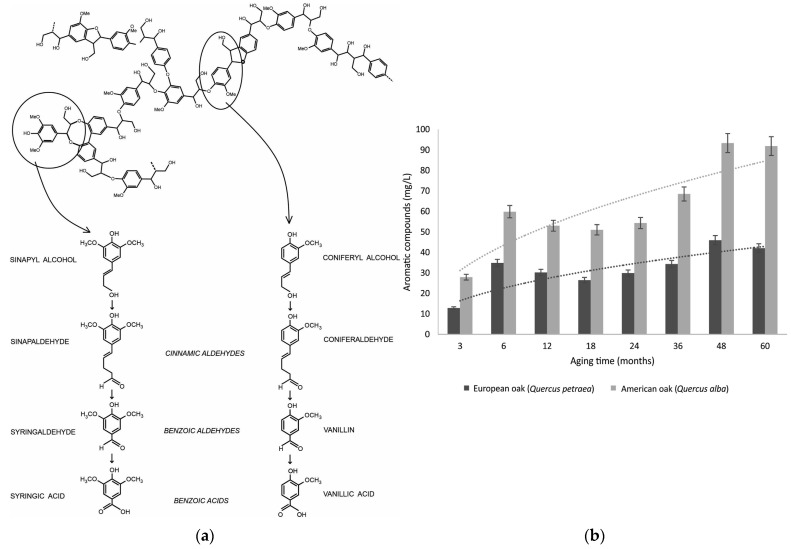
(**a**) Transformations of lignin-derived aromatic compounds during the ageing process of distilled spirits; and (**b**) the evolution of total contents of the analysed phenolic aldehydes and acids during the 60-month ageing process of cachaça in new barrels made from two oak species, European (*Quercus petraea*) and American (*Quercus alba*). Error bars represent the standard deviation of independent triplicates. Reprinted with permission from Castro et al. [129].

Ageing cachaça in new oak barrels for up to 60 months can improve the quality of the spirits [125]. They discuss the importance of ageing cachaça in wooden barrels to improve its quality, focusing on analysing the mechanism of lignin degradation during maturation (Figure 7a). The study characterises the ageing process of cachaça by analysing the contents of ageing-marker phenolic compounds extracted from the lignin of new oak barrels made from two species, European and American oak, for up to 60 months. The article identifies benzoic aldehydes and benzoic acids as the primary low molecular weight compounds derived from lignin degradation in barrel-aged cachaça, with American oak barrels conferring higher contents of these compounds than European oak barrels (Figure 7b). The study suggests that the total contents of benzoic acids can be considered for predicting the level of maturation of cachaça aged in both oak species. The results contribute to the knowledge of the phenolic composition of cachaça aged in new oak barrels, and the study highlights the importance of the wood species used for making barrels in the ageing process.

Figure 7a illustrates that during the ageing process of distilled spirits, lignin-derived aromatic compounds undergo transformations that significantly impact the final product’s quality. Low molecular weight phenolic compounds from wood are incorporated into distillates during ageing. Lignin macromolecules have branches of coniferyl (guaiacol compounds) and sinapyl (syringol compounds) alcohols. Coniferyl alcohol produces coniferaldehyde, which converts to vanillin and then oxidises to vanillic acid. Sinapyl alcohol generates sinapaldehyde, which transforms into syringaldehyde and later oxidizes to syringic acid. Lignin-derived phenolic compounds can characterise distilled spirits’ ageing level. In wine distillates aged up to 50 years, total guaiacol-type and syringol-type compound contents increased during maturation, with higher syringol-type compound contents. Likewise, grape mark distillates aged 5–6 years in European and American oak barrels had higher syringol-type compound contents than guaiacol-type compounds. In cachaça, both oak species imparted all studied phenolic compounds. The highest contents of identified phenolic compounds (cinnamic aldehydes, benzoic aldehydes, and benzoic acids) were found in American oak-aged cachaça compared to European oak-aged cachaça. Syringaldehyde and benzoic acids were the primary low molecular weight compounds in barrel-aged cachaça, regardless of oak species. Total aromatic compound contents derived from lignin degradation in cachaça aged in American oak barrels were approximately double those in European oak-aged cachaça. Overall, total low molecular weight lignin-derived compound contents stabilized or slightly decreased after 48 months of ageing in both oak species. Further investigations of high molecular weight lignin-derived compound presence in barrel-aged cachaça are recommended [125].

Figure 7b displays the 60-month evolution of analysed phenolic aldehydes and acids in cachaça aged in new European (*Quercus petraea*) and American (*Quercus alba*) oak barrels. The graph indicates the changes in analysed phenolic aldehydes and acids over time, with error bars representing the standard deviation of independent triplicates. The results reveal that the total contents of analysed phenolic aldehydes and acids increased over time in both barrel types, with a more pronounced increase in American oak barrels. Syringaldehyde and benzoic acids remained the main low molecular weight compounds in barrel-aged cachaça, irrespective of the oak species. Overall, the graph demonstrates that cachaça’s oak barrel ageing process leads to increased total contents of analysed phenolic aldehydes and acids, with a more pronounced increase in American oak barrels [125].

Silvello et al. [126] reported that ageing in new oak barrels improved the chemical and sensory profile of cachaça, while the contents of lignin degradation products found in cachaça aged in second-use extensively used barrels were similar. The researchers concluded that the composition of maturation-related congeners in the oldest samples of cachaça was in line with the average profile of phenolic compounds found in whisky aged for 12 years and cognac aged for ten years.

Castro et al. [127] discovered that the species of oak is a decisive factor in the extraction of phenolic compounds, with American oak (*Quercus alba*) barrels infusing higher contents than their French oak (*Quercus petraea*) counterparts. This suggests the superior potential of American oak barrels in the ageing process of cachaça and the consequent extraction of phenolic compounds. In addition, the study unveiled a substantial correlation between the number of barrel reuses and the phenolic compound content in aged cachaça [127]. The extraction of phenolic compounds ascended rapidly during the initial years of ageing. However, the extraction rate declined with each reuse of the barrels, despite the length of the ageing period. Notably, the rate of phenolic compound extraction in new barrels corresponds to approximately five years of extraction in extensively used barrels.

In summary, the choice of oak barrels, ageing time, and other factors play a critical role in producing high-quality cachaça, and research has shown that using oak barrels significantly improves the spirit’s quality and chemical composition.

### 4.3. The Effect of Wood Casks on Cachaça’s Sensory Properties

Wooden casks play a critical role in shaping the sensory properties of cachaça. Researchers have investigated the possibility of using Brazilian wood casks instead of oak casks to achieve a flavour profile, given the diverse flora of Brazil. Faria et al. [128] analysed the effects of ageing cachaça in casks made of eight different Brazilian kinds of wood. They found that the type of cask used significantly influenced the total phenolic compounds content, absorbance values, and average sensory scores for aroma, flavour, and colour.

**Figure 8:**
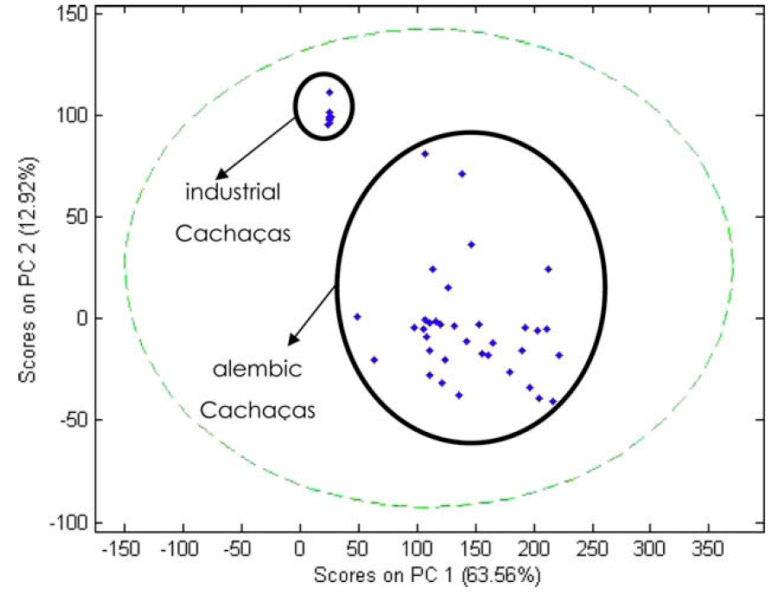
PCA score plot (PC1 × PC2) for the ESI–MS data of the pot stills and industrial cachaças. Reprinted with permission from De Souza et al. [134].

De Souza et al. [129] used high-performance liquid chromatography to determine the presence of various phenolic compounds at different concentrations in several samples of cider brandy aged in American and French oak casks. They found that direct infusion ESI–MS fingerprinting, along with exploratory statistical methods such as PCA and HCA (Hierarchical Cluster Analysis), can provide a simple, rapid, and accurate technique for analysis. De Souza et al. [130] used direct infusion electrospray ionization mass spectrometry in the negative mode (ESI–MS) to distinguish between two types of cachaça produced using different distillation apparatus, copper pot stills and industrial stainless-steel columns. The resulting spirits have subtle but characteristic chemical compositions and sensorial properties. The authors demonstrate that ESI–MS can provide fast and accurate discrimination between these two types of spirits. Additionally, they discuss the sue of PCA to analyse the ESI–MS data. It effectively distinguished the two types of cachaças, forming two distinct groups (Figure 8). The authors note that industrial cachaças use much more uniform and controlled experimental conditions than those used for pot stills cachaças, which likely explains why the industrial samples form a more uniform group.

Figure 8 presents a PCA score plot for the ESI–MS data of alembic and industrial cachaças, displaying the separation of these samples into two distinct groups. PC1 and PC2 account for 63.56% and 12.92% of the total variance, respectively, resulting in a combined total variance of 76.48% at a 95% confidence level, as denoted by the dotted line in the plot. The PCA methodology effectively evaluates the data and differentiates between the cachaça types [130].

Silva et al. [131] analysed sugarcane spirit extracts of different Brazilian woods and oak for 14 phenolic compounds and two coumarins. Their study aimed to fill the gap in the profile of phenolic compounds in aged spirits in barrels of Brazilian wood species by reporting the quantitative analysis of 14 phenolic compounds and two coumarins in sugarcane spirit extracts from 15 native wood species from Brazil.

In a study by Rota et al. [132], a panel of trained assessors evaluated the sensory profile of six samples of cachaça. The study found that the double-distillation process changed the sensory quality of the samples after ageing, and consumer preference was highest for the double-distilled sample. Santiago et al. [116] studied the physical and chemical quality of cachaça aged in casks made of different types of woods, including balsam, oak, jatoba, and peroba. The results showed that the physicochemical profiles of cachaça aged in each type of wood were different. The ethanol, aldehydes, and butan-1-ol concentrations were inadequate and outside the limits in the head and tail fractions of cachaça aged in casks.

Bortoletto et al. [133] assessed the effect of ageing on the quality of cachaça using oak chips from French forests. They found that more intensive toasting increased the content of ageing markers in cachaça, particularly syringic acid. The different origins of oak wood did not cause significant effects when compared.

Carvalho et al. [124] aged cachaça at 48% ABV in a new 20 L amburana barrel in the laboratory and found that the results were unsatisfactory compared to the predictive model fitted with fluorescence spectroscopy data. Four new maturation classes have been identified for distillates aged in wooden barrels related to extracting and transforming wood-derived phenolic compounds.

Finally, Caetano et al. [134] aimed to evaluate the sensory characteristics of non-aged alembic cachaças and develop a sensory lexicon to explain the quality attributes. Using principal components analysis and Kohonen’s neural network, the study analyzed 24 non-aged alembic cachaça samples from Salinas, Brazil. The results showed that woody, citric, and sour attributes received lower values, while sugary, fruity, sugarcane, and “rapadura” received higher values. The study suggests that using artificial neural networks is a potential approach for further studies in the field.

Overall, these studies show that the type of wood cask used and the ageing process significantly affect the sensory properties of cachaça. Further research using different analytical techniques and sensory evaluations is needed to fully understand the complex interplay between wood casks and cachaça’s sensory properties.

### 4.4. Key Secondary Compounds in Cachaça: Volatiles, Organic Acids, and Aging Impact

Phenolic compounds play an essential role in the ageing process of cachaça. Several studies have evaluated the quality and phenolic content of aged cachaças. Miranda et al. [109] analysed sugarcane spirit quality aged in 20 L oak casks for 390 days. They found that oak, commonly used for ageing cachaça by cooperage industries, had a lower variation in phenolic content than Brazilian woods. Cardoso et al. [135] compared sugarcane spirits extracted from different Brazilian woods and oak samples and assessed their antioxidant and radical-reducing capacity and their reactivity to heat. The study found that Brazilian woods have more variation in phenolic content than oak. However, the researchers noted that further studies should be conducted to identify the compounds responsible for the observed pro-oxidative effect.

Bortoletto and Alcarde [114] studied the profile of volatile compounds and specific ageing markers in sugarcane spirits aged 36 months in casks made of ten different types of wood. The study found that the sum of volatile congeners in sugarcane spirits increased over time during the ageing process, and the different types of wood influenced the profile in aged cachaças. Bortoletto and Alcarde [23] assessed the effect of ageing on the quality of cachaça using oak chips from French forests and found that more intensive toasting increased the content of ageing markers in cachaça.

Bernardes et al. [136] proposed a new method for detecting the phenolic content of aged cachaça in wooden casks to improve the drink’s quality. The optimized FC method quantifies samples with a total phenolic content between 1 and 80 mg GAE (Gallic Acid Equivalent)/L, and the authors claim that the model is of real broad applicability with successful results. The authenticity of aged cachaça was also studied using chemometric methods to discriminate commercial aged cachaças. The best results were obtained for the model based on low-level data fusion, providing reasonable reliability rates for the training and test sets for all the analysed classes [137].

Hinojosa-Nogueira et al. [138] used spectrophotometric techniques to investigate the effect of ageing time on the quality of rums in Spain. The study found that longer ageing times lead to higher concentrations of furanic compounds and higher antioxidant capacity. The authors used 42 commercial rums from 12 different countries, a sample of cachaça, and homemade rum in their analysis. The main phenolic compounds were vanillin, syringic acid, and epigallocatechin gallate. Those rums aged for longer had a more significant concentration of polyphenols.

De Castro et al. [139] assessed the phenolic composition of cachaça aged in oak barrels and glass bottles. The results showed that concentrations of furfural were higher in the samples of cachaça aged in oak barrels. The study suggests that ageing in new oak barrels improves the sensory features of distilled beverages and is the most widely used wood for ageing.

Campos et al. [140] aimed to improve the quality of aged cachaças by evaluating the concentrations of vanillic and syringic acids and other compounds found in cachaças aged in imburana (*A. cearensis*) and balm (*M. peruiferum*) woods and oak wood. The study found that thermal treatment changed phenolics’ composition, and coniferyl aldehyde and sinapaldehyde were observed in cachaças aged in jequitiba, jatoba, ipe, imburana, and balm.

These studies highlight the importance of phenolic compounds in the ageing process of cachaça and suggest that wood type significantly impacts the phenolic content and quality of aged cachaças. While oak wood is commonly used for ageing cachaça, Brazilian woods were found to have a more considerable variation in phenolic content. The studies also suggest that the concentrations of phenolic compounds can be improved by selecting the appropriate wood type, using more intensive toasting, and conducting thermal treatments. Using spectrophotometric techniques to investigate the effect of ageing time on the quality of rums provides further insight into the ageing process of distilled beverages. The proposed methods for detecting phenolic content and the authenticity of aged cachaça can help improve the drink’s quality and identify its origin.

### 4.5. Distinguishing Cachaça from Rum: Pattern Recognition Methods

Despite having distinct sensory characteristics, cachaça, a traditional Brazilian spirit, is often misclassified as rum. Both cachaça and rum are sugarcane-derived distilled spirits that have garnered international attention due to their unique sensory profiles. Although they share some production similarities, their sensory differences are significant, making sensory analysis a vital tool for evaluating their quality and establishing quality standards. Magnani [141] aimed to employ quantitative descriptive analysis (QDA) to compare the sensory profiles of rum and cachaça and identify their distinguishing attributes. A panel of seven trained judges evaluated four samples—molasses spirit, non-aged cachaça, aged cachaça, and aged rum—using QDA. The judges developed descriptors and intensity scales to characterize each sample’s sensory quality. The results highlighted notable differences in sensory characteristics, with attributes such as golden colour, body appearance, turbidity, wood aroma, wood flavour, sweet taste, and viscosity differentiating aged cachaça from rum.

Conversely, molasses spirit and non-aged cachaça were distinguished by attributes such as metallic aroma, taste, and pungent chemesthetic. Ageing was found to enhance favourable sensory attributes in aged cachaça. Magnani [141] emphasizes establishing quality standards for cachaça in the competitive international market. The findings can be utilized to develop sensory evaluation methods for rum and cachaça and establish quality standards for these spirits. Furthermore, the study provides valuable insights into the sensory characteristics of these spirits, guiding product development and marketing strategies. Overall, this research advances the understanding of rum and cachaça’s sensory characteristics and forms the basis for future studies in this field.

De Souza et al. [7] and de Souza et al. [129] described the Brazilian cachaça and suggested using direct infusion ESI–MS to differentiate between the two major types of Brazilian cachaças. In addition, Cardoso et al. [9] conducted a study comparing 28 samples of cachaça from small producers and export-grade cachaças aged in oak and balm barrels. The study found that propanol, isobutanol, and isopentanol were found to differentiate cachaças from rum.

To further differentiate cachaça, da Silva [142] developed a methodology for identifying different wood species used to mature Brazilian cachaça trees and their products. The study analysed UV-Vis absorption spectra of cachaça wood extracts and commercial samples and found that their findings could be extended to other spirits and a wider variety of wood species. Additionally, Catão et al. [143] evaluated the quality of five forest species for ageing cachaça, including Amburana cearencis. According to Brazilian legislation, the cachaças were stored in wooden barrels and glass containers for six months and then compared to the typical values.

Further studies have explored different factors that affect the flavour and quality of cachaça. Peptidic sensors were used in a study to predict the identity of unknown cachaça extracts and discriminate Cabernet Sauvignon wines based on grape maturation time [144]. Simioni et al. [145] evaluated the acceptability of cachaça stored in different woods among Brazilian and Slovakian consumers. The study found that while Brazilians preferred cachaça stored with oak, Slovakians preferred those stored with different woods.

In recent years, electronic nose systems have been developed to analyse the flavour compounds in aged sugarcane spirits [146]. The study found that the performance of the electronic nose in distinguishing between two samples of cachaça was more associated with incubation temperature and ethanol content than with stirring and equilibrium time. Further electronic nose analysis of aged sugarcane spirits will be conducted based on diluting the spirits to 10% ABV. Finally, Barbosa et al. [147] conducted a study collecting 50 L of cachaça and ageing it in four oak barrels. The study found that concentrations of furfural were higher in the cachaça aged in oak barrels compared to samples stored in a glass bottle. All samples, including the one stored in a glass bottle, contained PAHs. Pattern recognition methods such as Gas Chromatography-Mass Spectrometry (GC-MS) and principal component analysis can be used to differentiate between cachaça and rum.

The distinction between cachaça and rum is an essential aspect of cachaça production. Many studies have been conducted to develop pattern recognition methods to accurately differentiate between the two spirits. These methods include direct infusion ESI–MS, peptidic sensors, and electronic nose systems. These studies have also explored various factors that affect the flavour and quality of cachaça, such as the type of wood used to age the spirit, the incubation temperature, and ethanol content. Furthermore, studies have evaluated the acceptability of cachaça stored in different woods among consumers, with varying preferences depending on the cultural background. It is worth noting that while cachaça is often associated with Brazil, these studies can potentially contribute to the production of other sugarcane spirits worldwide.

### 4.6. The Role of Analytical Techniques in Cachaça Production

Producing high-quality cachaça requires using advanced analytical techniques to understand its chemical and sensory properties. A new approach in cachaça production has been proposed, using microfiltration and pervaporation techniques. The concentration of the volatiles in the aromatic profile was confirmed, and these compounds’ origins are still debated [148]. In another study, energy-dispersive X-ray spectrometry measured the metals in sugarcane spirit. The results indicated that the recommended conditions for extraction were pH 6.0, a sample volume of 25 mL, and a nanoparticle mass of 100 mg [149].

Analytical techniques play a vital role in understanding the chemical and sensory properties of cachaça. One such technique is Kohonen’s neural network, which establishes a chemical profile for cachaças produced in a specific region. A study by Caetano et al. [110] used principal component analysis and Kohonen’s neural network to analyse the chemical profile of non-aged alembic cachaça from the Salinas region in Brazil. The results showed that esters were the most abundant chemical class, followed by alcohols, acids, aldehydes, ketones, phenols, and copper. The study suggests that Kohonen’s neural network may be a more promising approach than principal component analysis due to its ease of interpretation and visualization.

Advanced analytical techniques, such as microfiltration, pervaporation, energy-dispersive X-ray spectrometry, and Kohonen’s neural network, are crucial in cachaça production. These techniques can provide valuable insights into the chemical and sensory properties of cachaça, leading to improved quality and flavour.

### 4.7. Analysing the Aroma Profile of Cachaça: Implications for Production and Exportation

The composition of alcohols and esters in cachaça was found to have a similar methanol content to rum but lower than wine spirit. The higher alcohols, esters, carboxylic acids, and carbonyl compounds are believed to contribute to its distinctive taste and aroma [150]. Yeast plays a crucial role in the fermentation of cachaça as it contributes to creating aromatic compounds such as “whisky lactone” [151].

Studies have been conducted to analyse the aroma of cachaça and compare it to rum. The results showed that damascenone, ethyl butyrate, methyl isobutyrate, and 2-methyl-butyrate were present at the same potency in cachaça and rum, the spicy-smelling eugenol, 4-ethyl guaiacol, and 2,4-nonadienal were much more potent in cachaça. The study showed that specific odorants were more potent in cachaça than in rum, suggesting that efforts to increase the exportation of cachaças could be aided by a better understanding of their chemical and sensory properties [10]. The aroma of rum has also been analysed, and it was found that propyl-2-methoxyphenol, γ-nonalactone, and eugenol were the most odour-active volatiles in aged rum. The aged rum was found to have an overall vanilla-like, dry, fruity aroma with additional oak wood, coconut, and caramel odour qualities [152].

**Figure 9:**
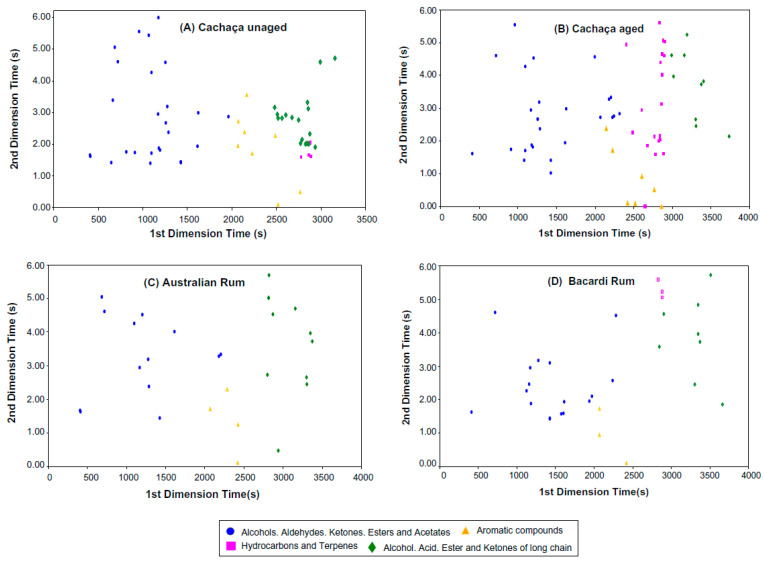
GC-GC/TOFMS peak apex plots of SPME headspace. Reprinted with permission from Cardeal and Marriot [115].

In another study, aroma analysis of spirit samples was conducted using cryogenic mass spectrometry. The results showed unique compounds in each identified class and that the aromatic compound region overlapped with the terpenes, but relatively few aromatics were noted [115]. In order to compare unaged and aged cachaça samples with rum samples, they used GC-GC analysis to create peak apex plots. These plots show the position of the maximum modulated peak in the 2D separation space. By comparing these plots, researchers found that unaged and aged cachaça samples are similar, but aged cachaça has more compounds, mainly terpenes, which give it a distinct flavour. Both cachaça plots differ from the rum plots, which have fewer volatile compounds, mainly hydrocarbons and terpenes. However, some compounds between rum and cachaça overlap, such as alcohols, aldehydes, esters, acetates, and aromatic compounds. These findings show that cachaça and rum are distinct spirits (Figure 9).

Sensory evaluation of cachaça has also been analysed, and the results showed that the best-classified cachaças were produced in copper stills and aged in oak casks. The intensity of yellow colour, wood flavour, sweetness, and fruit aroma positively correlated with the preference index [153].

The chemical composition of cachaças produced using yeast strains isolated from cachaça-producing regions was analysed. Cachaças produced using isolated yeast strains as starters displayed similar chemical compositions to spontaneous fermentation. Moreover, no contaminants specified by Brazilian legislation were detected. The study employed three selected yeast strains, and potential improvements in cachaça production were discussed [154].

Finally, a study used two different distillation processes to compare the chemical composition of two sugarcane spirits from the exact fermented most. The results showed that both distillation processes influenced the sugarcane spirits’ chemical quality and that two types of distillates with different quantitative chemical profiles were produced after the elimination of the fermentation step influence [155].

Yang et al. [156] analysed sugarcane juice’s aroma profiles and volatiles essential in cachaça production. The study found the TR-WAX column to be the best for detecting volatile compounds in sugarcane juice and categorized different sugarcane varieties into different groups based on their aromatic composition using PCA. Similarly, volatile and semi-volatile compounds in commercial rums were analysed using a headspace solid-phase microextraction procedure, successfully classifying more than 90% of the rums [157].

To classify a product as cachaça, several physicochemical analyses are performed, with the type of distillation device used influencing the chemical profile of the samples. Oliveira et al. [158] developed a method based on colour histograms and pattern recognition to analyse 122 samples of cachaça, 60 aged samples, and 62 non-aged samples adulterated. The study emphasized the importance of the type of distillation device in the chemical profile of the samples, as the content of DMS and esters was higher in the stainless-steel column samples.

Figure 9 displays peak apex plots of SPME headspace extracts from various spirit samples, including unaged and aged Brazilian cachaça, Australian rum, and Bacardi rum, analysed using GC-GC/TOFMS. Distinct symbols identify different compound classes: diamonds represent alcohols, aldehydes, ketones, esters, and acetic acid; squares represent aromatic compounds; triangles represent hydrocarbons and terpenes; and circles represent long-chain alcohols, acids, esters, and ketones. The plots reveal that compound groups are present in well-defined regions of the 2D separation space for each beverage, enabling the identification of differences and similarities among the spirit samples [115].

**Figure 10:**
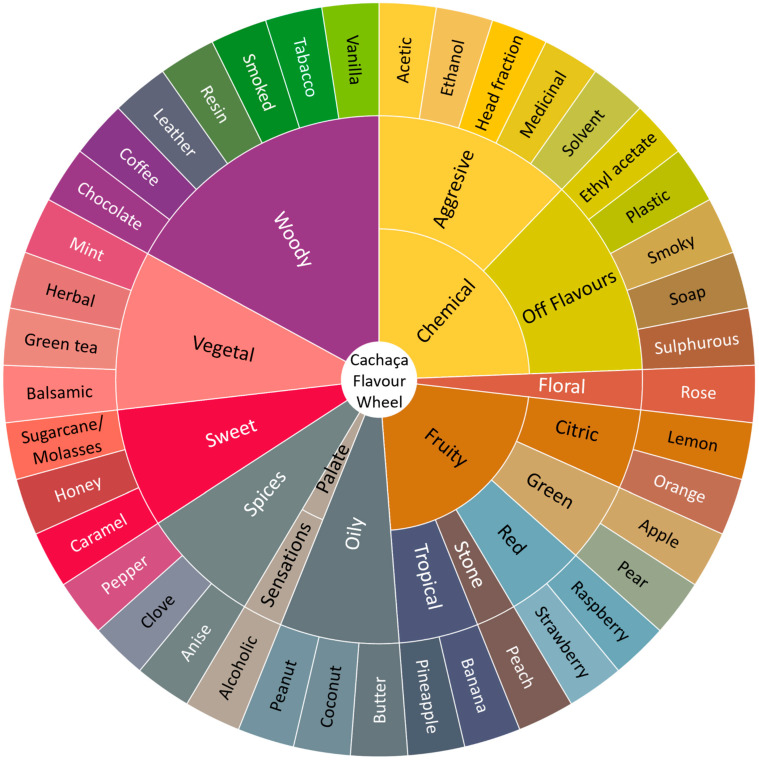
Cachaça flavour wheel.

A sensory wheel is an essential tool for understanding the complex and distinct flavour profile of cachaça, and it was created using carefully selected terms (Figure 10) [10,14,38,42,48,78,80,94,115,116,131,138,152,156,157,159] (see Appendix D). The sensory wheel is divided into three tiers, with the first tier categorizing the overall flavour profile into broader terms. The second tier contains more specific terminology within each category, while the third tier lists the odour descriptor. Terms that are similar in meaning are placed close to each other on the wheel, enhancing its usability. The first-tier descriptors have also been used in other alcoholic beverage lexicons for similar group flavours.

The aroma profile of cachaça is essential in producing high-quality cachaças and increasing their popularity in the global market. Research has shown that yeast strains significantly impact the creation of the distinctive aroma compounds in cachaça, and the type of distillation device used influences the chemical profile of the samples. Moreover, cachaça and rum have different aroma profiles, with specific odorants being more potent in cachaça than in rum [9,10,89,159,160,161]. Sensory evaluations have demonstrated that cachaças produced in copper stills and aged in oak casks are preferred, and the intensity of yellow colour, wood flavour, sweetness, and fruit aroma positively correlate with preference. Furthermore, analysis of volatile compounds in sugarcane juice and commercial rums provides valuable information for classifying cachaça and producing high-quality spirits.

Several factors, including yeast strains, bacteria presence, fermentation conditions, distillation, and maturation, influence the flavour of cachaça. The essential compounds responsible for the fruity-like aroma and alcoholic, wine-like, and whiskey-like notes in cachaça are esters and higher alcohols. Cachaça has two varieties: unaged (white or silver) and aged (yellow or gold). A spicy flavour typically characterizes aged cachaça due to higher levels of eugenol, 4-ethyl guaiacol, and 2,4-nonadienal [2].

Sensory tests are essential for the food and beverage industry, as they assess product acceptance, identify perceptible differences, and detect particularities that analytical procedures cannot detect. Ageing improves the sensory profile of cachaça, and further studies related to the sensory quality of cachaça are necessary, given the increase in demand and exportation. Standardising and developing sensory descriptors for cachaça is crucial to expand beverage qualification techniques and drive more significant quality investments. Appendix D provides information on important flavour volatiles in cachaça, including esters, ketones, acids, alcohols, acetals, phenolic compounds, and lactones, along with their typical concentration, flavour threshold, and sensory attributes. These compounds significantly impact distilled spirits’ overall flavour and aroma [2].

## 5. Contaminants during Cachaça Production

### 5.1. Vinasses

Cachaça and sugarcane spirit production generate significant vinasse, a potential environmental hazard. However, recent studies have shown that vinasse can be used as an alternative resource. Dos Reis et al. [162] evaluated the production of volatile compounds in vinasse and found that treating it with yeast can reduce its environmental impact by reducing biochemical and chemical oxygen demands (COD). The composition of the vinasses influences the effectiveness of the treatment.

Unfortunately, many cachaça producers struggle to meet quality standards established by Brazilian law. Menezes et al. [26] found that over 50% of analysed sugarcane spirit and cachaça samples did not comply with identity and quality standards. To further complicate matters, the mineral composition of vinasse from alembic cachaça can also vary depending on the management of the sugarcane. Mattos et al. [163] found that conventional management resulted in higher concentrations of some nutrients than organic management and that vinasse can be used in fertigation.

Despite these challenges, vinasse generated in cachaça production can still be helpful. Santos et al. [164] found it can produce *Candida utilis* CCT 3469 yeast cell biomass after preliminary purification. The chemical composition of the vinasse varied depending on the lignocellulosic materials used and the distillation process parameters.

While vinasse can be a potential environmental hazard, it can also be used as an alternative resource in cachaça production. The composition of vinasse can influence its effectiveness in reducing environmental impact, and the mineral composition of vinasse from alembic cachaça can vary depending on the management of the sugarcane. However, vinasse can still produce yeast cell biomass, a valuable resource. These findings provide valuable insights for producers and regulators in the cachaça industry.

### 5.2. Ethyl Carbamate (EC), PAH and Other Compounds in Cachaça Production

Cachaça has raised concerns about the potential health risks of EC in the beverage. EC, also known as urethane, is a compound commonly found in fermented food and beverage products and has been linked to carcinogenic properties [111].

#### 5.2.1. EC in Food and Beverage Products

In addition to cachaça, EC has been found in other fermented foods and beverages such as wine, sake, whiskey, brandy, bread, olives, and yoghurt [111]. In Brazil, studies have examined sugarcane brandies’ physicochemical and chromatographic components and found furfural concentrations below the maximum limit allowed [165].

Analysing EC in alcoholic beverages through the standard method requires a laborious extraction process using a diatomaceous earth column. As an alternative, researchers have investigated SPME, a solvent-free extraction technique. This method streamlines sample preparation and merges extraction and concentration into one efficient step. The applicability of the validated method using GC-MS combined with SPME for determining EC in cachaça demonstrated improved detection and quantification limits, offering a promising alternative to the traditional reference method [166].

**Figure 11:**
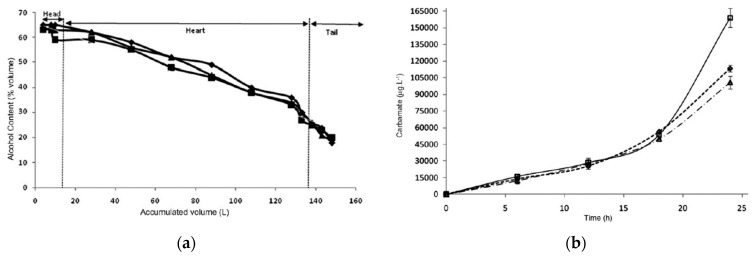
(**a**) Alcohol content as a function of distillate collected during wine distillation (fermented sugarcane juice); (**b**) concentration of EC in cane juice during the fermentation process to obtain cachaça. The different lines depicted in the graph correspond to successive repetitions of measurements taken during the distillation and fermentation process to produce cachaça. Reprinted with permission from Baffa et al. [167].

Another study determined EC in sugarcane spirits and found that EC was present in 70 of the 71 samples [25]. EC has been detected not only in cachaça but also in other fermented foods and beverages such as wine. Baffa et al. [167] emphasize the need to separate head and tail fractions from the heart fraction during distillation to ensure product quality (Figure 11a). In addition, they highlight that distillation in copper apparatus is necessary to guarantee good sensorial properties in the product, but copper contamination can occur. The GC-MS analyses of EC show that the compound is formed during sugar-cane fermentation and may result from yeast metabolism (Figure 11b). Therefore, more research is necessary to understand the pathway(s) involved in forming EC in fermented foods and beverages such as cachaça.

The European Commission’s CONTAM panel conducted a scientific opinion on the risks to human health from EC in food and alcoholic beverages. It concluded that the Margin of Exposure (MOE) of nearly 18,000 for exposure to EC in food, excluding alcoholic beverages, indicates a common concern [168]. A study estimated the lifetime cancer risk from EC in alcoholic beverages, including cachaça and tiquira, and found that EC poses a significant cancer risk for the alcohol-drinking population in Brazil [169].

Figure 11a illustrates the relationship between the volume of distillate collected during wine distillation and the alcohol content of the analysed samples. A consistent pattern in alcohol content across repetitions is observed. Head samples (up to 8 L) exhibit an alcohol content of approximately 65% ABV, heart samples (up to 128 L) show 35% ABV content, and tail samples (133 to 148 L) contain less than 20% ABV. After blending the heart fractions, the final cachaça product has an alcohol content of nearly 44% ABV, aligning with Brazilian regulations [167]. Figure 11b displays the EC concentration during the sugar cane juice fermentation. The graph reveals increased EC content throughout fermentation, peaking at 160 mg/L, suggesting EC production results from yeast metabolism. The EC content decreases during distillation, with the highest concentration in the head fraction (up to 8 L) and the lowest in the tail fraction (133 to 148 L). This highlights the importance of separating head and tail fractions from the heart fraction to ensure cachaça quality and minimize the risk of EC contamination [167].

#### 5.2.2. PAH in Cachaça Production

PAHs can also be formed during wood roasting, which is essential to consider as a potential source of PAH contamination in cachaça. PAHs in cachaça can pose health risks, as these compounds are genotoxic and carcinogenic [170].

Riachi et al. [19] reviewed data on EC and PAHs in Brazilian sugarcane spirits and the technologies to reduce their levels. The study found 16 PAHs in Brazilian sugarcane spirits and that the BaP content was two times higher in drinks from burned material than in drinks from unburned cane. The authors proposed that the precautionary principle of public health should be pursued to reduce the risk of EC and PAH contamination in sugarcane spirits from Brazil.

Bueno et al. [171] found that EC is a naturally occurring compound in fermented foods, and the formation of EC has been linked to the fermentation process. While EC is a naturally occurring compound in fermented foods and beverages, including cachaça, concerns have been raised about its potential health risks, particularly in alcoholic beverages. Further research is necessary to understand the pathways involved in forming EC in fermented products and identify strategies to reduce EC and PAH contamination in cachaça and other fermented products.

Menezes et al. [170] developed an innovative analytical method for detecting 16 PAHs in artisanal cachaça by combining direct immersion cooled fibre solid-phase microextraction (DI-CF-SPME) with gas chromatography-mass spectrometry (GC/MS). This simple, rapid, and cost-effective method demonstrated good precision, linearity, and sensitivity, enabling the researchers to measure trace levels of PAHs in 29 cachaça samples collected in Minas Gerais, Brazil. The benzo[a]pyrene equivalent (BaPeq) was quantified to assess health risks associated with PAH exposure. PCA and hierarchical cluster analysis (HCA) methodologies were employed to differentiate various sources of contamination in artisanal cachaça, offering valuable insights into the production process and quality control measures.

Souza et al. [172] investigated the occurrence of PAHs in cachaça stored in polyethylene terephthalate (PET) bottles and the impact of PET bottle storage on PAH levels. Cachaça samples from five producers in Minas Gerais were stored in PET bottles from three distinct manufacturers for up to eight months. High-performance liquid chromatography analyses revealed increased PAH concentrations over time in PET bottle-stored samples, with fluorene registering the highest concentration. These findings suggest that PET bottles can compromise cachaça quality and contribute to PAH contamination, highlighting the need to review legislation surrounding PET packaging used for beverage storage, given the carcinogenic nature of PAHs. This study provides valuable insights for regulatory bodies when considering plastic packaging for alcoholic beverages and managing associated consumer health risks.

Barbosa et al. [173] examined cachaça stored in PET packaging and revealed that the beverages contained high concentrations of chemicals outside the limits of Brazilian Quality Standards. They analysed 15 commercial cachaça samples stored in polymeric packaging to investigate the influence of storage on their physical-chemical quality and the presence of PAHs. The study found that all samples in PET packaging contained higher concentrations of PAHs than those reported in the literature for cachaça stored in alternative packaging types. PET packaging undergoes thermal processes during material synthesis and polymer moulding, which may contribute to forming PAHs and other contaminants. Of the analysed samples, 60% contained contaminant concentrations exceeding legal limits, and most had low ethanol concentrations.

#### 5.2.3. Formation of EC and Other Compounds

Its formation is driven by hydrogen cyanide and photochemically active substances, with various carbamyl compounds contributing to its formation [174]. In wine production, urea is believed to contribute primarily to the formation of EC.

Aresta et al. [175] reported that EC or urethane could be obtained industrially by the direct reaction of urea with ethanol. Iron ions in cachaça influence the reaction between ethanol and cyanate, and the distillation process also influences EC formation [176]. Double distillation reduces the EC content in sugarcane spirit by 97%, reducing the volatile cyanides and other nitrogen precursors [177].

Studies have shown that EC is formed in sugarcane spirit only after distillation, and the production process plays a significant role in EC formation [17]. Industrial cachaça was found to have higher levels of EC than homemade cachaça, indicating the need for further investigation into the production process’s role in EC formation. Additionally, they suggested that controlling the fermentation and distillation processes may be a way to reduce EC levels in cachaça [178].

In a study by Masson et al. [22], 71 sugarcane spirit samples were collected randomly from northern and southern Minas Gerais, Brazil, and analysed for compliance with Brazilian regulations. Results revealed that 9.85% of samples exceeded legal limits for acrolein, 21.00% for copper, and 8.85% for volatile acidity. Additionally, 26.71% of samples had alcohol concentrations below the minimum threshold. Cachaça, a distilled beverage made from fermented sugarcane broth, is unique to Brazil and represents a significant product in the nation’s agribusiness sector. The researchers developed an effective analytical method for determining acrolein levels in sugarcane spirits, which involved the formation of a dinitrophenylhydrazone derivative and analysis using high-performance liquid chromatography. In another study by Masson et al. [179], no correlations were observed between EC and alcohol content, acidity, or copper concentrations in the samples, supporting previous research [25].

A recent study by Alvarenga et al. [180] examined the correlation between the presence of acrolein with higher alcohols, glycerol, and acidity in cachaças. Cachaça displays significant variations in the concentration of acrolein, an aldehyde, among different beverages. They analysed 27 cachaça and sugarcane spirit samples acquired from retail stores across various Brazilian states. Phenolic compounds slightly correlated with acrolein formation, suggesting increased glycerol concentrations during beverage storage in wooden barrels. This finding indicates that acrolein may form under specific conditions. The authors attributed the presence of acrolein to the dehydration of glycerol, catalysed by metals and acids, and the contamination of the fermentation must occur by bacteria, such as *Bacillus amaracrylus* and *Lactobacillus colinoides*, which can synthesize enzymes that degrade glycerol. In addition, the chemical degradation and enzymatic conversion of glycerol, with a potential correlation to other compounds in the beverage, including butan-2-ol, propan-1-ol, and volatile acids.

Cravo et al. [181] investigated the link between the cyanogenic glycoside dhurrin in sugarcane and the formation of EC in cachaça. Five sugarcane varieties were collected from different regions in Brazil and subjected to two agroindustrial processing methods: stainless-steel column distillation and copper alembic distillation. EC formation is proposed to involve the cyanide ion (CN–), which forms via enzymatic action and thermal cleavage of cyanogenic glycosides, such as those potentially present in sugarcane. When oxidized, these glycosides form the cyanate ion (HCNO–), which reacts with alcohol to produce EC. Although none of the cachaça samples exceeded the EC limit, significant variations in the compound’s final concentration were observed among samples, highlighting the importance of understanding its formation mechanisms.

The presence of copper and cyanide has been linked to the formation of EC in cachaça. Bueno et al. [171] measured EC in sugarcane spirit for the first time and found that EC is a naturally occurring compound in fermented food. The relationship between copper in the beverage, total N in the juice, and EC incidence in cachaça requires further study for product commercialization [7].

The conditions used during the fermentation and storage of cachaça may also contribute to the formation of EC [182]. The researchers found that the storage of cachaça in oak barrels and glass containers influenced the formation of EC.

In summary, the formation of EC in cachaça production is a complex process driven by various chemical reactions, including hydrogen cyanide and carbamyl compounds. The production process, including fermentation, distillation, and storage, plays a significant role in EC formation, and controlling these processes may reduce EC levels in cachaça. Further studies are needed to fully understand the relationship between EC incidence in cachaça and factors such as copper concentration, total N in the juice, and storage conditions.

#### 5.2.4. EC Levels in Cachaça Production

With cachaça representing 87% of the volume production in the distilled beverage sector in Brazil, it is crucial to monitor the presence of contaminants such as ethyl EC and copper to maintain the quality of the beverage. Recent studies have measured the levels of EC and copper in cachaça produced under organic and conventional management systems [111].

Studies on EC levels in Brazilian sugarcane spirits have been conducted in various regions of Brazil, and the results have shown that EC is present in various sugarcane spirits, with concentrations ranging from 150 to 1500 μg/L [25]. Andrade-Sobrinho et al. [183] analysed 126 sugarcane spirit samples and found that EC’s presence varied between states. Double distillation has been shown to reduce EC levels in cachaça by 66.0% to 92.5% [177]. Similarly, whisky’s double distillation has also shown decreased EC content.

Several studies have examined the levels of EC in cachaça production in different regions of Brazil. Andrade-Sobrinho et al. [184] found that some brands exceed EC limits for ethanol. In Minas Gerais, researchers analysed the EC and other compounds in spirits produced in three state regions [185]. Another study of cachaças from Pernambuco State showed that the mean level of copper found for column still cachaças produced by blenders was lower than that for pot still ones [186].

Studies have found that EC concentrations in cachaça range from 20 to 960 μg/L, with 53% of samples having values above 210 μg/L [187]. However, all the aged cachaça samples analysed showed EC concentrations below the limit established by legislation [188]. The storage of cachaça in an oak barrel or glass vessel has also been shown to impact EC concentration, with the latter resulting in a significant increase in EC concentration [189].

Chreem et al. [190] analysed 120 samples of cachaça from 29 different brands and found that about 47% of the samples presented EC contents above 150 μg/L. The authors suggested that Brazilian authorities must require standardized production practices to reduce the amount of EC in cachaça. Zacaroni et al. [191] conducted a study to quantify the concentration of EC in cachaças aged in wooden barrels and stored in glass bottles. The researchers found no significant differences in the concentrations of EC in cachaças irradiated with sunlight and non-irradiated. They observed that the differences in the concentrations of EC presented a variation of 5.3–6.7%, suggesting that this compound is stable after its formation. The authors’ results showed that all the samples contained concentrations below the limit established by Brazilian legislation and were similar to the results of previous studies.

Mendonça et al. [192] found that EC was not detected in sugarcane juice, and the grinding process and cane variety did not seem to influence its formation. D’Avila et al. [182] analysed 13 unaged cachaça samples and found all contaminant levels below the legal ceiling. Guerreiro et al. [27] developed a new method based on QuEChERS and GC-MS Triple-Quadrupole to monitor EC in cachaça, finding only one sample above the limit allowed by Brazilian legislation.

The presence of EC in cachaça is undesirable for maintaining the quality of this popular distilled beverage in Brazil. Studies have measured the levels of EC in cachaça produced under organic and conventional management systems, with concentrations ranging from 20 to 960 μg/L and some brands exceeding EC limits for ethanol. Double distillation has been shown to reduce EC levels in cachaça, as has aged in oak barrels. However, storing cachaça in glass vessels has increased EC concentration. Brazilian authorities have been urged to require standardized production practices to reduce EC levels in cachaça. Despite this, many samples still contain EC concentrations above 210 μg/L. New methods for monitoring EC levels in cachaça have been developed, with promising results. Studies have also shown that EC is not present in sugarcane juice and that the grinding process and variety of cane do not seem to influence its formation. Overall, ongoing research on EC levels in cachaça production is essential for maintaining the quality and safety of this popular beverage.

#### 5.2.5. EC Formation during Distillation

The formation of EC during distillation was investigated by Riffkin et al. [193], who found that copper may act as a precursor or catalyst for ethanolysis. Battaglia et al. [194] found that EC was present in all vats from the second production day, but the differences disappeared before distillation. Exposure to sunlight was noted to cause a rise in EC concentration, and Aylott et al. [195] proposed a method for predicting final EC levels based on precursor levels in distilled spirits.

Bruno et al. [196] compared EC levels in cachaças produced using pots stills and distillation columns and found that EC levels were lower in cachaças produced using pots stills. Andrade-Sobrinho et al. [184] compared EC and cyanide content in sugarcane spirits produced using alembic distillation, and column distillation found that sugarcane spirits produced using alembic distillation had a lower content of EC.

Santiago et al. [197] investigated the stability of EC during the fermentation, distillation, and ageing of cachaça in oak and amburana barrels. They found that the concentration of EC remained stable throughout the production process, with a tendency for the concentration to stabilize in cachaça aged in oak and amburana barrels. However, further research is needed to understand the formation of EC during the distillation process.

Galinaro et al. [198] investigated the formation of EC from cyanate and ethanol and reported a substantial reduction in the concentration of EC when commercial samples of whiskey or cachaça were redistilled. They detected the presence of cyanate ions in freshly distilled sugarcane spirit and found that the decay of these species correlated with the formation of EC.

Rodrigues et al. [199] analysed samples from column and copper alembic distillation processes, revealing significant differences in chromatographic parameters. Column-distilled spirits exhibited higher concentrations of contaminants, from 5.63 to 7.00 mg/100 mL alcohol absolute, while copper alembic spirits had concentrations below legal limits. Furthermore, two samples contained EC concentrations exceeding legal limits, ranging from 245.31 to 235.53 μg/L. The research validates an analytical method for quantifying furfural and 5-HMF compounds formed or transferred during production.

Overall, these studies demonstrate the importance of understanding the factors that influence the formation of EC during cachaça production. Further research is needed to develop effective strategies for reducing EC levels in cachaça and ensuring the safety of this popular beverage.

## 6. Exploring the Current Research and Future Trends in Cachaça Production

Cachaça, a popular alcoholic beverage in Brazil, is made from sugarcane juice through fermentation and distillation. Producing high-quality cachaça requires strict adherence to specific parameters, including the raw material, type of yeast, fermentation, distillation, acidity, and content of copper, higher alcohols, aldehydes, EC, and methanol. However, there are concerns regarding the presence of EC, a potentially carcinogenic compound, in cachaça.

Several studies have been conducted to understand the formation and concentration of EC in cachaça, and the results suggest that the concentration of EC in cachaça is below the limit set by the legislation in Brazil. Further research is necessary to better understand the formation and concentration of EC in cachaça production and ensure this popular drink’s safety and quality.

In addition to EC, other contaminants such as pesticides and PAHs have been detected in cachaça. Studies have investigated the impact of ageing, wood cask type, and yeast strains on the quality and authenticity of cachaça. The findings of these studies provide valuable insights into the chemical profile of cachaça, including its phenolic content and ageing markers.

Recent research has explored vinasse, a by-product of cachaça production, as a resource in several applications, including the production of yeast cell biomass and as a medium for fertigation. The studies have provided valuable insights into the cachaça production process and may contribute to further improvements in the future.

Furthermore, studies on the physical and chemical quality, sensory evaluation, and extraction of compounds have also been conducted to better understand cachaça production and storage. Additionally, the detection of DMS and fatty esters in cachaça has been explored, improving the understanding of the physical characteristics of these beverages.

While cachaça production is an integral part of Brazilian culture and history, further research is necessary to address the concerns around contaminants and ensure the industry’s long-term sustainability and success. Future research trends in cachaça production include improving quality, ensuring product safety, and utilizing new technology and research for fermentation, distillation, and ageing.

In the production of cachaça, as with other distilled spirits, fermentation, distillation, and wood maturation are complex and involve (bio)chemical reactions that require a thorough understanding of quality control. Appreciating the intricacies of cachaça production necessitates delving into the understanding of the reactions that occur during the processing of raw material-derived musts through distillation. This includes complex chemical kinetics and equilibria involved in chemistry and wood maturation. To better understand cachaça production, it is essential to develop a deeper understanding of the driving forces that underlie the myriad of (bio)chemical and microbiological reactions involved.

In summary, cachaça production is evolving regarding quality, safety, innovation, presentation, and sustainability. To produce high-quality cachaça, quality control is necessary throughout the production process. Standardizing sensory descriptors is also crucial for driving more significant investments in quality and promoting cachaça’s global acceptance.

## 7. Conclusions

Cachaça, Brazil’s unique and traditional spirit, carries a rich cultural heritage and an evolving industrial landscape. Its production involves complex processes, from fermentation to ageing, which profoundly impact the final product’s quality and character. Understanding these processes is not merely a matter of tradition but also a vital part of contemporary research that carries industrial, environmental, and health implications.

- The role of yeast: yeast strains play a significant role in shaping the quality of cachaça, and further research is necessary to understand their impact on the fermentative process and their potential for biotechnological applications.
- Distillation factors: distillation is a critical aspect of cachaça production, and further study should investigate how various factors, including the raw material, type of yeast, fermentation, and chemical content, can affect the final quality.
- Chemical composition: monitoring the chemical composition of cachaça is crucial for ensuring its quality and identity. Development in production processes to improve flavour and consistency must be a focus of future research.
- Ageing in Brazilian wood casks: investigating the effects of different types of Brazilian wood casks on the final product can open new horizons in flavour profile and have potential industrial implications.
- Utilizing vinasse: exploring cachaça vinasse as an alternative yeast cell biomass production and fertigation resource provides a sustainable option that needs further research.
- Addressing health concerns: the presence of EC in cachaça is alarming due to its potential carcinogenic effects. Future studies must focus on understanding its formation, concentration, and reduction.
- Industry sustainability: overall, continued research is necessary to ensure the long-term sustainability and success of the cachaça industry, with particular emphasis on safety concerns, quality improvement, and the application of new technology and research in fermentation, distillation, and ageing.

## Figures and Tables

**Table 1:** Classification and labelling terminology for cachaça [4].

Classification	Name	Description	Labelling
Distillation process	*Cachaça de alambique*	when it is exclusively and entirely produced in a copper still and obtained by distilling fermented sugarcane juice.	Optional
	*Cachaça*	when it is produced by a different distillation method or a mixture of cachaças from other methods.	
Ageing process	*Cachaça envelhecida*	which is aged in a wooden container with a maximum capacity of 700 L for at least one year and contains at least 50% of its volume aged in the wood.	Required
	*Cachaça armazenada*	which is stored in a wooden container but does not meet the criteria for ageing as defined in the current Standards of Identity and Quality and other relevant administrative acts.	
	*Cachaça*	which is packaged in a suitable container and does not meet the criteria for ageing or storage in wood as defined in the current Standards of Identity and Quality and other relevant administrative acts.	
Sugar content	*Cachaça adoçada*	which is a beverage with added sugars in quantity greater than 6 g/L and less than 30 g/L.	Required
	*Cachaça*	which may contain added sugars in quantity equal to or less than 6 g/L	

**Table 2:** Minimum and maximum tolerable limits for different components of cachaça according to Brazilian legislation [4].

Parameter	Unit	Min	Max
Components			
Alcoholic graduation	% ABV at 20 °C	38	48
Volatile acidity, as acetic acid	mg/100 mL of anhydrous alcohol		150
Total esters, as ethyl acetate	mg/100 mL of anhydrous alcohol		200
Total aldehydes, in acetaldehyde	mg/100 mL of anhydrous alcohol		30
Sum of Furfural and Hydroxymethylfurfural	mg/100 mL of anhydrous alcohol		5
Higher alcohols *	mg/100 mL of anhydrous alcohol		360
Congeners	mg/100 mL of anhydrous alcohol		650
Total phenolic compounds (for aged cachaça)			
Total sugars (for cachaça)	g/L (as glucose)		≤6
Total sugars (for sweetened cachaça)	g/L (as glucose)	>6	<30
Contaminants			
Methanol	mg/100 mL of anhydrous alcohol		20
Ethyl carbamate (EC)	μg/L		210
Acrolein (2-propenal)	mg/100 mL of anhydrous alcohol		5
sec-Butyl alcohol (2-butanol)	mg/100 mL of anhydrous alcohol		10
n-Butyl alcohol (1-butanol)	mg/100 mL of anhydrous alcohol		3
Copper	mg/L		5

* Higher alcohols: sum of isobutyl alcohols (2-methyl propanol), isoamyl alcohols (2-methyl-1-butanol and 3-methyl-1-butanol) and n-propyl alcohol (1-propanol).

**Table 3:** Impact of various fermentation strains on cachaça production parameters and effects [8,14,15,37,38,40,46,47,48,49,50,51,52].

Strain	Quality Parameters	Effects
*Bacillus amaracrylus*	Fusel alcohols, aroma, flavour development	Increased production of fusel alcohols contributes to aroma and flavour development. Increases acidity. Contributes to flavour development.
*Candida apicola*	Ester production, aroma	An increase in ester production contributes to the fruity aroma. Increases acidity. Contributes to flavour development. Instrumental in the production of esters and higher alcohols contributing to the aroma of cachaça.
*C. famata*	Higher alcohols, flavour complexity	Enhanced production of higher alcohols contributes to flavour complexity. Increases acidity. Contributes to flavour development.
*C. guilliermondii*	Glycerol production, mouthfeel, body	Improved glycerol production enhancing mouthfeel and body. Increases acidity. Contributes to flavour development.
*D. bruxellensis*	Phenolic compounds, flavour complexity	Increased production of phenolic compounds contributes to flavour complexity. Increases acidity. Contributes to flavour development.
*Hanseniaspora guillierdii*	Acidity, flavour development	Increases acidity. Contributes to flavour development. Responsible for creating fruity and floral aromas due to increased production of higher alcohols.
*Lachancea mirantina*	Acidity, flavour development	Increases acidity. Contributes to flavour development—aids in producing esters and higher alcohols, adding to the aroma of cachaça.
*Lactobacillus colinoides*	Lactic acid, acidity, flavour development	The production of lactic acid and other organic acids contributes to acidity. Increases acidity. Contributes to flavour development.
*Lactobacillus hilgardii*	Acidity, volatile acidity, flavour development	It increased acidity and volatile acidity. Increases acidity. Contributes to flavour development. Produces lactic acid, adding to the acidity and flavour profile of cachaça.
*Lactobacillus* spp.	Acidity, volatile Acidity, flavour, aroma, fermentation behaviour	Increase in acidity and volatile acidity due to the production of lactic acid and other organic acids. Increases acidity. Contributes to flavour development. Crucial role in preventing bacterial contamination in the fermentation process. Contributes to unique smell, taste, and flavour development due to metabolite and volatile substance production. Impacts the overall fermentation behaviour of the mixture.
*Lactococcus lactis*	Acidity, volatile acidity, aroma	Enhanced production of diacetyl contributing to buttery aroma. Increase in acidity and volatile acidity. Increases acidity. Contributes to flavour development. Contributes to acidity and flavour development through the production of lactic acid.
*M. caribbica*	Higher alcohols, flavour complexity, alcohol content	Improved production of higher alcohols contributes to flavour complexity. Increases alcohol content. Decreases acidity. Contributes to flavour development.
*Meyerozyma guilliermondii*	Fruity esters, aroma profile, flavour development	Improved production of fruity esters enhancing aroma profile. Increases acidity. Contributes to flavour development. It is known for its role in producing esters that enhance the aroma of cachaça.
*Pichia caribbica*	Volatile compounds, aroma complexity	Enhanced production of volatile compounds contributing to aroma complexity. Increases acidity. Contributes to flavour development.
*Pichia fermentans*	Esters, fruity aroma, flavour development	Enhanced production of esters contributing to the fruity aroma. Increases acidity. Contributes to flavour development. It helps produce esters that contribute to the aroma of cachaça.
*S. cerevisiae*	Ethanol production, aroma and flavour profile, fermentation efficiency, alcohol content, fermentation kinetics, residual sugar levels	Increase in ethanol production. Improvement in aroma and flavour. Enhanced fermentation efficiency. Increases alcohol content. Decreases acidity. Contributes to flavour development. Known for its high ethanol production, which forms the basis of cachaça’s alcohol content. Increases alcohol content and enhances aroma profile due to increased ethanol production and improved fermentation kinetics. Reduces residual sugar levels by minimizing the risk of stuck fermentation.
*Schizosaccharomyces pombe*	Fusel alcohols, aroma, flavour development	Increased production of fusel alcohols contributes to aroma and flavour development. Increases acidity. Contributes to flavour development—aids in producing higher alcohols and esters that add to the aroma of cachaça.

## Data Availability

All the data is found in the article and the references.

## Abbreviations

ABV	Alcohol By Volume
BaP	Benzo[a]pyrene
BaPeq	Benzo[a]pyrene equivalent
°Bx	Brix or percentage of soluble solids
COD	Chemical Oxygen Demands
CRT	Tequila Regulatory Council
DI-CF-SPME	Direct Immersion Cooled Fibre Solid-Phase Microextraction
DMS	Dimethyl Sulfide
EC	Ethyl Carbamate
ESI–MS	Electrospray Ionization Mass Spectrometry
GC-BID	Gas Chromatography with Barrier Ionization Discharge
GC-FID	Gas Chromatography-Flame Ionization Detector
GC-MS	Gas Chromatography-Mass Spectrometry
GC×GC	Two-dimensional Gas Chromatography
GAE	Gallic Acid Equivalent
GI	Geographical Indication
GMP	Good Manufacturing Practices
GYMP	Growth medium containing Glucose, Yeast extract, Malt extract, and Peptone
HACCP	Hazard Analysis and Critical Control Points
HCA	Hierarchical Cluster Analysis
HMF	Hydroxymethylfurfural
HPLC/UV	High-Performance Liquid Chromatography/Ultraviolet
IBRAC	Brazilian Institute of Cachaça
ITS	Internal Transcribed Spacer
MAPA	Brazilian Ministry of Agriculture, Livestock, and Supply
MI	Maturity Index
MOE	Margin of Exposure
PBDAC	Brazilian Program for the Development of Cane Spirit, Caninha or Cachaça
PAHs	Polycyclic Aromatic Hydrocarbons
PCA	Principal Component Analysis
PCR	Polymerase Chain Reaction
PET	Polyethylene Terephthalate
QDA	Quantitative Descriptive Analysis
QuEChERS	Quick, Easy, Cheap, Effective, Rugged, and Safe
RAPD-PCR	Random Amplification of Polymorphic DNA—Polymerase Chain Reaction
SPME	Solid-Phase Microextraction
TFL	Trifluoroleucine
TOFMS	Time-of-Flight Mass Spectrometry
TR-WAX	Type of chromatography column
UV-Vis	Ultraviolet-Visible
VOCs	Volatile Organic Compounds

## Appendix A. Caipirinha Cocktail Recipe

One popular way to enjoy cachaça is in a caipirinha cocktail based on the IBA (International Bar Association). Here is how to make it:

Ingredients:

- 60 mL cachaça
- One lime, cut into small wedges
- Four teaspoons (16 g) white cane sugar
- Method:
- Place the lime wedges and sugar into an old-fashioned double glass.
- Gently muddle the lime and sugar together to release the lime juice and dissolve the sugar.
- Fill the glass with cracked ice.
- Add the cachaça to the glass.
- Stir gently to combine the ingredients.
- Garnish: none needed.

Note: for a variation of this cocktail, try using vodka or rum instead of cachaça to make a caipiroska or caipirissima, respectively.

Enjoy this refreshing and traditional Brazilian cocktail!

## Appendix B. Basic Calculation Scheme on Cachaça Production

### Appendix B.1. Dilution of Sugarcane Juice for Fermentation

This section outlines the procedure for diluting sugarcane juice for the onset of fermentation, as an example:

Start with sugarcane juice at an initial concentration of 22 °Bx.

Aim to achieve 100 L of must at 15 °Bx.

Calculate the specific gravity (SG) of the sugarcane juice at 15 °Bx using the following equation; the range of application for these equations are SG between 1.00 and 1.16, % ABV between 0 and 20 and Brix between 0 and 35:

**SG**	**% ABV**	**°Bx**
$SG={\left(0.99958251-0.0037958995\cdot {}^{\circ}Bx\right)}^{-1}$	$\%\;ABV={\left[0.88411955\cdot {{}^{\circ}Bx}^{0.5}-0.61771014\right]}^{2}$	
	$\%\;ABV=134.65985\cdot SG\cdot \ln\left(SG\right)-0.60945368$	${}^{\circ}Bx=263.15882-263.25586\cdot {SG}^{-1}$

If the temperature of the juice is different than 20 °C, a temperature correction factor for SG should be applied using the following equation:

$$SG_{c}=SG_{m}\cdot \left(-1.35666446\cdot 10^{-8}T^{3}+5.88216338\cdot 10^{-6}T^{2}-2.02881424\cdot 10^{-5}T+9.98161431\cdot 10^{-1}\right)$$

where T is the temperature in Celsius, $SG_{m}$ and $SG_{c}$ are the measured and the corrected SG, respectively.

The SG is determined to be 1.061. Multiply the SG by the volume to obtain the mass of the juice:

100L·1.061=106.1kg

To determine the final volume of the solution, use the formula:

$$C_{1}\cdot V_{1}=C_{2}\cdot V_{2}$$

22°Bx·M1=15°Bx·106.1kg

Solve for M1:M1 = 72.34 kg of sugarcane juice at 22 °Bx

Convert the mass to volume using the SG of sugarcane juice at 22 °Bx (1.0918):

$$V=\frac{72.34}{1.0919}=66.25\;L\;of\;sugarcane\;juice\;at\;22\;{}^{\circ}Bx$$

Therefore, adding 33.75 L of water (100–66.25) is necessary to obtain 100 L of must at 15 °Bx.

### Appendix B.2. Fermentation Efficiency

Fermentation efficiency (E) is a critical parameter in cachaça production, as it directly influences the ethanol yield. It is calculated by comparing the actual ethanol produced (% *v*/*v*) to the theoretical ethanol (% *v*/*v*), expressed as a percentage. The equation for fermentation efficiency is:

$$E(\%)=\frac{Real\;Volume}{Theoretical\;Volume}\times 100$$

We consider the stoichiometry of sucrose (C_12_H_22_O_11_, 342 g) conversion to ethanol (4 × C_2_H_6_O, 184 g) to calculate the theoretical yield. The theoretical yield is 0.538 g of ethanol per gram of sucrose (184 g ethanol/342 g sucrose).

Sugarcane juice with 16 °Bx density is 1.0654, resulting in 1065.4 kg of must for a 1000 L volume. With 0.16 fraction of sucrose in the must, we have 170.46 kg of sucrose. Assuming 100% of soluble solids as sucrose, the theoretical volume of ethanol can be calculated as follows:

$$Theoretical\;Volume=\frac{\left(170.46\;kg\;sucrose\times 0.538\right)}{0.78934(ethanol\;SG)}=116.2\;L\;of\;ethanol(91.71\;kg)$$

For the real volume, suppose the fermentation process yielded 8.5% ethanol:

$$Real\;Volume=\frac{8.5\%\;ethanol}{100}\times 1000\;L\;juice=85\;L\;of\;ethanol$$

Finally, the fermentation efficiency is calculated as follows:

$$E\left(\%\right)=\frac{85\;L}{116.2\;L}\times 100=73.15\%$$

In this example, the fermentation efficiency of 73.15% indicates that 73.15% of the sugars were converted into alcohol. A higher efficiency represents a more negligible difference between the theoretical and practical volumes and a higher sugar-to-alcohol conversion rate.

### Appendix B.3. Distillation Efficiency

The first step is to determine the % ABV in the must using the following expression based on the initial ($SG_{i}$) and final ($SG_{f}$) specific gravity:

$$\%\;ABV=95.819\cdot SG_{f}\cdot \left(\frac{SG_{i}-SG_{f}}{1.775-SG_{i}}\right)$$

Distillation efficiency (DE) is a critical parameter in cachaça production, as it directly influences ethanol recovery. It is calculated by comparing the volume of ethanol recovered, or the volume of absolute ethanol distilled (% *v*/*v*), to the ethanol present in the fermented must (% *v*/*v*), expressed as a percentage. The equation for fermentation efficiency is:

$$DE(\%)=\frac{Volume\;of\;ethanol\;distilled}{Volume\;of\;ethanol\;fermented\;must}\times 100$$

We have 1000 L of fermented must with 8.5% ABV:

1000·0.085=85Lofethanolfermentedmush

It is placed in the still, and all the distillate is collected until the spirit exiting the spirit pipe reads 1% ABV. During the collection of the head, heart and tails, we have Table A1.

**Table A1:** Distillation results.

	Total Volume	Ethanol Content	Ethanol Volume	Fraction of Total Volume	Total Ethanol Fraction
	(L)	(% ABV)	(L)	(%)	(% ABV)
Wine (must)	1000.00	8.50	85.00	100.00	100.00
Head	13.08	65.00	8.50	1.31	10.00
Heart	141.67	48.00	68.00	14.17	80.00
Tail	21.25	20.00	4.25	2.12	5.00
Vinasses	824.01	0.52	4.25	82.40	5.00

Based on Table A1, it is necessary to determine the total ethanol distilled and the ethanol content as follows:

Ethanolvolume=head+heart+tail=13.08+141.67+21.25=176L

$$Ethanol\;content=\frac{head+heart+tail}{Ethanol\;volume}=\frac{13.08\cdot 0.65+141.67\cdot 0.48+21.25\cdot 0.20}{176}\cdot 100=45.88\%\;ABV$$

Therefore, after distillation, 176 L at 45.88% ABV.

176·0.4588=80.75Lofethanoldistilled

The efficiency of the distillation is:

$$DE=\frac{80.75\;L}{85\;L}\cdot 100=95\%$$

In this example, the distillation efficiency of 95% indicates that 95% of the ethanol present in the fermented must was recovered during the distillation process. A higher distillation efficiency represents a more efficient separation of the ethanol from the fermented must and a higher yield.

Overall Yield:

The overall yield of cachaça production is the product of the fermentation and distillation efficiency. In this example, the overall yield (OY) is calculated as follows:

OY(%)=E×DE

OY=73.15%×95%=69.49%

In this example, the overall yield of 69.49% indicates that 69.49% of the initial sugar content in the sugarcane juice was converted into ethanol and recovered during the distillation process. A higher overall yield represents a more efficient use of the sugar content in the sugarcane juice and a higher ethanol yield.

### Appendix B.4. Formulas for Calculating Volume, Surface Area, and Ageing Time in Barrels

Barrels commonly have a bulging design, meaning they do not represent perfect cylinders. Based on this geometry, the formulas to calculate a barrel’s volume and internal surface area are presented below.

The volume of a barrel ($V_{barrel}$, m^3^):

$$V_{barrel}=\frac{\pi }{4}\cdot H\cdot D_{m}^{2}$$

where:

H: Height of the barrel (m)

D: Diameter at the bulging (widest) part of the barrel (m)

$D_{e}$: Diameter at the end (lid) of the barrel (m)

$D_{m}$: Average diameter of the barrel ($=\left(D+D_{e}\right)/2$) (m)

The internal surface area of a barrel ($A_{barrel}$, m^2^):

$$A_{barrel}=\pi \cdot D_{m}\left(\frac{D_{m}}{2}+H\right)$$

Specific surface area (SE, m^−1^):

$$SE=\frac{A_{barrel}}{V_{barrel}}=\frac{2\cdot \left(D_{m}+2\cdot H\right)}{D_{m}\cdot H}$$

Aging Time (TE, days):

The ageing time is inversely proportional to the specific surface area and can vary depending on the type of wood used. According to Brazilian legislation for barrels of 700 L and a specific surface area of 6.235 m^−1^, the ageing time in days is calculated as:

$$TE=365\cdot \frac{6.235}{SE}$$

## Appendix C. Inoculum for the Fermentation Process

Caipira ferment:

Steps to produce Inoculum for the Fermentation Process:

1. Gather ingredients: acquire 3 kg of rice bran, 4 kg of cornmeal, 1 kg of biscuits, and enough lemon or sour orange peel to form a paste.
2. Prepare the inoculum: combine the ingredients in a bag and mix to form a paste. Leave the mixture to rest for 12 to 24 h until cracks appear on the surface.
3. Add diluted cane juice: introduce diluted cane juice (1:1) to the mixture until submerged, cover it with a cloth and leave it to rest for an additional 24 h.
4. Repeat the addition of cane juice: upon observing effervescence in the mixture, add two to five times more diluted cane juice and let it rest for 24 h. Repeat this step until the inoculum reaches 0.2% of the volume of the primary fermentation tank.
5. Prepare inoculum directly in the primary fermentation tank: the inoculum can also be prepared directly in the primary fermentation tank.
6. Obtain the initial volume of inoculum: the initial volume obtained from the “fermento caipira” process is usually equivalent to 20% of the volume of the primary fermentation tank when settled.

CA-11

Steps to produce the inoculum for the fermentation process using yeast CA-11:

1. Rehydrate yeast cells: mix 1 kg of yeast with 10 L of water at 40 °C and constantly agitate for 1 h.
2. Initial juice addition: add 10 L of juice with 8 °Bx and 35 °C to the fermentation tank. Use a compressor to aerate the system.
3. The second juice addition: once the Brix inside the tank reaches 2–3 °Bx, add 40 L of juice with 10 °Bx and 35 °C. Continue aeration.
4. Third juice addition: wait for the Brix inside the tank to reach 2–3 °Bx, and add 70 L of juice with 12 °Bx.
5. Transfer to fermentation tank: when the Brix inside the tank reaches 2–3 °Bx, transfer the contents to the primary fermentation tank, constantly agitating to remove all yeast mass. Add 150 L of juice with 14 °Bx and 30 °C. Wait for the Brix to reduce to 2–3 °Bx.
6. Feeding process: start feeding the tank with juice at 15 °Bx, gradually adding the juice so that the Brix inside the tank does not exceed 7 °Bx. Wait for the Brix to reduce to 3 °Bx before adding the next portion of juice. Continue feeding until the total volume of juice (1000 L) has been added.

## Appendix D. Cachaça Flavour Wheel

**#**	**Volatile Compound Name (Tier 4)**	**IUPAC Name**	**Compound Category**	**CAS**	**Odour Type**	**Odour Descriptor**	**Tier1**	**Tier2**	**Tier3**	**Tier1**	**Tier2**	**Tier3**	**Author**
1	(-)-Cubenol	(1S,4R,4aR,8aR)-4,7-dimethyl-1-propan-2-yl-2,3,4,5,6,8a-hexahydro-1H-naphthalen-4a-ol	Alcohols	21284-22-0	Spicy	Spicy, herbal, tea, green tea	Spices		Clove	Vegetal		Green tea	[116]
2	(±)-Menthone	5-methyl-2-propan-2-ylcyclohexan-1-one	Ketones	89-80-5	Minty	Minty	Vegetal		Mint				[116]
3	(E)-2-Nonenal	(E)-non-2-enal	Aldehydes	18829-56-6	Fatty	Fatty, green, cucumber, aldehydic, citrus	Oily		Peanut	Fruity	Citric	Lemon	[10]
4	(E)-3-Hexen-1-ol	(E)-3-hexen-1-ol	Alcohols	928-97-2	Green	Green, cortex, privet, leafy, floral, petal, oily, earthy	Vegetal		Herbal	Floral		Roses	[48]
5	(E)-a-Bergamotene	(1S,5S,6R)-2,6-dimethyl-6-(4-methylpent-3-en-1-yl)bicyclo[3.1.1]hept-2-ene	Terpene	13474-59-4	Woody	Woody, warm, tea	Woody		Resin				[116]
6	(E)-b-Farnesene	(6E)-7,11-dimethyl-3-methylidenedodeca-1,6,10-triene	Terpene	18794-84-8	Woody	Woody, citrus, herbal, sweet	Woody		Resin	Fruity	Citric	Orange	[78]
7	(E)-Ethyl dec-4-enoate	(E)-Ethyl dec-4-enoate	Esters	76649-16-6	Green	Green, fruity, waxy, cognac	Vegetal		Herbal				[94]
8	(E)-Geranyl acetone	(5E)-6,10-dimethylundeca-5,9-dien-2-one	Ketones	3796-70-1	Floral	Fresh, green, fruity, waxy, roses, woody, magnolia, tropical, pear, guava.	Floral		Roses	Sweet		Honey	[116]
9	(E,E)-Farnesol	(2E,6E)-3,7,11-trimethyldodeca-2,6,10-trien-1-ol	Alcohols	106-28-5	Floral	Floral, sweet, lily, waxy	Floral		Roses	Fruity	Citric	Orange	[48,116]
10	(Z)-Nerolidol	(6Z)-3,7,11-trimethyldodeca-1,6,10-trien-3-ol	Alcohols	3790-78-1	Waxy	Waxy, floral	Oily		Butter				[116]
11	10-Undecen-1-ol	10-undecen-1-ol	Alcohols	112-43-6	Citrus	Fresh, floral, waxy, ozone, clean, citrus	Fruity	Citric	Lemon				[94,115]
12	1-Dodecanol	1-Dodecanol	Alcohols	112-53-8	Waxy	Earthy, soapy, waxy, fatty, honey, coconut	Oily		Butter				[48]
13	2,3-Butane diol	2,3-butanediol	Diol	513-85-9	Creamy	Fruity, creamy, buttery	Oily		Butter	Sweet		Honey	[134]
14	2,4-Nonadienal	Nona-2,4-dienal	Aldehydes	6750-03-4	Green	Fatty, green, cucumber, fruit, tropical fruit	Vegetal		Herbal	Fruity	Tropical	Pear	[10]
15	2,5-Diethyl tetrahydrofuran	2,5-diethyloxolane	Ether	41239-48-9	Herbal	Sweet, herbal, caramellike	Vegetal		Herbal	Sweet		Caramel	[116]
16	2-Butanol	Butan-2-ol	Alcohols	78-92-2	Fruity	Sweet, apricot	Fruity	Tropical	Apple	Sweet		Honey	[134]
17	2-Ethyl-1-hexanol	2-ethylhexan-1-ol	Alcohols	104-76-7	Citrus	Citrus, fresh, floral, oily, sweet	Fruity	Citric	Lemon	Sweet		Honey	[48]
18	2-Heptanol	Heptan-2-ol	Alcohols	543-49-7	Citrus	Fresh, lemongrass, herbal, sweet, floral, fruity, green	Fruity	Citric	Lemon	Vegetal		Herbal	[94,115,116,134]
19	2-Methoxy-4-vinyl phenol	4-ethenyl-2-methoxyphenol	Phenol	7786-61-0	Spicy	Spicy, clove, carnation, phenolic, peppery, smoky, woody, powdery	Spices		Clove				[10,134]
20	2-Methyl butyl acetate	2-Methylbutyl acetate	Esters	624-41-9	Fruity	Fruit, overripe, sweet, banana, ripe, tropical fruit	Sweet		Honey	Fruity	Citric	Lemon	[94,115]
21	2-Methyl-1-butanol	2-methylbutan-1-ol	Alcohols	137-32-6	Ethereal	Ethereal, fusel, alcoholic, fatty, greasy, winey, whiskey, leathery, cocoa, Fish oil, green, malt, onion, wine	Palate	Sensations	Alcoholic	Woody		Leather	[38,94,115,116]
22	2-Nonanol	Nonan-2-ol	Alcohols	628-99-9	Waxy	Waxy, green, creamy, citrus, orange, cheesy, fruity	Oily		Butter				[94,115]
23	2-Nonanone	Nonan-2-one	Ketones	821-55-6	Fruity	Fresh, sweet, green, weedy, earthy, herbal, fruity, waxy, soapy, cheesy, coconut	Sweet		Honey	Fruity	Citric	Lemon	[48]
24	2-Octanol	Octan-2-ol	Alcohols	123-96-6	Spicy	Fresh, spicy, green, woody, herbal, earthy	Spices		Clove	Vegetal		Herbal	[94,115]
25	2-Undecanol	Undecan-2-ol	Alcohols	1653-30-1	Waxy	Fresh, waxy, cloth, laundered cloth, sarsaparilla	Oily		Butter				[94,115]
26	2-Undecanone	Undecan-2-one	Ketones	112-12-9	Fruity	Waxy, fruity, creamy, fatty, orris, floral, ketonic, pineapple	Sweet		Honey	Floral		Roses	[94,115]
27	3-Methyl-1-pentanol	3-methylpentan-1-ol	Alcohols	589-35-5	Fermented	Fusel, cognac, winey, cocoa, green, fruity, pungent	Palate	Sensations	Alcoholic	Vegetal		Herbal	[48,134]
28	4-Ethyl guaiacol	4-Ethyl-2-methoxyphenol	Phenol	2785-89-9	Spicy	Spicy, smoky, bacon, phenolic, clove, medicinal, woody, sweet, vanilla	Spices		Clove	Woody		Vanilla	[10,94,115]
29	4-Ethyl phenol	p-Ethylphenol	Phenol	123-07-9	Smoky	Phenolic, castoreum, smoky, guaiacol	Woody		Smoked	Chemical	Off flavours	Smoky	[48]
30	4-Methyl guaiacol	2-methoxy-4-methylphenol	Phenolic Compounds	93-51-6	Spicy	Vanilla, clove, spicy, phenolic, medicinal, leathery, woody, smoky, burnt, candy,	Spices		Clove	Woody		Leather	[2]
31	5-Hydroxymethyl furfural	5-(hydroxymethyl)furan-2-carbaldehyde	Aldehydes	67-47-0	Fatty	Fatty, buttery, musty, waxy, caramellike	Oily		Butter	Sweet		Caramel	[42]
32	5-Methyl hexanoic acid	5-methylhexanoic acid	Acid	628-46-6	Fatty	Fatty, cheesy, oily, fruity	Oily		Peanut	Sweet		Honey	[116]
33	Acetal	1,1-Diethoxyethane	Ether	105-57-7	Ethereal	Green, nutty, earthy, sweet, vegetable, Ethereal	Spices		Anise	Sweet		Honey	[94,115,116]
34	Acetaldehyde	Acetaldehyde	Aldehydes	75-07-0	Ethereal	Pungent, ethereal, aldehydic, fruity, musty, green apple	Palate	Sensations	Alcoholic	Sweet		Honey	[14,42,48,134]
35	Acetic acid	Acetic acid	Acid	64-19-7	Acidic	Sharp, pungent, sour, vinegar	Chemical	Aggressive	Acetic				[14,38,42,48,78,134]
36	Acetone	Propan-2-one	Ketones	67-64-1	Solvent	Solvent, ethereal, apple, pear	Chemical	Aggressive	Solvent	Fruity	Tropical	Pear	[134]
37	Acetophenone	1-phenylethanone	Ketones	98-86-2	Floral	Sweet, pungent, hawthorn, mimosa, almond, acacia, chemical, cherry, marzipan, coumarin, almond, nutty, heliotrope, vanilla	Floral		Roses	Sweet		Molasses	[42]
38	Acrolein	Acrolein	Aldehydes	107-02-8	Fruity	Almond, cherry	Fruity	Tropical	Apple	Sweet		Honey	[134]
39	a-Farnesene	(3E,6E)-3,7,11-trimethyldodeca-1,3,6,10-tetraene	Terpene	502-61-4	Woody	Citrus, herbal, lavender, bergamot, myrrh, neroli, green, woody, vegetable, floral	Woody		Resin	Fruity	Citric	Lemon	[116]
40	Amyl alcohol	Pentan-1-ol	Alcohols	71-41-0	Fermented	Fusel, oily, sweet, balsamic, pungent, fermented, bready, yeasty, fusel, winey, solvent	Palate	Sensations	Alcoholic	Chemical	Aggressive	Solvent	[14,38]
41	Anisole	Anisole	Ether	100-66-3	Phenolic	Phenolic, gasoline, ethereal, anise	Chemical	Aggressive	Medicinal				[94,115]
42	a-Terpeniol	2-(4-methyl-1-cyclohex-3-enyl)propan-2-ol	Alcohols	98-55-5	Terpene	Pine, terpene, lilac, citrus, woody, floral, resinous, cooling, lemon, lime	Woody		Resin	Floral		Roses	[48]
43	b-Damascenone	(E)-1-(2,6,6-trimethyl-1-cyclohexa-1,3-dienyl)but-2-en-1-one	Ketones	23696-85-7	Floral	Natural, sweet, fruity, roses, plum, grape, raspberry, sugar, woody, earthy, green, floral, apple	Floral		Roses	Sweet		Honey	[94,115]
44	b-Damascone	(E)-1-(2,6,6-Trimethyl-1,3-cyclohexadien-1-yl)-2-buten-1-one	Ketones	35044-68-9	Fruity	Floral, black currant, plum, roses, honey, tobacco	Sweet		Honey	Floral		Roses	[116]
45	Benzaldehyde	Benzaldehyde	Aldehydes	100-52-7	Fruity	Sharp, sweet, bitter, almond, cherry, fruity, powdery, nutty, maraschino cherry	Fruity	Tropical	Apple	Sweet		Honey	[42,48,134]
46	Benzoic acid	Benzoic acid	Acid	65-85-0	Balsamic	Balsamic, urine	Vegetal		Balsamic				[38]
47	Bisabolene	1-methyl-4-(6-methylhept-5-en-2-ylidene)cyclohexene	Terpene	495-62-5	Fruity	Balsamic, citrus, myrrh, spicy, woody, fruity, tropical, terpene, green, banana	Sweet		Honey	Fruity	Citric	Orange	[116]
48	Butyl alcohol	Butan-1-ol	Alcohols	71-36-3	Fermented	Fusel, oily, sweet, balsamic, whiskey, Malty, solvent-like	Palate	Sensations	Alcoholic	Oily		Butter	[42,48,134]
49	Butyl butyrate	Butyl butanoate	Esters	109-21-7	Fruity	Fruity, banana, pineapple, green, cherry, tropical fruit, ripe, sweet	Fruity	Tropical	Apple	Sweet		Honey	[134]
50	Butyric acid	Butanoic acid	Acid	107-92-6	Cheesy	Sharp, acetic, cheesy, buttery, fruity, dairy	Oily		Butter	Sweet		Honey	[38,48]
51	Caryophyllene	(4Z)-4,11,11-trimethyl-8-methylenebicyclo(7.2.0)undec-4-ene	Sesquiterpene	13877-93-5	Spicy	Sweet, woody, spicy, clove, dry, terpene	Spices		Clove	Sweet		Honey	[116]
52	Cis-oak lactone	(4R,5R)-5-butyl-4-methyloxolan-2-one	Lactones	55013-32-6	Spicy	Coconut, flowery, woody, sweet, spicy, coconut, vanilla	Woody		Vanilla	Woody		Resin	[2]
53	D-Dihydrogeraniol	(3R)-3,7-Dimethyloct-6-en-1-ol	Alcohols	1117-61-9	Floral	Citronella, roses, leafy, oily, petal	Floral		Roses	Fruity	Citric	Orange	[48,78,94,115]
54	Decanal	Decanal	Aldehydes	112-31-2	Aldehydic	Sweet, aldehydic, waxy, orange, peel citrus, floral	Fruity	Citric	Orange	Fruity	Red fruits	Strawberry	[116]
55	Decanoic acid	Decanoic acid	Acid	334-48-5	Fatty	Rancid, sour, fatty, citrus	Oily		Peanut	Fruity	Citric	Orange	[38,48,78,134]
56	Decanol	Decan-1-ol	Alcohols	112-30-1	Fatty	Fatty, waxy, floral, orange, sweet, clean, watery	Oily		Peanut	Fruity	Citric	Orange	[94,115]
57	Decyl acetate	Decyl acetate	Esters	112-17-4	Waxy	Waxy, clean, fresh, cloth, laundered cloth, citrus, soapy, sweet, fatty, creamy	Oily		Butter				[94,115]
58	Dextro,laevo-menthol	5-methyl-2-propan-2-ylcyclohexan-1-ol	Terpene alcohol	89-78-1	Menthol	Peppermint, cooling, woody	Vegetal		Mint	Oily		Peanut	[48,116]
59	Diacetyl	Butane-2,3-dione	Ketones	431-03-8	Buttery	Buttery, sweet, creamy, pungent, caramellike	Oily		Butter				[10,42]
60	Diethyl malate	Diethyl malate	Esters	7554-12-3	Caramellike	Caramellike, sugar, brown sugar, sweet, winey, fruity, herbal	Sweet		Caramel	Fruity	Tropical	Pear	[38]
61	Diethyl succinate	Diethyl butanedioate	Esters	123-25-1	Fruity	Fruity, apple, cooked apple, ylang	Fruity	Tropical	Apple	Sweet		Honey	[38,48,94,115,116,134]
62	Dihydroeugenol	2-Methoxy-4-propylphenol	Alcohols	2785-87-7	Spicy	Clove, sharp, spicy, sweet, phenolic, powdery, allspice	Spices		Clove	Sweet		Honey	[94,115]
63	Dimethyl sulfide	Methylsulfanylmethane	Thioether	75-18-3	Sulphurous	Sulphurous, onion, sweet corn, vegetable, cabbage, tomato, green, radish, creamy, tomato, fishy, seafood, berry, fruity	Chemical	Off flavours	Sulphurous	Vegetal		Herbal	[42]
64	Ethanol	Ethanol	Alcohols	64-17-5	Alcoholic	Alcoholic, ethereal, medicinal	Chemical	Aggressive	Ethanol				[14,48,78]
65	Ethyl (E)-2-decenoate	Ethyl (E)-dec-2-enoate	Esters	7367-88-6	Green	Green, aldehydic, fatty, watercress, herbal, waxy, melon, unripe melon, metallic, green apple, pear, fruit	Vegetal		Herbal	Fruity	Tropical	Apple	[94,115]
66	Ethyl (R)-2-hydroxy-4-methyl pentanoate	Ethyl (R)-2-hydroxy-4-methyl pentanoate	Esters	60856-83-9	Fruity	Fresh, blackberry	Fruity	Tropical	Apple	Sweet		Honey	[78]
67	Ethyl (Z)-linoleate	Ethyl (Z)-linoleate	Esters	544-35-4	Fatty	Fatty, fruity, oily	Oily		Peanut	Sweet		Honey	[78]
68	Ethyl 2-methyl butyrate	Ethyl 2-methylbutanoate	Esters	7452-79-1	Fruity	Sharp, sweet, green apple, fruity, berry, fresh, tropical	Fruity	Tropical	Apple	Sweet		Honey	[94,115]
69	Ethyl 9-decenoate	Ethyl 9-decenoate	Esters	67233-91-4	Fruity	Fruity, fatty	Sweet		Honey				[94,115,116]
70	Ethyl acetate	Ethyl acetate	Esters	141-78-6	Ethereal	Ethereal, fruity, sweet, weedy, green, grape, rummy, pineapple, solvent	Chemical	Off flavours	Ethyl acetate	Sweet		Molasses	[14,38,42,48,78,80,94,115,116,134]
71	Ethyl acrylate	Ethyl prop-2-enoate	Esters	140-88-5	Plastic	Plastic, acrylate, fruity	Chemical	Off flavours	Plastic				[10]
72	Ethyl butyrate	Ethyl butanoate	Esters	105-54-4	Fruity	Fruity, pineapple, cognac, sweet, “tutti frutti”, Ethereal, apple, buttery	Sweet		Honey	Fruity	Citric	Lemon	[10,38,42,80,94,115,116]
73	Ethyl decanoate	Ethyl decanoate	Esters	110-38-3	Waxy	Sweet, waxy, fruity, apple, grape, oily, brandy, fatty, nut	Oily		Butter				[42,78,80,94,115,116,134]
74	Ethyl formate	Ethyl formate	Esters	109-94-4	Ethereal	Ethereal, fruity, rum-like, green, alcoholic, roses, cognac, fermented, winey, cognac	Palate	Sensations	Alcoholic	Sweet		Molasses	[2]
75	Ethyl heptanoate	Ethyl heptanoate	Esters	106-30-9	Fruity	Fruity, pineapple, cognac, rummy, winey, sweet, banana, berry, green, seedy	Sweet		Honey	Fruity	Citric	Lemon	[94,115]
76	Ethyl hexanoate	Ethyl hexanoate	Esters	123-66-0	Fruity	Sweet, fruity, pineapple, waxy, green, banana	Sweet		Honey	Fruity	Citric	Lemon	[42,48,78,80,94,115,134]
77	Ethyl isobutyrate	Ethyl 2-methylpropanoate	Esters	97-62-1	Fruity	Sweet, ethereal, fruity, alcoholic, fusel, rummy, pungent	Palate	Sensations	Alcoholic	Sweet		Honey	[94,115]
78	Ethyl isovalerate	Ethyl 3-methylbutanoate	Esters	108-64-5	Fruity	Fruity, sweet, apple, pineapple, “tutti frutti”, sharp, apple, green, orange	Sweet		Honey	Fruity	Citric	Lemon	[94,115]
79	Ethyl lactate	Ethyl 2-hydroxypropanoate	Esters	97-64-3	Fruity	Sharp, tart, fruity, buttery, butterscotch, sweet, acidic, ethereal, strawberry, perfumed	Sweet		Honey	Oily		Butter	[42,78,80,134]
80	Ethyl laurate	Ethyl dodecanoate	Esters	106-33-2	Waxy	Sweet, waxy, floral, soapy, clean, rummy, creamy, oily, fatty	Oily		Butter				[42,48,78,80,116,134]
81	Ethyl myristate	Ethyl tetradecanoate	Esters	124-06-1	Waxy	Sweet, waxy, violet, orris	Oily		Butter				[48,115,116] [94,134]
82	Ethyl nonanoate	Ethyl nonanoate	Esters	123-29-5	Waxy	Fruity, roses, waxy, rummy, winey, tropical, cognac, apple, banana	Oily		Butter				[42,80,94,115,116]
83	Ethyl octanoate	Ethyl octanoate	Esters	106-32-1	Waxy	Fruity, winey, waxy, sweet, apricot, banana, brandy, pear, musty, pineapple, creamy, dairy, cognac	Oily		Butter				[38,42,48,78,80,94,115,116,134]
84	Ethyl palmitate	Ethyl hexadecanoate	Esters	628-97-7	Waxy	Waxy, fruity, creamy, milky, balsamic, greasy, oily	Oily		Butter				[116,134]
85	Ethyl pentadecanoate	Ethyl pentadecanoate	Esters	41114-00-5	Sweet	Honey, sweet	Sweet		Honey				[78]
86	Ethyl phenyl acetate	Ethyl 2-phenylacetate	Esters	101-97-3	Floral	Sweet, floral, honey, roses, balsamic, cocoa, chocolate, dark chocolate, anisic, liquorice, black liquorice.	Floral		Roses	Sweet		Honey	[10]
87	Ethyl propionate	Ethyl propanoate	Esters	105-37-3	Fruity	Sweet, fruity, rummy, grape, pineapple, ethereal, winey, fermented, egg-nog, rum-like	Sweet		Honey	Fruity	Citric	Lemon	[94,115]
88	Ethyl undecanoate	Ethyl undecanoate	Esters	627-90-7	Soapy	Soapy, waxy, fatty, cognac, coconut, buttery	Chemical	Off flavours	Soap	Oily		Butter	[94,115]
89	Ethyl valerate	Ethyl pentanoate	Esters	539-82-2	Fruity	Fruity, apple, sweet, pineapple, green, tropical, acidic, berry	Fruity	Tropical	Pineapple	Fruity	Tropical	Banana	[2]
90	Eugenol	2-methoxy-4-prop-2-enylphenol	Phenolic ether	97-53-0	Spicy	Sweet, spicy, clove, woody, phenolic, savoury, ham, bacon, cinnamon, allspice	Spices		Clove				[94,115]
91	Farnesyl acetate	3,7,11-trimethyldodeca-2,6,10-trienyl acetate	Esters	29548-30-9	Floral	green, floral, orchid, metallic, cortex, waxy, roses, citrus	Floral		Roses	Fruity	Citric	Orange	[116]
92	Fenchol	1,3,3-trimethylbicyclo[2.2.1]heptan-2-ol	Alcohols	1632-73-1	Camphoraceous	Camphoraceous, pine, woody, dry, sweet, lemon	Woody		Resin	Fruity	Citric	Lemon	[116]
93	Furfural	Furan-2-carbaldehyde	Aldehydes	98-01-1	Bready	Sweet, woody, almond, bread, baked, caramellike, phenolic	Woody		Resin	Sweet		Caramel	[38,42,134]
94	Furfuryl acetate	Furan-2-ylmethyl acetate	Esters	623-17-6	Fruity	Sweet, fruity, banana, horseradish	Sweet		Honey				[48]
95	Furfuryl alcohol	Furan-2-ylmethanol	Alcohols	98-00-0	Bready	Alcoholic, chemical, musty, sweet, caramellike, bready, coffee, sulphurous	Woody		Coffee	Sweet		Caramel	[38,48]
96	Geraniol	(2E)-3,7-dimethylocta-2,6-dien-1-ol	Alcohols	106-24-1	Floral	Sweet, floral, fruity, roses, waxy, citrus, citronella	Floral		Roses	Fruity	Citric	Lemon	[48]
97	Heptanal	Heptanal	Aldehydes	111-71-7	Green	Fresh, aldehydic, fatty, green, herbal, cognac, ozone	Vegetal		Herbal	Palate	Sensations	Alcoholic	[10,94,115]
98	Heptanoic acid	Heptanoic acid	Acid	111-14-8	Cheesy	Rancid, sour, cheesy, sweaty, waxy, fermented, pineapple, fruity	Chemical	Aggressive	Head fraction	Fruity	Tropical	Pineapple	[38,48]
99	Heptyl alcohol	1-Heptanol	Alcohols	111-70-6	Green	Musty, leafy, violet, herbal, green, sweet, woody, peony	Vegetal		Herbal	Oily		Peanut	[48]
100	Hexadecanol	Hexadecan-1-ol	Alcohols	36653-82-4	Waxy	Waxy, clean, greasy, floral, oily	Oily		Butter				[134]
101	Hexanal diethyl acetal	1,1-Diethoxyhexane	Acetal	3658-93-3	Fermented	Cognac, pear, floral, hyacinth, apple, fruity	Palate	Sensations	Alcoholic	Sweet		Honey	[94,115]
102	Hexanoic acid	Hexanoic acid	Acid	42-62-1	Fatty	Sour, fatty, sweaty, cheesy	Oily		Peanut	Chemical	Aggressive	Head fraction	[38,48,134]
103	Hexanol	Hexan-1-ol	Alcohols	111-27-3	Herbal	Ethereal, fusel, oily, fruity, alcoholic, sweet, green, pungent	Vegetal		Herbal	Palate	Sensations	Alcoholic	[48,94,115]
104	Isoamyl acetate	3-methylbutyl acetate	Esters	123-92-2	Fruity	Sweet, fruity, banana, solvent, ripe	Sweet		Honey				[10,38]
105	Isoamyl alcohol	3-methylbutan-1-ol	Esters	123-51-3	Fermented	Fusel, alcoholic, whiskey, fruity, banana, pungent, ethereal, cognac, molasses	Palate	Sensations	Alcoholic	Sweet		Honey	[14,42,48,78,134]
106	Isoamyl decanoate	3-methylbutyl decanoate	Esters	2306-91-4	Waxy	Waxy, banana, fruity, sweet, cognac, green	Oily		Butter				[78]
107	Isoamyl octanoate	3-methylbutyl octanoate	Esters	2035-99-6	Fruity	Sweet, oily, fruity, green, soapy, pineapple, coconut	Sweet		Honey	Fruity	Citric	Lemon	[42,80,94,115,134]
108	Isobutyl acetate	2-Methylpropyl acetate	Esters	110-19-0	Fruity	Sweet, fruity, ethereal, banana, tropical, apple	Sweet		Honey	Fruity	Citric	Lemon	[38,94,115]
109	Isobutyl alcohol	2-methylpropan-1-ol	Alcohols	78-83-1	Ethereal	Ethereal, winey, malty	Palate	Sensations	Alcoholic				[14,42,48,116,134]
110	Isobutyl octanoate	2-Methylpropyl octanoate	Esters	5461-06-3	Fruity	Fruity, green, oily, floral	Sweet		Honey	Floral		Roses	[94,115]
111	Isobutyraldehyde	2-methylpropanal	Aldehydes	78-84-2	Aldehydic	Fresh, aldehydic, floral, pungent, green	Floral		Roses	Woody		Resin	[42]
112	Isobutyric acid	2-methylpropanoic acid	Acid	79-31-2	Acidic	Acidic, sour, cheesy, dairy, buttery, rancid	Oily		Butter	Chemical	Aggressive	Solvent	[38]
113	Isovaleraldehyde	3-methylbutanal	Aldehydes	590-86-3	Aldehydic	Ethereal, aldehydic, chocolate, peach, fatty, green, sweet	Woody		Chocolate	Fruity	Tropical	Peach	[42]
114	Linalool	3,7-dimethylocta-1,6-dien-3-ol	Alcohols	78-70-6	Floral	Citrus, floral, sweet, bois de roses, woody, green, blueberry, orange, terpenic, waxy.	Floral		Roses	Fruity	Citric	Lemon	[38,48]
115	Methyl 2-hydroxy-4-methyl valerate	Methyl 2-hydroxy-4-methylpentanoate	Esters	40348-72-9	Fruity	Sweet, fruity, musty	Sweet		Honey				[134]
116	Methyl salicylate	Methyl 2-hydroxybenzoate	Esters	119-36-8	Minty	Wintergreen, minty, sweet, root beer, phenolic, camphoraceous	Vegetal		Mint	Vegetal		Balsamic	[2]
117	Methyl stearate	Methyl octadecanoate	Esters	112-61-8	Waxy	Oily, waxy	Oily		Butter				[134]
118	Nerolidol	3,7,11-trimethyldodeca-1,6,10-trien-3-ol	Terpene	7212-44-4	Floral	Floral, green, waxy, citrus, woody	Floral		Roses	Fruity	Citric	Lemon	[78,116]
119	Nerolidol acetate	[(6E)-3,7,11-trimethyldodeca-1,6,10-trien-3-yl] acetate	Esters	2306-78-7	Floral	Fresh, sweet, citrus, waxy, freesia, woody	Floral		Roses	Fruity	Citric	Lemon	[116]
120	Nonanal	Nonanal	Aldehydes	124-19-6	Aldehydic	Waxy, aldehydic, roses, fresh, orris, orange peel, fatty, citrus, green, lemon peel, cucumber	Floral		Roses	Fruity	Citric	Orange	[94,115]
121	Nonanal diethyl acetal	1,1-Diethoxynonane	Acetal	54815-13-3	Aldehydic	Aldehydic, floral, rose	Floral		Roses				[94,115]
122	Nonanoic acid	Nonanoic acid	Acid	112-05-0	Waxy	Waxy, cheesy, dairy	Oily		Butter				[38,48]
123	Nonanol	Nonan-1-ol	Alcohols	143-08-8	Floral	Fresh, clean, fatty, floral, roses, orange, dusty, wet, oily	Floral		Roses	Oily		Peanut	[94,115]
124	Octanal	Octanal	Aldehydes	124-13-0	Aldehydic	aldehydic, waxy, citrus, orange peel, green, herbal, fresh, fatty	Fruity	Citric	Orange	Vegetal		Herbal	[38,116]
125	Octanoic acid	Octanoic acid	Acid	124-07-2	Fatty	Fatty, waxy, rancid, oily, vegetable, cheesy	Oily		Peanut	Vegetal		Herbal	[38,48,78,134]
126	Octanol	Octan-1-ol	Alcohols	111-87-5	Waxy	Waxy, green, orange, aldehydic, roses, mushroom, citrus, floral, sweet, fatty, coconut.	Oily		Butter				[48,78,116,134]
127	Ortho-guaiacol	2-methoxyphenol	Phenolic ether	90-05-1	Phenolic	Phenolic, smoky, spicy, vanilla, woody	Chemical	Aggressive	Medicinal	Sweet		Honey	[10,38,48]
128	Palmitic acid	Hexadecanoic acid	Acid	57-10-3	Waxy	Waxy, fatty, creamy	Oily		Butter				[48]
129	Phenethyl alcohol	2-Phenylethanol	Alcohols	60-12-8	Floral	Floral, roses, dried roses, sweet, fresh, bready, honey	Floral		Roses	Sweet		Honey	[38,48,78,94,115,116,134]
130	Phenyl acetate	Phenyl acetate	Esters	122-79-2	Phenolic	Phenolic, medicinal, animal, resinous, castoreum, woody, smoky, burnt	Chemical	Aggressive	Medicinal				[48]
131	Phenylethyl acetate	2-phenylethyl acetate	Esters	103-45-7	Floral	Floral, roses, sweet, honey, fruity, tropical, yeasty, cocoa, balsamic, raspberry.	Floral		Roses	Sweet		Honey	[38,48]
132	Propionaldehyde	Propanal	Aldehydes	123-38-6	Ethereal	Earthy, alcoholic, winey, whiskey, cocoa, nutty, ethereal, pungent, cognac, brandy, meaty, grape	Palate	Sensations	Alcoholic	Spices		Anise	[42,134]
133	Propionic acid	Propanoic acid	Acid	79-09-4	Acidic	Pungent, acidic, cheesy, vinegar, dairy.	Chemical	Aggressive	Acetic	Oily		Butter	[38]
134	Propyl acetate	Propyl acetate	Esters	109-60-4	Fruity	Solvent, celery, fruity, fusel, raspberry, pear, pungent, sweet	Fruity	Red fruits	Raspberry	Sweet		Honey	[38]
135	Propyl alcohol	Propan-1-ol	Alcohols	71-23-8	Alcoholic	Alcoholic, fermented, fusel, musty, tequila, yeasty, sweet, fruity, apple, pear	Palate	Sensations	Alcoholic	Fruity	Tropical	Pear	[14,38,42,48,134]
136	Propyl decanoate	Propyl decanoate	Esters	30673-60-0	Waxy	Waxy, fruity, fatty, green, vegetable, woody, oily, fruity	Oily		Butter				[94,115]
137	Propyl octanoate	Propyl octanoate	Esters	624-13-5	Coconut	Coconut, cocoa, cognac, winey, fatty	Oily		Coconut				[94,115]
138	Valeraldehyde	Pentanal	Aldehydes	110-62-3	Fermented	Fermented, bready, fruity, nutty, berry	Palate	Sensations	Alcoholic	Sweet		Molasses	[42]
139	Valeric acid	Pentanoic acid	Acids	109-52-4	Cheesy	Strong, pungent, cheesy, acidic, sweaty, rancid, sharp, sour milky, tobacco, fruity	Chemical	Aggressive	Medicinal	Woody		Tobacco	[2]
140	Vanillin	4-hydroxy-3-methoxybenzaldehyde	Phenolic aldehyde	121-33-5	Vanilla	Sweet, vanilla, creamy, chocolate, phenolic	Sweet		Vanilla	Sweet		Chocolate	[10]
141	Vinyl amyl ketone	1-octen-3-one	Ketones	4312-99-6	Earthy	Herbal, mushroom, earthy, musty, dirty, metallic, vegetable, cabbage, broccoli, savoury, fishy, chicken	Vegetal		Herbal		Woody	Resin	[10]
142	α-Bisabolol	(2R)-6-methyl-2-[(1R)-4-methyl-1-cyclohex-3-enyl]hept-5-en-2-ol	Terpene alcohol	515-69-5	Floral	Floral, peppery, balsamic, clean	Floral		Roses	Spices		Pepper	[116]
143	γ-Nonalactone	5-pentyloxolan-2-one	Lactones	104-61-0	Coconut	Coconut, creamy, waxy, buttery, sweet, oily, fatty	Oily		Coconut	Oily		Butter	[2]
The odour type and odour descriptors were obtained from the Good Scents Company Information System [200].													

## References

[1] FGV (2017). The Brazilian Cachaca Industry and Their Interactions with International Trade. https://agro.fgv.br/publicacao/brazilian-cachaca-industry-and-their-interactions-international-trade.

[2] Bortoletto A.M., Hill A., Jack F. (2023). Chapter 3—Rum and cachaça. Distilled Spirits.

[3] De Silva A.P., Silvello G.C., Bortoletto A.M., Alcarde A.R. (2020). Composição química de aguardente de cana obtida por diferentes métodos de destilação. Braz. J. Food Technol..

[4] BRASIL (2023). Ministério da Agricultura, Pecuária e Abastecimento (MAPA). no 539, de 26 de Dezembro de 2022. https://www.in.gov.br/en/web/dou/-/portaria-mapa-n-539-de-26-de-dezembro-de-2022-453828778.

[5] Pataro C., Guerra J.B., Gomes F.C., Neves M.J., Pimentel P.F., Rosa C.A. (2002). Trehalose accumulation, invertase activity and physiological characteristics of yeasts isolated from 24 h fermentative cycles during the production of artisanal Brazilian cachaça. Braz. J. Microbiol..

[6] Haller H. (2016). Cachaça: The most famous spirit you’ve never heard on. Artisan Spirit: Spring 2016.

[7] Silva J.H.D.N., Verruma-Bernardi M.R., de Oliveira A.L. (2020). Cachaça Production in Brazil and its Main Contaminant (Ethyl Carbamate). Sci. Agric..

[8] Schwan R.F., Mendonça A.T., Da Silva J.J., Rodrigues V., Wheals A.E. (2001). Microbiology and physiology of Cachaça (Aguardente) fermentations. Antonie Van Leeuwenhoek Int. J. Gen. Mol. Microbiol..

[9] Cardoso D.R., Andrade-Sobrinho L.G., Leite-Neto A.F., Reche R.V., Isique W.D., Ferreira M.M.C., Lima-Neto B.S., Franco D.W. (2004). Comparison between Cachaça and Rum Using Pattern Recognition Methods. J. Agric. Food Chem..

[10] De Souza M.D.C.A., Vásquez P., del Mastro N.L., Acree T.E., Lavin E.H. (2006). Characterization of Cachaça and Rum Aroma. J. Agric. Food Chem..

[11] Cardoso M.D.G. (2020). Produção de Aguardente de Cana.

[12] Waldemar G.V. (2010). Bebidas Alcoólicas: Ciência e Tecnologia.

[13] Alvarez F., da Mata Correa L.F., Araújo T.M., Mota B.E.F., da Conceição L.E.F.R., de Miranda Castro I., Brandão R.L. (2014). Variable flocculation profiles of yeast strains isolated from cachaça distilleries. Int. J. Food Microbiol..

[14] Campos C., Silva C., Dias D., Basso L., Amorim H., Schwan R. (2010). Features of *Saccharomyces cerevisiaeas* a culture starter for the production of the distilled sugar cane beverage, cachaça in Brazil. J. Appl. Microbiol..

[15] Duarte W.F., de Sousa M.V.F., Dias D.R., Schwan R.F. (2011). Effect of Co-Inoculation of *Saccharomyces cerevisiae* and *Lactobacillus fermentum* on the Quality of the Distilled Sugar Cane Beverage Cachaça. J. Food Sci..

[16] Gomes F.C., Pataro C., Guerra J.B., Neves M.J., Corrêa S.R., Moreira E.S., Rosa A.C. (2002). Physiological diversity and trehalose accumulation in *Schizosaccharomyces pombe* strains isolated from spontaneous fermentations during the production of the artisanal Brazilian *cachaça*. Can. J. Microbiol..

[17] Machado A.M.d.R., Cardoso M.d.G., Saczk A.A., dos Anjos J.P., Zacaroni L.M., Dórea H.S., Nelson D.L. (2013). Determination of ethyl carbamate in cachaça produced from copper stills by HPLC. Food Chem..

[18] Portugal C.B., de Silva A.P., Bortoletto A.M., Alcarde A.R. (2017). How native yeasts may influence the chemical profile of the Brazilian spirit, cachaça?. Food Res. Int..

[19] Riachi L.G., Santos Â., Moreira R.F.A., De Maria C.A.B. (2014). A review of ethyl carbamate and polycyclic aromatic hydrocarbon contamination risk in cachaça and other Brazilian sugarcane spirits. Food Chem..

[20] Lima C.M.G., Benoso P., Pierezan M.d.O., Santana R.F., Hassemer G.d.S., da Rocha R.A., Nora F.M.D., Verruck S., Caetano D., Simal-Gandara J. (2022). A state-of-the-art review of the chemical composition of sugarcane spirits and current advances in quality control. J. Food Compos. Anal..

[21] Galinaro C.A., Cardoso D.R., Franco D.W. (2007). Profiles of Polycyclic Aromatic Hydrocarbons in Brazilian Sugar Cane Spirits: Discrimination between Cachaças Produced from Nonburned and Burned Sugar Cane Crops. J. Agric. Food Chem..

[22] Masson J., Cardoso M.d.G., Zacaroni L.M., dos Anjos J.P., Sackz A.A., Machado A.M.d.R., Nelson D.L. (2012). Determination of acrolein, ethanol, volatile acidity, and copper in different samples of sugarcane spirits. Food Sci. Technol..

[23] Bortoletto A.M., Alcarde A.R. (2015). Assessment of chemical quality of Brazilian sugar cane spirits and cachaças. Food Control.

[24] E Silva J.H.D.N., Verruma-Bernardi M.R., de Medeiros S.D.S., de Oliveira A.L. (2020). Monitoring the content of ethyl carbamate and copper in organic and conventional cachaça. Sci. Agric..

[25] Labanca R.A., Glória M.B., Afonso R.J. (2008). Determinação De carbamato De etila em aguarDentes De cana por cg-em. Quim. Nova.

[26] Menezes E.G.T., Alves J.G.L.F., Valeriano C., Guimarães I.C. (2013). Physico-chemical and sensorial evaluation of sugarcane spirits produced using distillation residue. Braz. Arch. Biol. Technol..

[27] Guerreiro T.M., Ozawa K.S., Lima E.d.O., Melo C.F.O.R., de Oliveira D.N., Triano S.P.D.N., Catharino R.R. (2018). New Approach of QuEChERS and GC-MS Triple-Quadrupole for the Determination of Ethyl Carbamate Content in Brazilian cachaças. Front. Nutr..

[28] Guerra J., Araujo R., Pataro C., Franco G., Moreira E., Mendonca-Hagler L., Rosa C. (2001). Genetic diversity of *Saccharomyces cerevisiae* strains during the 24 h fermentative cycle for the production of the artisanal Brazilian cachaca. Lett. Appl. Microbiol..

[29] Oliveira E.S., Rosa C.A., Morgano M.A., Serra G.E. (2004). Fermentation characteristics as criteria for selection of cachaça yeast. World J. Microbiol. Biotechnol..

[30] Oliveira E.S., Cardello H.M.A.B., Jeronimo E.M., Souza E.L.R., Serra G.E. (2005). The influence of different yeasts on the fermentation, composition and sensory quality of cachaça. World J. Microbiol. Biotechnol..

[31] Silva C.L.C., Rosa C.A., Oliveira E.S. (2006). Studies on the kinetic parameters for alcoholic fermentation by flocculent *Saccharomyces cerevisiae* strains and non-hydrogen sulfide-producing strains. World J. Microbiol. Biotechnol..

[32] Morais P., Rosa C., Linardi V., Pataro C., Maia A. (1997). Short Communication: Characterization and succession of yeast populations associated with spontaneous fermentations during the production of Brazilian sugar-cane aguardente. World J. Microbiol. Biotechnol..

[33] Pataro C., Guerra J., Petrillo-Peixoto M., Mendonca-Hagler L., Linardi V., Rosa C. (2000). Yeast communities and genetic polymorphism of *Saccharomyces cerevisiae* strains associated with artisanal fermentation in Brazil. J. Appl. Microbiol..

[34] Badotti F., Gomes F.C., Teodoro M.M., Silva A.L.D., Rosa C.A., Machado A.M.d.R. (2014). Electrospray Ionization Mass Spectrometry Characterization of Musts and Alembic Brazilian Cachaças Using Selected Yeast Strains. J. Food Sci..

[35] Vicente M.d.A., Fietto L.G., Castro I.d.M., dos Santos A.N.G., Coutrim M.X., Brandão R.L. (2006). Isolation of *Saccharomyces cerevisiae* strains producing higher levels of flavoring compounds for production of “cachaça” the Brazilian sugarcane spirit. Int. J. Food Microbiol..

[36] Oliveira V.A., Vicente M.A., Fietto L.G., Castro I.d.M., Coutrim M.X., Schuller D., Alves H., Casal M., Santos J.d.O., Araújo L.D. (2008). Biochemical and Molecular Characterization of *Saccharomyces cerevisiae* Strains Obtained from Sugar-Cane Juice Fermentations and Their Impact in Cachaça Production. Appl. Environ. Microbiol..

[37] Ramos C.L., Duarte W.F., Freire A.L., Dias D.R., Eleutherio E.C.A., Schwan R.F. (2013). Evaluation of stress tolerance and fermentative behavior of indigenous *Saccharomyces cerevisiae*. Braz. J. Microbiol..

[38] Duarte W.F., Amorim J.C., Schwan R.F. (2013). The effects of co-culturing non-*Saccharomyces* yeasts with *S. cerevisiae* on the sugar cane spirit (cachaça) fermentation process. Antonie Van Leeuwenhoek Int. J. Gen. Mol. Microbiol..

[39] Basso L.C., de Amorim H.V., de Oliveira A.J., Lopes M.L. (2008). Yeast selection for fuel ethanol production in Brazil. FEMS Yeast Res..

[40] Soares E. (2011). Flocculation in *Saccharomyces cerevisiae*: A review. J. Appl. Microbiol..

[41] Da Conceição L.E.F.R., Saraiva M.A.F., Diniz R.H.S., Oliveira J., Barbosa G.D., Alvarez F., Correa L.F.d.M., Mezadri H., Coutrim M.X., Afonso R.J.d.C.F. (2015). Biotechnological potential of yeast isolates from *cachaça*: The Brazilian spirit. J. Ind. Microbiol. Biotechnol..

[42] Serafim F., Franco D. (2015). Chemical traceability of industrial and natural yeasts used in the production of Brazilian sugarcane spirits. J. Food Compos. Anal..

[43] Parente D.C., Vidal E.E., Leite F.C.B., Pita W.d.B., de Morais M.A. (2015). Production of sensory compounds by means of the yeast *Dekkera bruxellensis* in different nitrogen sources with the prospect of producing cachaça. Yeast.

[44] Araújo T.M., Souza M.T., Diniz R.H.S., Yamakawa C.K., Soares L.B., Lenczak J.L., Oliveira J.V.d.C., Goldman G.H., Barbosa E.A., Campos A.C.S. (2018). Cachaça yeast strains: Alternative starters to produce beer and bioethanol. Antonie Van Leeuwenhoek Int. J. Gen. Mol. Microbiol..

[45] Costa A.C.T., Hornick J., Antunes T.F.S., Santos A.M.C., Fernandes A.A.R., Broach J.R., Fernandes P.M.B. (2021). Complete genome sequence and analysis of a *Saccharomyces cerevisiae* strain used for sugarcane spirit production. Braz. J. Microbiol..

[46] Carvalho F.P., Duarte W.F., Dias D.R., Piccoli R.H., Schwan R.F. (2014). Interaction of *Saccharomyces cerevisiae* and *Lactococcus lactis* in the fermentation and quality of artisanal cachaça. Acta Sci. Agron..

[47] Walker G.M., Stewart G.G. (2016). *Saccharomyces cerevisiae* in the Production of Fermented Beverages. Beverages.

[48] Amorim J.C., Schwan R.F., Duarte W.F. (2016). Sugar cane spirit (cachaça): Effects of mixed inoculum of yeasts on the sensory and chemical characteristics. Food Res. Int..

[49] Bernardi T.L., Pereira G.V.d.M., Cardoso P.G., Dias E.S., Schwan R.F. (2008). *Saccharomyces cerevisiae* strains associated with the production of cachaça: Identification and characterization by traditional and molecular methods (PCR, PFGE and mtDNA-RFLP). World J. Microbiol. Biotechnol..

[50] Pereira L.F., Costa C.R.L., Brasileiro B.T.R.V., de Morais M.A. (2011). *Lachancea mirantina* sp. nov., an ascomycetous yeast isolated from the cachaça fermentation process. Int. J. Syst. Evol. Microbiol..

[51] Soares T.L., Silva C.F., Schwan R.F. (2011). Monitoring the fermentation process for cachaça production using microbiological and physico-chemical methods with different *Saccharomyces cerevisiae* isolates. Food Sci. Technol..

[52] Pereira G.D.M., Ramos C., Galvão C., Dias E.S., Schwan R. (2010). Use of specific PCR primers to identify three important industrial species of Saccharomyces genus: *Saccharomyces cerevisiae*, Saccharomyces bayanus and Saccharomyces pastorianus. Lett. Appl. Microbiol..

[53] Dato M.C.F., Pizauro J.M., Mutton M.J.R. (2005). Analysis of the secondary compounds produced by *Saccharomyces cerevisiae* and wild yeast strains during the production of ‘cachaca. Braz. J. Microbiol..

[54] Barbosa E., Souza M., Diniz R., Godoy-Santos F., Faria-Oliveira F., Correa L., Alvarez F., Coutrim M., Afonso R., Castro I. (2016). Quality improvement and geographical indication of cachaça (Brazilian spirit) by using locally selected yeast strains. J. Appl. Microbiol..

[55] Jeronimo E.M., Oliveira E.d.S., Souza E.L.R., Silva M.d.A., Serra G.E. (2008). Addition of proteic nitrogen during alcoholic fermentation for the production of cachaça. Sci. Agric..

[56] Jeronimo E.M., Souza E.L.R., Silva M.D., Cruz J.C.S., Gava G.J.D., Serra G.E. (2008). Soya protein isolated in alcoholic fermentation for the production of cachaça. Bol. Do Cent. Pesqui. Process. Aliment..

[57] Mutton M.J.R., Garcia G., Teixeira V., Silva A.F., Costa G.G., Ferreira O.E. (2020). The clarification of sugarcane juice and the use of CA-11 yeast produces better quality cachaca. Rev. Ciência Agronômica.

[58] Brexó R.P., Andrietta M.d.G.S., Sant’Ana A.S. (2018). Artisanal cachaça and brewer’s spent grain as sources of yeasts with promising biotechnological properties. J. Appl. Microbiol..

[59] Ribeiro M.L.D., Ferreira O.E., Teixeira V., Mutton M.A., Mutton M.J.R. (2017). Physico-chemical treatment of sugarcane juice produces quality cachaça. Rev. Ciência Agronômica.

[60] Gonçalves R.C.F., Teodoro M.M.G., de Resende Machado A.M., Gomes F.D.C.O., Badotti F., das Graças Cardoso M. (2016). Compostos voláteis em cachaças de alambique produzidas por leveduras selecionadas e por fermentação espontânea. Magistra.

[61] Silva C.L., Vianna C.R., Cadete R.M., Santos R.O., Gomes F.C., Oliveira E.S., Rosa C.A. (2009). Selection, growth, and chemo-sensory evaluation of flocculent starter culture strains of *Saccharomyces cerevisiae* in the large-scale production of traditional Brazilian cachaça. Int. J. Food Microbiol..

[62] Araújo R.A., Gomes F.C., Moreira E.S., Cisalpino P.S., Rosa C.A. (2007). Monitoring *Saccharomyces cerevisiae* populations by mtDNA restriction analysis and other molecular typing methods during spontaneous fermentation for production of the artisanal cachaça. Braz. J. Microbiol..

[63] Gomes F., Silva C., Marini M., Oliveira E., Rosa C. (2007). Use of selected indigenous *Saccharomyces cerevisiae* strains for the production of the traditional cachaça in Brazil. J. Appl. Microbiol..

[64] De Carvalho-Netto O.V., Rosa D.D., Camargo L.E.A. (2008). Identificarion of contaminant bacteria in cachaça yeast by 16s rDNA gene sequencing. Sci. Agric..

[65] Silva P.H.A.D., Santos J.D.O., Araújo L.D., Faria F.C., Pereira A.F., Oliveira V.A.D., Vicente M.D.A., Brandão R.L. (2009). Chromatographic evaluation of volatile compounds in brazilian sugar cane spirits produced with yeasts from different locations. Ciência Tecnol. Aliment..

[66] Nova M.X.V., Schuler A.R.P., Brasileiro B.T.R.V., Morais M.A. (2009). Yeast species involved in artisanal cachaça fermentation in three stills with different technological levels in Pernambuco, Brazil. Food Microbiol..

[67] De Aquino F.W.B., Franco D.W. (2011). Formation of Dextran Deposits in Brazilian Sugar Cane Spirits. J. Agric. Food Chem..

[68] Martini C., Verruma-Bernardi M.R., Borges M., Margarido L.A.C., Ceccato-Antonini S.R. (2011). Yeast composition of sugar cane juice in relation to plant varieties and seasonality. Biosci. J..

[69] Gomes F.d.C.O., Araújo R.A.d.C., Cisalpino P.S., Moreira E.S.A., Zani C.L., Rosa C.A. (2009). Comparison between two selected *Saccharomyces cerevisiae* strains as fermentation starters in the production of traditional cachaça. Braz. Arch. Biol. Technol..

[70] De Souza A.P.G., Vicente M.d.A., Klein R.C., Fietto L.G., Coutrim M.X., Afonso R.J.d.C.F., Araújo L.D., da Silva P.H.A., Bouillet L.M., Castro I.M. (2011). Strategies to select yeast starters cultures for production of flavor compounds in cachaça fermentations. Antonie Van Leeuwenhoek Int. J. Gen. Mol. Microbiol..

[71] Gomes F.C.O., Silva C.L.C., Vianna C.R., Lacerda I.C.A., Borelli B.M., Nunes Á.C., Franco G.R., Mourão M.M., Rosa C.A. (2010). Identification of lactic acid bacteria associated with traditional cachaça fermentations. Braz. J. Microbiol..

[72] Lacerda I.C.A., Gomes F.C.O., Borelli B.M., Faria C.L.L., Franco G.R., Mourão M.M., Morais P.B., Rosa C.A. (2011). Identification of the bacterial community responsible for traditional fermentation during sour cassava starch, cachaça and minas cheese production using culture-independent 16s rRNA gene sequence analysis. Braz. J. Microbiol..

[73] Portugal C.B., Alcarde A.R., Bortoletto A.M., de Silva A.P. (2016). The role of spontaneous fermentation for the production of cachaça: A study of case. Eur. Food Res. Technol..

[74] Stroppa C.T., Alves J.G.L.F., de Figueiredo A.L.F., Castro C.C. (2009). Kinetic parameters of yeasts strains isolated from cachaça distilleries in Minas Gerais/Brazil. Cienc. Agrotecnologia.

[75] Badotti F., Vilaça S.T., Arias A., Rosa C.A., Barrio E. (2014). Two interbreeding populations of *Saccharomyces cerevisiae* strains coexist in cachaça fermentations from Brazil. FEMS Yeast Res..

[76] Brexó R.P., Brandão L.R., Chaves R.D., Castro R.J., Câmara A.A., Rosa C.A., Sant’ana A.S. (2020). Yeasts from indigenous culture for cachaça production and brewer’s spent grain: Biodiversity and phenotypic characterization for biotechnological purposes. Food Bioprod. Process..

[77] Nascimento R.F., Cardoso D.R., Dos B., Neto S.L., Franco D.W., Farias J.B. (1998). Influência do material do alambique na composição química das aguardentes de cana-de-açúcar. Quim. Nova.

[78] Nonato E.A., Carazza F., Silva F.C., Carvalho C.R., Cardeal Z.d.L. (2001). A Headspace Solid-Phase Microextraction Method for the Determination of Some Secondary Compounds of Brazilian Sugar Cane Spirits by Gas Chromatography. J. Agric. Food Chem..

[79] Vilela F.J., Cardoso M.D.G., Masson J., Anjos J.P.D. (2007). Determinação das composições físico-químicas de cachaças do sul de minas gerais e de suas misturas. Ciência Agrotecnologia.

[80] Nascimento E.S.P., Cardoso D.R., Franco D.W. (2008). Quantitative Ester Analysis in Cachaça and Distilled Spirits by Gas Chromatography−Mass Spectrometry (GC−MS). J. Agric. Food Chem..

[81] Tábua M.C.M., Santiago W.D., Magalhães M.L., Ferreira V.R.F., Brandão R.M., Teixeira M.L., Pedroso M.P., Machado A.M.d.R., Nelson D.L., Cardoso M.d.G. (2020). Identification of volatile compounds, quantification of glycerol and trace elements in distilled spirits produced in Mozambique. J. Food Sci. Technol..

[82] Labanca R.A., Glória M.B.A., Gouveia V.J.P., Afonso R.J.D.C.F. (2006). Determinação dos teores de cobre e grau alcoólico em aguardentes de cana produzidas no estado de Minas Gerais. Quim. Nova.

[83] Neves E.A., Oliveira A., Fernandes A.P., Nóbrega J.A. (2007). Simple and efficient elimination of copper(II) in sugar-cane spirits. Food Chem..

[84] Reche R.V., Franco D.W. (2009). Distinction between cachaças distilled in pot stills and in columns using chemometrics. Quim. Nova.

[85] Fernandes W.J., Cardoso M.d.G., Vilela F.J., de Morais A.R., Silva V.d.F., Nelson D.L. (2007). Physicochemical quality of a blend of domestic cachaças from the south of Minas Gerais. J. Food Compos. Anal..

[86] Santiago W.D., Cardoso M.d.G., Zacaroni L.M., Rodrigues L.M.A., Duarte F.C., Ribeiro C.d.F.e.S., Nelson D.L. (2015). Multivariate analysis for the characterization of physico-chemical profiles of cachaça produced in copper stills over a period of six years in Minas Gerais state. J. Inst. Brew..

[87] Alcarde A.R., de Souza P.A., Belluco A.E.d.S. (2010). Volatilization kinetics of secondary compounds from sugarcane spirits during double distillation in rectifying still. Sci. Agric..

[88] Silva F.A., Vendruscolo F., Carvalho W.R., Júnior M.S.S., Pinheiro M.V.M., Caliari M. (2013). Influence of the number of distillations on the composition of organic sugarcane spirit. J. Inst. Brew..

[89] Franitza L., Nicolotti L., Granvogl M., Schieberle P. (2018). Differentiation of Rums Produced from Sugar Cane Juice (Rhum Agricole) from Rums Manufactured from Sugar Cane Molasses by a Metabolomics Approach. J. Agric. Food Chem..

[90] Soares S.A.R., Costa S.S.L., Araujo O.R.G., Teixeira L.S.G., Dantas A.F. (2018). Comparison of Spectrophotometric Methods for the Determination of Copper in Sugar Cane Spirit. J. AOAC Int..

[91] Cardoso D.R., Lima-Neto B.S., Franco D.W. (2003). Influência do material do destilador na composição química das aguardentes de cana. Parte II. Quim. Nova.

[92] Cardoso D.R., Sobrinho L.G.A., Lima-Neto B.S., Franco D.W. (2004). A rapid and sensitive method for dimethylsulphide analysis in Brazilian sugar cane sugar spirits and other distilled beverages. J. Braz. Chem. Soc..

[93] Reche R.V., Neto A.F.L., Da Silva A.A., Galinaro C.A., De Osti R.Z., Franco D.W. (2007). Influence of Type of Distillation Apparatus on Chemical Profiles of Brazilian Cachaças. J. Agric. Food Chem..

[94] Cardeal Z., de Souza P., da Silva M.G., Marriott P. (2008). Comprehensive two-dimensional gas chromatography for fingerprint pattern recognition in cachaça production. Talanta.

[95] De Souza P.P., Cardeal Z.d.L., Augusti R., Morrison P., Marriott P.J. (2009). Determination of volatile compounds in Brazilian distilled cachaça by using comprehensive two-dimensional gas chromatography and effects of production pathways. J. Chromatogr. A.

[96] Carlos J., Penteado P., Masini J.C. (2009). Heterogeneidade de álcoois secundários em aguardentes brasileiras de diversas origens e processos de fabricação. Quim. Nova.

[97] Negri G., Neto J.A.R.S., Carlini E.L.d.A. (2015). Chemical Analysis of Suspected Unrecorded Alcoholic Beverages from the States of São Paulo and Minas Gerais, Brazil. J. Anal. Methods Chem..

[98] Zacaroni L.M., de Sales P.F., Cardoso M.d.G., Santiago W.D., Nelson D.L. (2017). Response surface optimization of SPME extraction conditions for the analysis of volatile compounds in Brazilian sugar cane spirits by HS-SPME-GC-MS. J. Inst. Brew..

[99] Duarte F.C., Cardoso M.D.G., Pinheiro A.C.M., Santiago W.D., de Carvalho L.L. (2012). Alterações físico-químicas e sensoriais de cachaças envelhecidas submetidas à filtragem com carvão ativado. Cienc. Tecnol. Aliment..

[100] Ferreira V.H., Hantao L.W., Poppi R.J. (2021). Use of color based chromatographic images obtained from comprehensive two-dimensional gas chromatography in authentication analyses. Talanta.

[101] Nascimento H.O.D., da Silva M.Z.F., Alexandre J.B., Vidal C.B., Carvalho T.V., Nascimento R.F.D. (2022). New HS-SPME-GC-BID method for the determination of volatile constituents in distilled beverages. Microchem. J..

[102] Oliveira R.E.d.S., Cardoso M.d.G., Santiago W.D., Barbosa R.B., Alvarenga G.F., Nelson D.L. (2020). Physicochemical parameters and volatile composition of cachaça produced in the state of Paraíba, Brasil. Res. Soc. Dev..

[103] Bortoletto A.M., Silvello G.C., Alcarde A.R. (2018). Good Manufacturing Practices, Hazard Analysis and Critical Control Point plan proposal for distilleries of cachaça. Sci. Agric..

[104] Santiago W.D., Borges C.N., Barbosa R.B., Mendonça H.A., Nelson D.L., Cardoso M.d.G. (2020). Investigação sobre cachaças brasileiras quanto a sua padronização e qualidade. Res. Soc. Dev..

[105] Lima A.D.J.B., Cardoso M.D.G., Guerreiro M.C., Pimentel F.A. (2006). Emprego do carvão ativado para remoção de cobre em cachaça. Quim. Nova.

[106] Duarte F.C., Cardoso M.D.G., Magriotis Z.M., Santiago W.D., Mendonça J.G.P., Rodrigues L.M.A. (2014). Removal of copper in cachaças using clays. Cienc. Agrotecnologia.

[107] Zacaroni L.M., Magriotis Z.M., Cardoso M.d.G., Santiago W.D., Mendonça J.G., Vieira S.S., Nelson D.L. (2015). Natural clay and commercial activated charcoal: Properties and application for the removal of copper from cachaça. Food Control.

[108] Barbosa R.B., Magriotis Z.M., Gândara A.P.A., Santiago W.D., Alvarenga G.F., Brandão R.M., Oliveira R.E.d.S., Caetano A.R.S., Nelson D.L., Cardoso M.d.G. (2022). Kinetic, thermodynamic and physical-chemical study of the removal of copper from cachaça using coconut fibers. Food Addit. Contam. Part A.

[109] De Miranda M.B., Martins N.G.S., Belluco A.E.D.S., Horii J., Alcarde A.R. (2008). Perfil físico-químico de aguardente durante envelhecimento em tonéis de carvalho Chemical profile of aguardente. Ciênc. Tecnol. Aliment..

[110] Caetano D., Lima C.M.G., Sanson A.L., Silva D.F., Hassemer G.d.S., Verruck S., Silva G.A., Afonso R.J.d.C.F., Coutrim M.X., Gregório S.R. (2021). Descriptive screening and lexicon development of non-aged artisanal cachaça sensorial profile using principal component analysis and Kohonen artificial neural networks. J. Sens. Stud..

[111] Santiago W.D., Cardoso M.d.G., Lunguinho A.d.S., Barbosa R.B., Cravo F.D., Gonçalves G.d.S., Nelson D.L. (2017). Determination of ethyl carbamate in cachaça stored in newly made oak, amburana, jatobá, balsa and peroba vats and in glass containers. J. Inst. Brew..

[112] Santiago W.D., Cardoso M.d.G., Nelson D.L. (2017). Cachaça stored in casks newly constructed of oak (*Quercus* sp.), amburana (*Amburana cearensis*), jatoba (*Hymenaeae carbouril*), balsam (*Myroxylon peruiferum*) and peroba (*Paratecoma peroba*): Alcohol content, phenol composition, colour intensity and dry extrac. J. Inst. Brew..

[113] Teixeira V., Silva A.F., de Freita C.M., de Freita L.A., Mendes F.Q., Tralli L.F., Mutton M.J.R. (2019). Using Moringa oleifera Lamarck seed extract for controlling microbial contamination when producing organic cachaça. Int. J. Food Microbiol..

[114] Bortoletto A.M., Alcarde A.R. (2013). Congeners in sugar cane spirits aged in casks of different woods. Food Chem..

[115] Cardeal Z.L., Marriott P.J. (2009). Comprehensive two-dimensional gas chromatography–mass spectrometry analysis and comparison of volatile organic compounds in Brazilian cachaça and selected spirits. Food Chem..

[116] Santiago W.D., Cardoso M.d.G., Santiago J.A., Teixeira M.L., Barbosa R.B., Zacaroni L.M., Sales P.F., Nelson D.L. (2016). Physicochemical profile and determination of volatile compounds in cachaça stored in new oak (*Quercus* sp.), amburana (*Amburana cearensis*), jatoba (*Hymenaeae carbouril*), balsam (*Myroxylon peruiferum*) and peroba (*Paratecoma peroba*) casks by SPME-GC–MS. J. Inst. Brew..

[117] Buglass A. (2010). Handbook of Alcoholic Beverages.

[118] Mosedale J., Puech J.-L. (1998). Wood maturation of distilled beverages. Trends Food Sci. Technol..

[119] Parazzi C., Arthur C.M., Lopes J.J.C., Borges M.T.M.R. (2008). Avaliação e caracterização dos principais compostos químicos da aguardente de cana-de-açúcar envelhecida em tonéis de carvalho (*Quercus* sp.). Cienc. Tecnol. Aliment..

[120] de Aquino F.W.B., Rodrigues S., Nascimento R.F.D., Casimiro A.R.S. (2006). Simultaneous determination of aging markers in sugar cane spirits. Food Chem..

[121] Alcarde A.R., de Souza P.A., Belluco A.E.d.S. (2010). Aspectos da composição química e aceitação sensorial da aguardente de cana-de-açúcar envelhecida em tonéis de diferentes madeiras. Cienc. E Tecnol. Aliment..

[122] Alcarde A.R., Souza L.M., Bortoletto A.M. (2014). Formation of volatile and maturation-related congeners during the aging of sugarcane spirit in oak barrels. J. Inst. Brew..

[123] Santiago W., Cardoso M.D.G., Zacaroni L., Anjos J.D., de Resende Machado A., Mendonça J. (2012). Perfil físico-químico e quantificação de compostos fenólicos e acroleína em aguardentes de cana-de-açúcar armazenadas em tonéis de diferentes madeiras. Científica.

[124] Carvalho D.G., Ranzan L., Trierweiler L.F., Trierweiler J.O. (2020). Determination of the concentration of total phenolic compounds in aged cachaça using two-dimensional fluorescence and mid-infrared spectroscopy. Food Chem..

[125] Castro M.C., Bortoletto A.M., Silvello G.C., Alcarde A.R. (2020). Lignin-derived phenolic compounds in cachaça aged in new barrels made from two oak species. Heliyon.

[126] Silvello G.C., Bortoletto A.M., de Castro M.C., Alcarde A.R. (2021). New approach for barrel-aged distillates classification based on maturation level and machine learning: A study of cachaça. LWT.

[127] Castro M.C., Silvello G.C., Corniani L.S., Acevedo M.S.M.S.F., Pereira A.d.A.M., Alcarde A.R. (2023). Maturation-related phenolic compounds in cachaça aged in oak barrels: Influence of reuses. Wood Sci. Technol..

[128] Cardello H.M.A.B., Boscolo M., Isique W.D., Odello L., Franco D.W., Faria J.B. (2003). Evaluation of Brazilian woods as an alternative to oak for cachaças aging. Eur. Food Res. Technol..

[129] De Souza P.P., Augusti D.V., Catharino R.R., Siebald H.G.L., Eberlin M.N., Augusti R. (2007). Differentiation of rum and Brazilian artisan cachaça via electrospray ionization mass spectrometry fingerprinting. J. Mass Spectrom..

[130] De Souza P.P., de Oliveira L.C., Catharino R.R., Eberlin M.N., Augusti D.V., Siebald H.G., Augusti R. (2009). Brazilian cachaça: “Single shot” typification of fresh alembic and industrial samples via electrospray ionization mass spectrometry fingerprinting. Food Chem..

[131] Da Silva A.A., Nascimento E.S.P.D., Cardoso D.R., Franco D.W. (2009). Coumarins and phenolic fingerprints of oak and Brazilian woods extracted by sugarcane spirit. J. Sep. Sci..

[132] Rota M.B., Piggott J.R., Faria J.B. (2013). Sensory profile and acceptability of traditional and double-distilled cachaça aged in oak casks. J. Inst. Brew..

[133] Bortoletto A.M., Corrêa A.C., Alcarde A.R. (2016). Fatty acid profile and glycerol concentration in *cachaças* aged in different wood barrels. J. Inst. Brew..

[134] Caetano D., Lima C.M.G., Sanson A.L., Silva D.F., Hassemer G.d.S., Verruck S., Gregorio S.R., da Silva G.A., Afonso R.J.d.C.F., Coutrim M.X. (2022). Chemical Fingerprint of Non-aged Artisanal Sugarcane Spirits Using Kohonen Artificial Neural Network. Food Anal. Methods.

[135] Cardoso D.R., Frederiksen A.M., da Silva A.A., Franco D.W., Skibsted L.H. (2008). Sugarcane spirit extracts of oak and Brazilian woods: Antioxidant capacity and activity. Eur. Food Res. Technol..

[136] Bernardes C.D., de Figueiredo M.C., Barbeira P.J. (2014). Developing a PLS model for determination of total phenolic content in aged cachaças. Microchem. J..

[137] Bernardes C.D., Barbeira P.J.S. (2016). Different Chemometric Methods for the Discrimination of Commercial Aged Cachaças. Food Anal. Methods.

[138] Hinojosa-Nogueira D., Pérez-Burillo S., Rufián-Henares J., de la Cueva S.P. (2020). Characterization of rums sold in Spain through their absorption spectra, furans, phenolic compounds and total antioxidant capacity. Food Chem..

[139] De Castro M.C., Bortoletto A.M., Silvello G.C., Alcarde A.R. (2021). Maturation related phenolic compounds in cachaça aged in new oak barrels. J. Inst. Brew..

[140] Campos J.O.S., de Aquino F.W.B., Nascimento R.F.D., da Costa J.G.M., De Keukeleire D., de Casimiro A.R.S. (2004). Influence and effect of thermal treatment in elaboration of regional wood extracts for cachaça. J. Food Compos. Anal..

[141] Magnani B. (2009). Estudo Comparativo das Caracteristicas Sensoriais do Rum e Da Cachaça. Sao Paulo, Brazil. https://www2.fcfar.unesp.br/Home/Pos-graduacao/AlimentoseNutricao/bruna_magnani-ME_completo.pdf.

[142] Da Silva A.A., De Keukeleire D., Cardoso D.R., Franco D.W. (2012). Multivariate analyses of UV-Vis absorption spectral data from cachaça wood extracts: A model to classify aged Brazilian cachaças according to the wood species used. Anal. Methods.

[143] Catão C.G., Paes J.B., Gomes J.P., Araújo G.T. (2011). Qualidade da madeira de cinco espécies florestais para o envelhecimento da cachaça 1 Quality of wood of five forestal species for aging of ‘ cachaça. Rev. Bras. Eng. Agrícola Ambient..

[144] Ghanem E., Afsah S., Fallah P.N., Lawrence A., LeBovidge E., Raghunathan S., Rago D., Ramirez M.A., Telles M., Winkler M. (2017). Differentiation and Identification of Cachaça Wood Extracts Using Peptide-Based Receptors and Multivariate Data Analysis. ACS Sens..

[145] Simioni S.C.C., Tovar D.M., Rodrigues J.F., de Souza V.R., Nunes C.A., Vietoris V., Pinheiro A.C.M. (2018). Temporal dominance of sensations and preferences of Brazilians and Slovakians: A cross-cultural study of *cachaças* stored with woods from the Amazon rainforest. J. Sci. Food Agric..

[146] Silvello G.C., Alcarde A.R. (2020). Experimental design and chemometric techniques applied in electronic nose analysis of wood-aged sugar cane spirit (cachaça). J. Agric. Food Res..

[147] Barbosa R.B., Santiago W.D., Alvarenga G.F., Oliveira R.E.d.S., Ferreira V.R.F., Nelson D.L., Cardoso M.d.G. (2022). Physical–Chemical Profile and Quantification of Phenolic Compounds and Polycyclic Aromatic Hydrocarbons in Cachaça Samples Aged in Oak (*Quercus* sp.) Barrels with Different Heat Treatments. Food Bioprocess Technol..

[148] Karp J.R., Hamerski F., da Silva V.R., Medeiros A.B. (2019). Membrane processing of the Brazilian spirit Cachaça. J. Inst. Brew..

[149] De Almeida J.S., Meira L.A., Dias F.D.S., Teixeira L.S.G. (2019). Magnetic solid phase microextraction using CoFe_2_O_4_ nanoparticles for determination of Cu, Cd, Pb and V in sugar cane spirit samples by energy dispersive X-ray fluorescence spectrometry. Braz. J. Anal. Chem..

[150] Boscolo M., Bezerra C.W.B., Cardoso D.R., Neto B.S.L., Franco D.W. (2000). Identification and dosage by HRGC of minor alcohols and esters in Brazilian sugar-cane spirit. J. Braz. Chem. Soc..

[151] Suomalainen H., Lehtonen M. (1979). The Production of Aroma Compounds by Yeast. J. Inst. Brew..

[152] Pino J.A., Tolle S., Gök R., Winterhalter P. (2012). Characterisation of odour-active compounds in aged rum. Food Chem..

[153] Odello L., Braceschi G.P., Seixas F.R.F., Silva A.A.D., Galinaro C.A., Franco D.W. (2009). Avaliação sensorial de cachaça. Quim. Nova.

[154] Silveira A.L., Barbeira P.J.S. (2022). A fast and low-cost approach for the discrimination of commercial aged cachaças using synchronous fluorescence spectroscopy and multivariate classification. J. Sci. Food Agric..

[155] Serafim F.A.T., da Silva A.A., Galinaro C.A., Franco D.W. (2012). Chemical profile comparison of sugarcane spirits from the same wine distilled in alembics and columns. Quim. Nova.

[156] Yang H.-F., Wang S.-L., Yu S.-J., Zeng X.-A., Sun D.W. (2014). Characterization and Semiquantitative Analysis of Volatile Compounds in Six Varieties of Sugarcane Juice. Int. J. Food Eng..

[157] Belmonte-Sánchez J.R., Gherghel S., Arrebola-Liébanas J., González R.R., Vidal J.L.M., Parkin I., Frenich A.G. (2018). Rum classification using fingerprinting analysis of volatile fraction by headspace solid phase microextraction coupled to gas chromatography-mass spectrometry. Talanta.

[158] Oliveira S., Fernandes D.D.d.S., Véras G. (2019). Overview of Analytical Techniques Associated with Pattern Recognition Methods in Sugarcane Spirits Samples. Crit. Rev. Anal. Chem..

[159] Pino J.A., Winterhalter P., Gök R., González J. (2017). Characterisation of aroma-active compounds in commercial aged rums. Acta Aliment..

[160] Medeiros A., de Matos M., Monteiro A.d.P., de Carvalho J., Soccol C. (2017). Cachaça and Rum. Current Developments in Biotechnology and Bioengineering.

[161] Faria J.B. (2012). Sugar cane spirits: Cachaça and rum production and sensory properties. Alcoholic Beverages.

[162] Dos Reis K.C., Arrizon J., Amaya-Delgado L., Gschaedler A., Schwan R.F., Silva C.F. (2018). Volatile compounds flavoring obtained from Brazilian and Mexican spirit wastes by yeasts. World J. Microbiol. Biotechnol..

[163] Mattos D.S., Margarido L.A.C., Ceccato-Antonini S.R. (2018). Influence of sugarcane variety and management on the mineral composition of vinasse from alembic cachaça. Acta Sci. Technol..

[164] Dos Santos J.F., Canettieri E.V., Souza S.A., Rodrigues R., Martínez E.A. (2019). Treatment of sugarcane vinasse from cachaça production for the obtainment of Candida utilis CCT 3469 biomass. Biochem. Eng. J..

[165] Masson J.D., Graças M.C., Vilela F.J., Pimentel F.A., De Morais A.R., Anjos J.P.D. (2007). Parâmetros físico-químicos e cromatográficos em aguardentes de cana queimada e não queimada. Ciência E Agrotecnologia.

[166] Machado A.M.d.R., Cardoso M.d.G., Emídio E.S., Prata V.d.M., Dórea H.S., dos Anjos J.P., Magriotis Z.M., Nelson D.L. (2012). Experimental Design Methodology to Optimize the Solid Phase Microextraction Procedure Prior to GC/MS Determination of Ethyl Carbamate in Samples of Homemade Cachaça. Anal. Lett..

[167] Júnior J.C.B., Mendonça R.C.S., Pereira J.M.d.A.T.K., Pereira J.A.M., Soares N.d.F.F. (2011). Ethyl-carbamate determination by gas chromatography–mass spectrometry at different stages of production of a traditional Brazilian spirit. Food Chem..

[168] European Food Safety Authority (EFSA) (2007). Ethyl carbamate and hydrocyanic acid in food and beverages—Scientific Opinion of the Panel on Contaminants. EFSA J..

[169] Lachenmeier D.W., Lima M.C., Nóbrega I.C., AP Pereira J., Kerr-Corrêa F., Kanteres F., Rehm J. (2010). Cancer risk assessment of ethyl carbamate in alcoholic beverages from Brazil with special consideration to the spirits cachaça and tiquira. BMC Cancer.

[170] Menezes H.C., Paulo B.P., Paiva M.J.N., de Barcelos S.M.R., Macedo D.F.D., Cardeal Z.L. (2015). Determination of polycyclic aromatic hydrocarbons in artisanal cachaça by DI-CF-SPME–GC/MS. Microchem. J..

[171] Bueno R., Tonin A., Poliseli C., Sinosaki N., Oliveira C., Visentainer J., Ribeiro M., Silva V., Meurer E. (2020). Two Years Monitoring of Ethyl Carbamate in Sugar Cane Spirit from Brazilian Distilleries. J. Braz. Chem. Soc..

[172] Souza R.H.Z., Cardoso M.d.G., Machado A.M.R., Santiago W.D., Pedroso M.P., Brandão R.M., Oliveira R.E.S., Barbosa R.B., Alvarenga G.F., Caetano A.R.S. (2022). Polycyclic aromatic hydrocarbons in cachaças packed in bottles of polyethylene terephthalate. J. Food Sci..

[173] Barbosa R.B., Alvarenga G.F., Ferreira V.R.F., Santiago W.D., Nelson D.L., Cardoso M.D.G. (2023). Cachaça sold in polyethylene terephthalate packaging: Determination of the physical-chemical profile, polycyclic aromatic hydrocarbons and ethyl carbamate. Ciênc. Agrotecnol..

[174] Zimmerli B., Schlatter J. (1991). Ethyl carbamate: Analytical methodology, occurrence, formation, biological activity and risk assessment. Mutat. Res. /Genet. Toxicol..

[175] Aresta M., Boscolo M., Franco D.W. (2001). Copper(II) Catalysis in Cyanide Conversion into Ethyl Carbamate in Spirits and Relevant Reactions. J. Agric. Food Chem..

[176] Galinaro C.A., Franco D.W. (2011). Formação de carbamato de etila em aguardentes recém-destiladas: Proposta para seu controle. Quim. Nova.

[177] Alcarde A.R., de Souza L.M., Bortoletto A.M. (2012). Ethyl carbamate kinetics in double distillation of sugar cane spirit. J. Inst. Brew..

[178] Borges G.B.V., Gomes F.d.C.O., Badotti F., Silva A.L.D., Machado A.M.d.R. (2014). Selected *Saccharomyces cerevisiae* yeast strains and accurate separation of distillate fractions reduce the ethyl carbamate levels in alembic cachaças. Food Control.

[179] Masson J., Cardoso M.d.G., Zacaroni L.M., dos Anjos J.P., Santiago W.D., Machado A.M.d.R., Saczk A.A., Nelson D.L. (2014). GC-MS analysis of ethyl carbamate in distilled sugar cane spirits from the northern and southern regions of Minas Gerais. J. Inst. Brew..

[180] Alvarenga G.F., Machado A.M.d.R., Barbosa R.B., Ferreira V.R.F., Santiago W.D., Teixeira M.L., Nelson D.L., Cardoso M.d.G. (2023). Correlation of the presence of acrolein with higher alcohols, glycerol, and acidity in cachaças. J. Food Sci..

[181] Cravo F.D., Santiago W.D., Lunguinho A.d.S., Barbosa R.B., Oliveira R.E.d.S., Alvarenga G.F., Santos S.D., Souza R.H.Z., de Souza E.C., de Almeida K.J. (2019). Composition of Cachaças Produced from Five Varieties of Sugarcane and the Correlation of the Presence of Dhurrin in the Cane with That of Ethyl Carbamate in the Product. Am. J. Plant Sci..

[182] D’Avila G.B., Cardoso M.d.G., Santiago W.D., Rodrigues L.M.A., da Silva B.L., Cardoso R.R., Caetano A.R.S., Ribeiro C.d.F.e.S., Nelson D.L. (2016). Quantification of ethyl carbamate in cachaça produced in different agro-industrial production systems. J. Inst. Brew..

[183] De Andrade-Sobrinho L.G., Boscolo M., Dos B., Lima-Neto S., Franco D.W. (2002). Carbamato de etila em bebidas alcoólicas (cachaça, tiquira, uísque e grapa). Quim. Nova.

[184] Sobrinho L.G.d.A., Cappelini L.T.D., da Silva A.A., Galinaro C.A., Buchviser S.F., Cardoso D.R., Franco D.W. (2008). Teores de carbamato de etila em aguardentes de cana e mandioca. Parte II. Quim. Nova.

[185] Barcelos L.V.F., Cardoso M.D.G., Vilela F.J., Anjos J.P.D. (2009). Teores de Carbamato de Etila e Outros Componentes Secundários em Diferentes Cachaças Produzidas em Três Regiões do Estado de Minas Gerais: Zona da Mata, sul de Minas e Vale do Jequitinhonha. http://mct.gov.br.

[186] Nóbrega I.C., Pereira J.A., Paiva J.E., Lachenmeier D.W. (2009). Ethyl carbamate in pot still cachaças (Brazilian sugar cane spirits): Influence of distillation and storage conditions. Food Chem..

[187] Caruso M.S.F., Nagato L.A.F., Alaburda J. (2010). Benzo(a)pireno, carbamato de etila e metanol em cachaças. Quim. Nova.

[188] Zacaroni L.M., Cardoso M.d.G., Saczk A.A., Santiago W.D., dos Anjos J.P., Masson J., Duarte F.C., Nelson D.L. (2011). Caracterização e quantificação de contaminantes em aguardentes de cana. Quim. Nova.

[189] Anjos J.P.D., Cardoso M.D.G., Saczk A.A., Zacaroni L.M., Santiago W.D., Dórea H.S., Machado A.M.D.R. (2011). Identificação do carbamato de etila durante o armazenamento da cachaça em tonel de carvalho (*Quercus* sp.) e recipiente de vidro. Quim. Nova.

[190] Chreem D.R., Riachi L.G., Moreira R.F.A., de Maria C.A.B. (2015). A study of the ethyl carbamate level in cachaça samples. Int. Food Res. J..

[191] Zacaroni L.M., Cardoso M.d.G., Santiago W.D., Gomes M.d.S., Duarte F.C., Nelson D.L. (2015). Effect of light on the concentration of ethyl carbamate in cachaça stored in glass bottles. J. Inst. Brew..

[192] Mendonça J.G.P., Cardoso M.D.G., Santiago W.D., Rodrigues L.M.A., Nelson D.L., Brandão R.M., da Silva B.L. (2016). Determination of ethyl carbamate in cachaças produced by selected yeast and spontaneous fermentation. J. Inst. Brew..

[193] Riffkin H.L., Wilson R., Howie D., Muller S.B. (1989). Ethyl carbamate formation in the production of pot still whisky. J. Inst. Brew..

[194] Battaglia R., Conacher H.B.S., Page B.D. (1990). Ethyl carbamate (urethane) in alcoholic beverages and foods: A review. Food Addit. Contam..

[195] Aylott R.I., Cochrane G.C., Leonard M.J., MacDonald L.S., MacKenzie W.M., McNeish A.S., Walker D.A. (1990). Ethyl carbamate formation in grain based spirits: Part I: Post-distillation ethyl carbamate formation in maturing grain whisky. J. Inst. Brew..

[196] Bruno S., Vaitsman D., Kunigami C., Brasil M. (2007). Influence of the distillation processes from Rio de Janeiro in the ethyl carbamate formation in Brazilian sugar cane spirits. Food Chem..

[197] Santiago W.D., Cardoso M.D.G., Duarte F.C., Saczk A.A., Nelson D.L. (2014). Ethyl carbamate in the production and aging of cachaça in oak (*Quercus* sp.) and amburana (*Amburana cearensis*) barrels. J. Inst. Brew..

[198] Galinaro C.A., Ohe T.H.K., da Silva A.C.H., da Silva S.C., Franco D.W. (2015). Cyanate as an Active Precursor of Ethyl Carbamate Formation in Sugar Cane Spirit. J. Agric. Food Chem..

[199] Rodrigues L.M.A., Cardoso M.d.G., Santiago W.D., Barbosa R.B., Santiago J.d.A., Lima L.M.Z., Nelson D.L. (2020). Organic contaminants in distilled sugar cane spirits produced by column and copper alembic distillation. Res. Soc. Dev..

[200] The Good Scents Company. http://www.thegoodscentscompany.com/.

